# Improved Approach for ab Initio Calculations of Rate Coefficients for Secondary Reactions in Acrylate Free-Radical Polymerization

**DOI:** 10.3390/polym16070872

**Published:** 2024-03-22

**Authors:** Fernando A. Lugo, Mariya Edeleva, Paul H. M. Van Steenberge, Maarten K. Sabbe

**Affiliations:** 1 Laboratory for Chemical Technology (LCT), Department of Materials, Textiles, and Chemical Engineering, Ghent University, Technologiepark-Zwijnaarde 125, 9052 Ghent, Belgium; fernando.lugo@ugent.be (F.A.L.); paul.vansteenberge@ugent.be (P.H.M.V.S.); 2 Center for Polymer and Material Technology (CPMT), Department of Materials, Textiles, and Chemical Engineering, Ghent University, Technologiepark-Zwijnaarde 130, 9052 Ghent, Belgium; mariya.edeleva@ugent.be

**Keywords:** kinetics of radical polymerization, acrylates, secondary reactions, ab initio, kinetic Monte Carlo, pulsed-laser polymerization

## Abstract

Secondary reactions in radical polymerization pose a challenge when creating kinetic models for predicting polymer structures. Despite the high impact of these reactions in the polymer structure, their effects are difficult to isolate and measure to produce kinetic data. To this end, we used solvation-corrected M06-2X/6-311+G(d,p) ab initio calculations to predict a complete and consistent data set of intrinsic rate coefficients of the secondary reactions in acrylate radical polymerization, including backbiting, β-scission, radical migration, macromonomer propagation, mid-chain radical propagation, chain transfer to monomer and chain transfer to polymer. Two new approaches towards computationally predicting rate coefficients for secondary reactions are proposed: (*i*) explicit accounting for all possible enantiomers for reactions involving optically active centers; (*ii*) imposing reduced flexibility if the reaction center is in the middle of the polymer chain. The accuracy and reliability of the ab initio predictions were benchmarked against experimental data via kinetic Monte Carlo simulations under three sufficiently different experimental conditions: a high-frequency modulated polymerization process in the transient regime, a low-frequency modulated process in the sliding regime at both low and high temperatures and a degradation process in the absence of free monomers. The complete and consistent ab initio data set compiled in this work predicts a good agreement when benchmarked via *k*MC simulations against experimental data, which is a technique never used before for computational chemistry. The simulation results show that these two newly proposed approaches are promising for bridging the gap between experimental and computational chemistry methods in polymer reaction engineering.

## 1. Introduction

Polyacrylates find a wide range of applications, being important ingredients in coatings, paints, casts, adhesives, 3D printing resins, hydrogels, etc. [1,2,3,4,5,6,7,8], requiring control over the material properties (i.e., transparency, gloss, haziness, curing temperature and glass transition temperature). The wide range of applications has, in recent years, increased the interest in deeply understanding the kinetic parameters of acrylates [9,10]. These properties are determined by the molecular properties of the polymer chains, (e.g., the average molar mass and molar mass distribution, unsaturation (i.e., terminal-double-bond) level, and branching density) [11,12].

**Figure 1:**
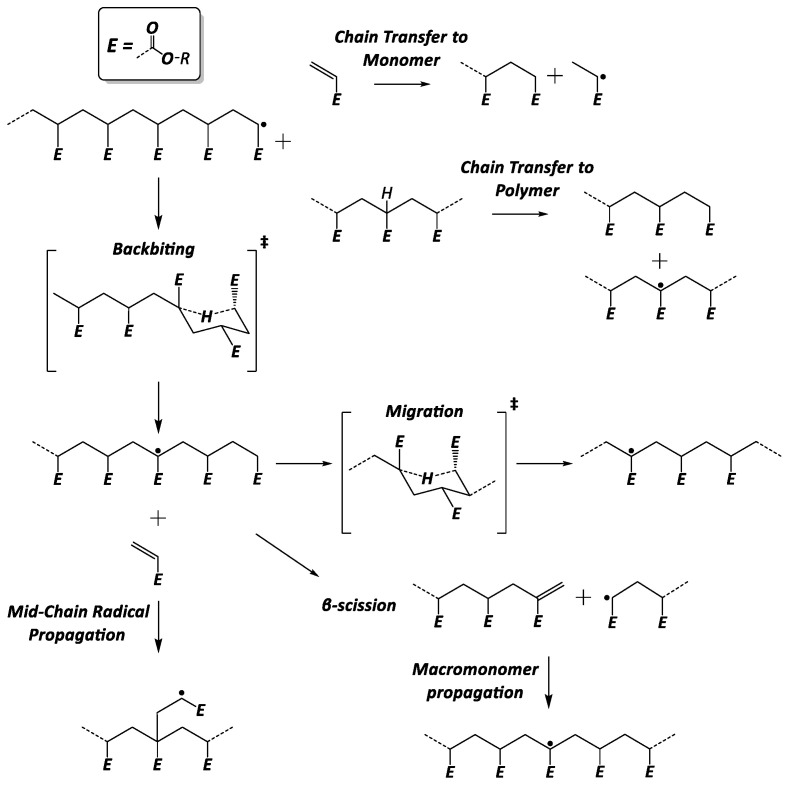
Most relevant secondary reactions in free-radical polymerization of acrylates.

Due to the complex reaction mechanism of acrylate free-radical polymerization (FRP) (i.e., the presence of numerous secondary reactions (Figure 1)), the relation between the molecular structure of the acrylic polymers and reaction conditions is not straightforward. The active radical center of an alkyl acrylate polymer chain, a so-called end-chain radical (ECR), can undergo intramolecular chain transfer reactions (backbiting), which will transfer the radical center towards the middle of the chain, forming a mid-chain radical (MCR). This tertiary carbon-centered radical has a different chemical reactivity than its secondary radical counterpart: the ability to migrate the radical center through the backbone; chain breaking through a β-scission reaction; or further propagation at a slower rate. This last reaction produces branches in the polymer, which further complicates the structural analysis of the polymer product. The β-scission reaction reduces the average chain length and produces unsaturated polymer chains (macromonomers). The latter can propagate with another ECR or MCR, bonding longer chains and creating a branch. ECRs or MCRs can also undergo intermolecular chain transfer reactions, terminating a chain by abstracting a hydrogen atom from another chain and, hence, creating a new MCR in a random position in the polymer backbone. Additionally, if the reaction proceeds with a hydrogen atom of a monomer, the chain transfer reaction produces a new unimer radical, which will start a new chain. These rather rare reaction events during polymerization significantly affect the microstructure of the polymer chains [13] and consequently they determine the material properties. As a result, the design of the tailored material properties requires a profound knowledge of these rare secondary-reaction events and accurate values of their rate coefficients.

Precise measurement of the rate coefficients in FRP reactions is challenging. For the $k_{p}$ of propagation reactions, the IUPAC Working Party on “Modelling of kinetics and processes of polymerization” proposed the use of pulsed-laser polymerization (PLP) combined with molar mass distribution (MMD) analysis as a reliable methodology [14,15,16,17,18]. This method was applied to multiple monomer types by multiple research groups, producing a set of benchmark values for monomers as styrene, (meth)acrylic esters, including linear and cyclic alkyl substituents [6,17,18,19,20,21,22,23,24,25]. However, the applicability of PLP for secondary reactions is not straightforward.

Despite the advances in measuring techniques, the effect of secondary reactions on the MMD is still difficult to isolate from the rest of the reactions. Some research relies on the analysis of the polymers’ molecular properties (branching degree, unsaturation level, *M*_n_ and MMD) to derive the values of the rate coefficients for the secondary reactions [26,27,28,29,30,31,32,33]. To analyze these properties, final-microstructure analysis methods, such as ^13^C NMR, are useful for discerning the effects of the secondary reactions in the microstructure. However, each of these molecular properties is a consequence of a set of serial or parallel secondary reactions, making it difficult to link the structural observations directly to individual rate coefficients. For example, in acrylate radical polymerization, both backbiting and chain-transfer-to-polymer reactions create MCRs. These can propagate to form branches or undergo β-scission and split the chain in two. Therefore, the degree of branching cannot be directly attributed to a single rate coefficient.

To overcome this challenge of data extraction from experiments, computational methods are used to aid the analysis of the post-processed results. For example, kinetic Monte Carlo (*k*MC) methods can simulate polymer properties, such as the MMD, allowing for more detailed comparisons with the measured experimental data. In this way, the *k*MC method allows for identifying the effects of a particular secondary reaction on the polymer product by fitting simulations and experiments under specific reaction conditions. The reliability of determining the rate coefficients through comparison to experimental data via these simulations strongly depends on the sensitivity towards the rate coefficients. It has been shown that acrylate polymers properties are sensitive to several rate coefficients at the same time [13] and, consequently, the reliable determination of individual rate coefficients is a complicated matter.

First-principle methods allow for the prediction of the rate coefficients for elementary reaction steps, which is especially useful in cases where the experimental determination is not straightforward. FRP reactions have already been studied using ab initio methods by several authors [34,35,36,37,38,39] for propagation, as well as secondary reactions. Ab initio research on secondary reactions typically focuses on which type of model/basis set combination is most suitable to represent the reaction barriers, and on the size of the model molecule. However, most rate coefficients obtained via ab initio calculations span across a very wide range. For example, the backbiting reactivity varies over multiple orders of magnitude depending on the method and basis set and cannot be considered to be in acceptable agreement with the known experimental data. A direct comparison of ab initio and experimentally derived rate coefficients is not always straightforward, as first-principle rate coefficients always represent an individual elementary reaction, while experimentally derived rate coefficients can incorporate contributions from neglected reactions or be strongly cross correlated to other rate coefficients or other phenomena (e.g., diffusional limitations, which mask or disguise the intrinsic rate coefficient) [40].

The accuracy of ab initio predictions depends on the correct construction of the molecular model. A representative ab initio model starts by selecting an adequate molecular-structure representation. The molecular structure of this model should not be as large as actual polymers because of computational limitations, while an overly small model will not account for the steric and electronic effects imposed by long chains. Therefore, the molecular model is typically made as large as can be managed with a given computational method. Furthermore, FRP reactions occur in a condensed phase in which the solvation of the radicals highly impacts the secondary reactions. By default, ab initio methods consider their rate coefficient predictions in the gas phase, which incorrectly describes the reality. Hence, the addition of solvation effects by some additional model is required, such as PCM or COSMO-RS [41]. Because the size of the molecular model is significantly smaller than a real polymer molecule, the effects that arise from the macromolecular nature of polymers, such as the size of the molecule, tacticity and 3D folding of the polymer chain, need to be accounted for through different approaches. Firstly, the acrylate polymer backbone is atactic in the case of FRP of acrylates because the repeating units can be arranged in different manners depending on the chiralities of the substituents. Secondly, the segments in the middle of the polymer chain experience restricted motion, which affects the reactivity of MCRs. 

In this work, we develop a complete set of rate coefficients of the secondary reactions in FRP of alkyl acrylates based on ab initio calculations, using the same computational level for all reactions to maintain a consistent model. We account for the solvation effects via the COSMO-RS theory. We propose two new approaches that reduce the gap between the predicted and experimental rate coefficients. The first approach accounts for all permutations of an atactic polymer chain by considering all optical isomers of the chain segment model for those reactions that involve considerable movements in the backbone of the chain. The second approach considers the flexibility reduction of a segment in a large polymer chain. We account for this by applying a geometry restriction to the model component’s molecular structure to simulate the strain applied by the rest of the backbone. To demonstrate that both approaches are efficient, the resulting ab initio data set was tested by performing *k*MC simulations of (i) pulsed-laser polymerization–size exclusion chromatography (PLP-SEC) traces under three highly different experimental conditions, and (ii) a controlled degradation of ARGET ATRP polymers. We selected a broad range of temperatures, monomer concentrations and laser frequencies to achieve a high sensitivity towards a particular secondary-reaction rate coefficient in each simulation.

## 2. Computational Details

### 2.1. Level of Theory

Ab initio calculations were performed using the Gaussian 16_C.01 package [42]. All structures were optimized at the B3LYP/6-311+G(d,p) level. Thermal contributions were calculated using the harmonic-oscillator approach at the same computational level. All structures were confirmed to have zero imaginary frequencies in the case of reactants and products, and exactly one in the transition states. Electronic energies for all stationary points were determined using single-point M06-2X/6-311+G(d,p) calculations using the previously optimized B3LYP/6-311+G(d,p) geometries. Standard ideal gas statistical thermodynamics were used to calculate the enthalpies, entropies and Gibbs energies in the gas phase [43]. The optimized geometries used to calculate every reaction within this work are included in Supporting Information.

To include solvation effects, the Gibbs energies of solvation ($\Delta G_{solv}$) were calculated using the COSMO-RS theory [44,45,46] as implemented in the COSMOtherm software package [47]. These solvation energies were calculated by COSMO-RS using BP86/TZVP single-point calculations based on the same B3LYP/6-311+G(d,p) geometry optimized before, using a methyl acrylate as the solvent with a density of 950 g L^−1^ at 298.15 K. The effect of the solvation energy on the rate coefficients is shown in Table S6 in Supporting Information. Afterwards, the $\Delta G_{solv}$ was included in the gas Gibbs energy with the following equation:

(1) $$G_{cond}^{{}^{\circ}}=G_{gas}^{{}^{\circ}}+\Delta G_{solv}$$

Classical transition state theory was used to calculate the rate coefficients based on the Gibbs reaction barrier in the condensed phase via the following equation:

(2) $$k\left(T\right)=\kappa \left(T\right)\frac{k_{B}T}{h}{\left(C^{0}\right)}^{-\Delta^{‡}n}exp\left(-\frac{\Delta^{‡}G_{cond}^{0}}{RT}\right)$$

where $\kappa \left(T\right)$ is the quantum-tunneling correction factor calculated through the Eckart method [48]; $k_{B}$ is the Boltzmann constant; h is the Plank constant; $C^{0}$ is the standard concentration of 1 mol L^−1^; $\Delta^{‡}n$ is the difference in moles between the reactant and transition states; $\Delta^{‡}G_{cond}^{0}$ is the temperature-dependent Gibbs energy barrier; and *R* is the universal gas constant. Lastly, Arrhenius parameters were regressed over a broad temperature range starting at 298.15 K till 413.15 K, as used in the simulations explained in the following section.

### 2.2. Molecular-Model Construction Considering Chiral Effects

To account for the influence of the polymer chain chirality, we predicted the reaction rate coefficient for each chiral permutation within the chosen polymer section and then averaged out the rate coefficients, including the weighting factors. For this purpose, the number of enantiomers within a molecular model must be clearly defined based on the number of chiral centers. Each radical molecular model with *n* units has *n* − 1 chiral centers, considering that the unit possessing the radical lacks chirality. Then, the molecular model has several possible enantiomers equal to all chiral permutations, excluding mirror-image structures. For example, if an ECR molecular model possesses three units, the first two units possess chirality, and the last one does not because it has a radical. Hence, the structure has two possible chiral permutations: RRM*/SSM* and RSM*/SRM*. Afterwards, the rate coefficients for all chiral permutations are averaged out using weighting factors based on chiral propagation probabilities, which will be explained in detail in Section 3.1. These rules apply for all molecules, with a few exceptions, which will be explicitly explained.

### 2.3. Reduced-Flexibility Approach

Firstly, the molecular structures of the reactants, transition state and products were optimized. Secondly, a strain was applied on the reactant and transition-state chains by increasing the distance between the first and last carbon atoms in 0.2 Å steps. This process gradually increased the electronic energies of both the reactant and transition states, which impacted the Gibbs energy of each structure. Lastly, the Gibbs energy was recalculated at each step, and the reaction barrier trend was studied so that the effect induced by the reduced-flexibility effect could be distinguished.

### 2.4. Simulation Details

The kinetic Monte Carlo (*k*MC) modeling of the pulsed-laser polymerization–size exclusion chromatography (PLP-SEC) of *n*BuA was performed using the models reported by Marien et al. [49] and Vir et al. [50], starting from the well-established Gillespie algorithm [51] combined with tree-based data structures [52], advanced sampling algorithms [53], detailed reaction schemes and well-established experimentally determined rate coefficients. The modeling details are provided by Marien et al. [49] and Vir et al. [50] We simulated 3 sets of the PLP-SEC experimental conditions, which are sensitive to the values of backbiting and β-scission secondary reactions: (1) 306 K in bulk performed at a 500 Hz laser frequency, (2) 306 K with a solvent fraction (*Φ*_s_) of 0.75 and a 50 Hz laser frequency and (3) 413 K in bulk performed at a 10 Hz laser frequency, being sensitive to ECR propagation, backbiting and β-scission, respectively.

Electron spray ionization mass spectrometry (ESI-MS) data for the synthesis of macromonomers (MMs) via the activation of bromine-capped poly(*n*-butyl acrylate) were simulated using the data set predicted in this work, including backbiting, radical migration, β-scission, macromonomer propagation and chain transfer to polymer. The rest of the model parameters were taken from Van Steenberge et al. [54] Because this experiment was conducted free of monomers in the mixture, the results were insensitive to reactions involving monomers, enhancing the effect of the aforementioned side reactions used in this simulation.

## 3. Results and Discussion

To develop the complete rate coefficient data set, each relevant reaction in the acrylate radical polymerization had to be accounted for. This included ECR propagation, backbiting or intramolecular chain transfer, β-scission, migration, macromonomer propagation, MCR propagation, chain transfer to monomer and, lastly, chain transfer to polymer or intermolecular chain transfer. For each reaction, a suitable molecular model was defined, and then ab initio predictions were performed and compared with the literature data. For every reaction, we used a specific molecular model for the ab initio calculations to achieve the maximal accuracy and minimal computational time. The relevance of the application of different molecular models arises from the structural factors, which affect a particular reaction. For example, the ECR propagation of acrylates is influenced by the molecular structure of the monomer and not by optical isomerism. Thus, we can use a simplistic model made up of two units instead of a more time-consuming larger model. At the same time, to simulate backbiting, we need to explicitly account for at least five monomer units to correctly represent the chiral effects of nearby units. Consequently, variation in the molecular model allows us to reduce the computational time without losing the accuracy of the predictions. To guide the reader, the molecular models are specified in the section corresponding to each secondary reaction. Finally, we applied *k*MC simulations to show that the set of rate coefficients derived via our improved methodology, which is a benchmark procedure never used before, provides an adequate representation of the experimental results.

### 3.1. Backbiting in Atactic Polymer Chains

**Figure 2:**
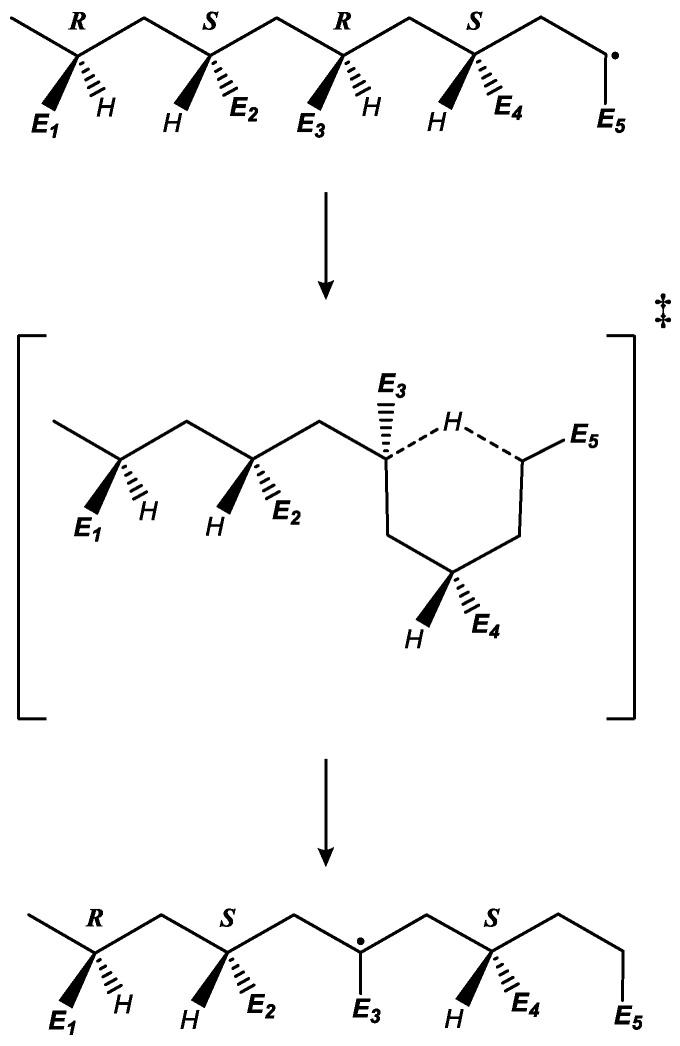
Backbiting reaction of the methyl acrylate pentamer: minimum-energy optical isomer (RSRS/SRSR).

The default approach in quantum chemistry is to calculate rate coefficients based on the minimum-energy structures of the reactant and transition states; however, this strategy is insufficient to predict backbiting rate coefficients. We realized, by testing different optical isomers for the methyl acrylate polymer chain, that the reaction barrier is strongly dependent on the tacticity of the chosen model. In the case of methyl acrylate, the pentamer model radical used has 16 possible different optical isomers due to the existence of four different chiral centers in the main chain, as shown in Figure 2.

A careful study of all the chiral permutations of these optical isomers reveals that the minimal-energy isomer corresponds to the structure with alternating chiralities (RSRS/SRSR), as this arrangement minimizes the lateral acrylate side-chain interactions. Nevertheless, the Gibbs energies in the condensed phase of the various optical isomers are, at most, 11 kJ mol^−1^ more than the minimum-energy isomer (see Supporting Information, Table S1, for all optical isomers), and therefore these other optical isomers are thermodynamically accessible as well. Although the energies indicate that these different optical isomers can exist, the actual chiral composition of the polymer chains is controlled by the propagation kinetics.

**Figure 3:**
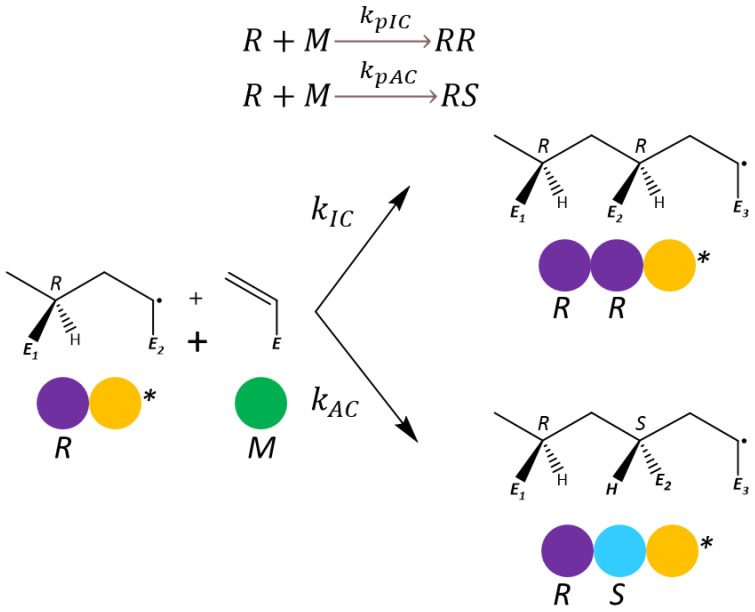
ECR propagation scheme showing identical-chirality (IC) propagation, producing a *meso* dyad, and alternating-chirality (AC) propagation, producing a *racemo* dyad.

During the propagation of an ECR in acrylate polymerization, the achiral radical center turns into a chiral carbon atom. Depending on the formed chirality, the addition could follow two pathways: in the first, it produces a chiral center with the same chirality as the preceding monomer unit in the polymer chain (*meso dyad*), and, in the second, it produces a chiral atom with a different chirality than the preceding monomer unit (*racemo dyad*). For example, a dimer radical is formed by two units: one with a chiral center (R) and the other being an ECR. After the addition of the radical to a monomer, the resulting trimer radical can have a chirality of RR or RS, as shown in the following equation and the scheme in Figure 3.

In this equation, the rate coefficient for identical-chirality propagation is represented by $k_{pIC}$, and its counterpart, alternating-chirality propagation, is represented by $k_{pAC}$. The relation between these two rate coefficients and the overall propagation rate determines the probability of the next monomer addition to create a *meso* ($P_{IC}$) or *racemo* ($P_{AC}$) dyad, as defined in Equation (3):

(3) $$P_{IC}=\frac{k_{pIC}}{k_{pIC}+k_{pAC}}\;\;\;\;\;\;\;\;\;\;\;\;P_{AC}=\frac{k_{pAC}}{k_{pIC}+k_{pAC}}$$

These rate coefficients were predicted by using a dimer model, and the results are shown in Table S2 of Supporting Information. The addition probabilities are analogous to the tacticity of the polymer because the chirality determines the relative orientation of the acrylate groups around the polymer backbone. Experimental measurements found that methyl acrylate has an almost atactic structure with a slight preference towards the *meso* configuration [55] (Table 1), which translates to a slight preference for identical-chirality sequences. This work predicts a preference towards alternating chirality, implying more *racemo* combinations than experiments predict at a relation of 2:1. However, this preference does not indicate that the acrylate polymer is not atactic because the substituents are still disposed in a random manner. Moreover, the ab initio Gibbs energy reaction barriers between identical- and alternating-chirality propagations differ only by 1.6 kJ mol^−1^ at room temperature (see Supporting Information, Table S2), which, in terms of the ab initio accuracy, means that both reaction barriers are considered equivalent.

By knowing the probability to form an identical or alternating chiral center in the backbone upon addition, it is possible to calculate the probability that an n-length polymer segment exists within the backbone by multiplying the probabilities. As an example, a segment within the backbone composed by three units that are a *meso* and then a *racemo* dyad has a probability of existence of $P_{IC}\times P_{AC}$. This enables us to calculate the chiral composition of the polymer supposing a fixed-length polymer segment, which is useful for constructing complete ab initio molecular models.

For the backbiting molecular model, we calculated the chiral composition of each possible pentamer structure based on the addition chiral probabilities for each of the 16 different enantiomers. Table 2 includes the chiral composition based on our ab initio predictions and those reported in the literature based on experiments [55]. To have a consistent relation between the chirality of the atoms of the backbone in different molecular models, the atom chirality was determined assuming that the pentamer model structure is embedded in a long polymer chain (i.e., assuming the first unit in the pentamer model (unit 1 in Figure 2)). In the backbiting reaction of the methyl acrylate pentamer, the minimum-energy optical isomer (RSRS/SRSR) is attached to the rest of the chain. With this assumption, the priority order for the chirality determination considers the left-side backbone in Figure 1 as a higher priority in contrast to the right-side backbone, resulting in R or S carbon atoms, depending on the disposition of the acrylate substituent (see Supporting Information, Figure S1, for an explicit example of determining the chiralities in the pentamer model radical). As a result, this assignment of chiralities implies that an RRRR or SSSS sequence is isotactic and that an RSRS or SRSR sequence is syndiotactic.

The statistical occurrences of the chiral sequences shown in Table 2 illustrate that no singular chiral sequence dominates the overall mixture for more than 30%. This observation remains consistent whether derived from the experimental tacticity or the computational probabilities calculated ab initio. The dominant configuration predicted in this study, namely, the RSRS/SRSR sequence, accounts for less than 30% of the sequences, while the most probable configuration identified through the experimental data, the RRRR/SSSS sequence, comprises less than 15% of the sequences. While these results may seem rather discrepant to the general reader, it must be stressed that this is the first time that these optical isomers have been explicitly calculated ab initio. These results underscore that the polymer exhibits a diverse enantiomeric composition rather than being accurately represented by a single-structure ab initio molecular model. It is evident that considering multiple configurations becomes pertinent in understanding the polymer’s microstructure, while relying on a singular model might fail to capture the inherent complexity of the polymer’s chiral composition, especially when the enantiomers display distinct reactivities.

Depending on the chirality sequence, we indeed found that the pentamer optical isomers display different backbiting reactivities, as shown in Table 3, in which the rate coefficient changes depending on the backbone chirality sequence. The arrangement of the side chains influences the energetics of the reactant radical and six-ring transition state for the backbiting. In general, isomers with alternating chiralities have higher stabilities, leading to higher reaction barriers for backbiting in comparison to isomers with identical tacticity sequences, which are typically less stable and therefore have lower backbiting barriers, as shown in Table S1. All these observations lead us to consider the reactivity of each statistically and energetically relevant isomer, rather than considering only the minimal-energy isomer, to calculate the rate coefficient. As such, the rate coefficient is calculated as the average of each optical isomer rate coefficient, weighted by the statistical occurrence of each optical isomer (see Equation (4) and Table 2). In Equation (4), ${\bar{k}}_{\mathrm{BB}}$ represents the weighted-average backbiting rate coefficient, $\omega_{i}$ represents the weight for each optical isomer (i), $k_{i}$ is the respective rate coefficient of the isomer (i) and *n* is the total number of optical isomers, which depends on the size and number of chiral atoms in the molecular model. In the backbiting case in which the model is a pentamer with four chiral atoms, which produce eight relevant optical isomers, *n* is equal to 8:

(4) $${\bar{k}}_{BB}=\;\overset{n=8}{\underset{i=1}{\sum }}\omega_{i}k_{i}$$

The obtained weighted-average rate coefficient (${\bar{k}}_{\mathrm{BB}}$) is calculated from the rate coefficients of the individual optical isomers along with the individual rate coefficient ($k_{BB}$) for each optical isomer, as reported in Table 3. The data in Table 3 show that the difference between the rate coefficients for optical isomers could be up to two orders of magnitude. Therefore, if only one of the chiral permutations is chosen as a model radical to calculate the backbiting rate coefficient, it would be an incorrect description of the average rate coefficient for an actual mixture of ECRs. The weighted-average approach provides a better overall representation of the different rates at which backbiting occurs.

The main source for the differences in the rate coefficients (Table 3) is related to the steric hindrance between the side chains of the pentamer model. The energy of these large structures is mostly dependent on the arrangement of methyl acrylate units relative to each other, and it is a direct consequence of the tacticity of the main chain. In the reactants (pentamer ECRs), the main-chain energy is minimized in a straight zig-zag shape. However, if the chirality is identical in two sequential units (*meso dyad*), then the steric repulsion between the side chains makes it energetically more favorable to bend the main chain of the polymer so that the distance between the acrylate units is increased. This will result in a curled structure, which is more likely to undergo backbiting in comparison with its counterpart with the alternating-chirality structure (*racemo dyad*) because this chain will be straighter (see Supporting Information, Figure S2, for a detailed example). This curled structure is mentioned by Yu et al. [56] in their ab initio work as an intermediate state before the backbiting reaction. Furthermore, it is important to note that the tacticity also affects the formation of the transition state in diverse ways for each optical isomer. Otherwise, the reactivities would be equal for each optical isomer despite the relative reactant stability (see Supporting Information, Table S1). The relative differences between the transition states of optical isomers arise from two sources: the arrangement of the acrylate units within the ring (third, fourth and fifth carbon atoms in Figure 2), which vary between an axial or equatorial placement, and the shape of the remainder of the chain that is not part of the ring, related to the first and second monomer units shown in Figure 2. For a visual model representation of these structures, see Supporting Information, Figure S3.

**Figure 4:**
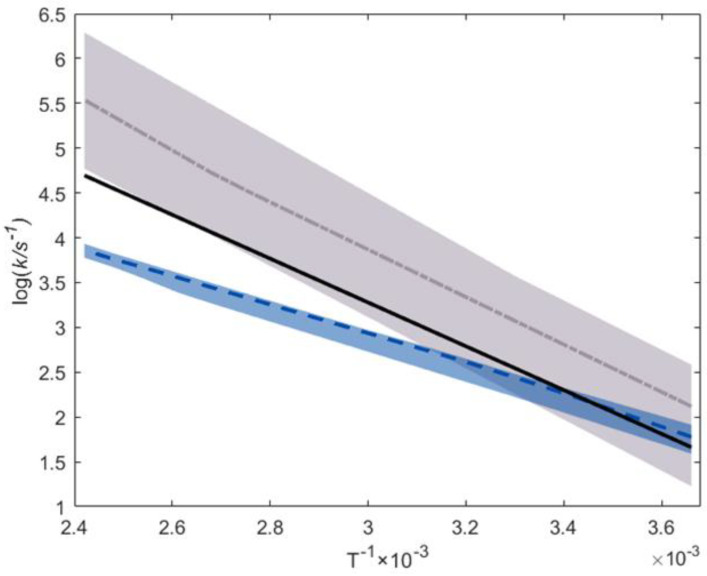
Comparison of the rate coefficients in this work with the literature values for the backbiting reaction. The colored sections represent the area between the 25% and 75% percentiles within a group of data: 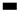 this work’s prediction; 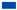 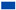 experimental median; 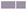 ab initio median; 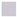 25–75% ab initio percentile area [34,35,56,62]; 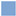 25–75% experimental percentile area [28,32,50,57,58,59,60,61].

Figure 4 compares our ab initio Arrhenius parameter prediction (black line) to the available literature data: the experimental median is the blue dashed line [28,32,50,57,58,59,60,61], the median is the grey dashed line, 25–75% of the experimental percentile zone is in light blue and the corresponding ab initio results are in light grey [34,35,56,62]. Figure 4 shows that our predictions are much closer to the experimental values than most reported ab initio predictions. Especially in the low-temperature range, our ab initio predictions agree almost perfectly with the experimentally measured values. However, there is no close agreement between our predicted values and the literature-based measured values in the high-temperature region. The higher activation energies predicted ab initio results in higher rate coefficients than the experiments indicate. Nevertheless, because all experimental works regarding alkyl acrylates are performed in the low-temperature range (273–333 K), these might be influenced by a strong tunneling effect (included in this work), which is negligible at high temperatures. Hamzehlou et al. [32] is the only experimental work focusing on high-temperature measurements, and their results produce a higher activation energy than the low-temperature measurements. Hence, data comparison is complicated because the extrapolation of low-temperature to high-temperature data should be performed accounting for the tunneling effect, which is not typically the case in the experimental literature.

### 3.2. β-Scission

**Figure 5:**
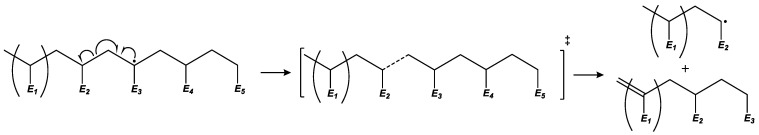
β-scission molecular model representing a left β-scission reaction.

After backbiting or migration, the MCR formed can undergo a β-scission reaction, producing an ECR and a macromonomer. Depending on which bond around the MCR breaks, the reaction is called left or right β-scission. We chose a pentamer model to calculate the rate coefficient of the β-scission and took into account the influence of the monomer units adjacent to the MCR correctly (Figure 5). Our molecular model represents a left β-scission. It has been previously shown that the barriers for left and right β-scission are comparable [34]. The β-scission reaction is one of the most highly activated reactions in most polymerization models. A β-scission reaction similar to the used model, calculated via ab initio group additive values, shows a gas-phase activation energy around 100.3 kJ mol^−1^ [63]. 

Based on the pentamer model, we predicted the following Arrhenius parameters for the left β-scission of the methyl acrylate MCR:

(5) $$k_{\beta }=\left(3.35\;\times 10^{13}\;s^{-1}\right)\exp\left(-\frac{115.97\;kJ}{RT}\right)$$

Our calculations resulted in a rate coefficient lower than those of any experimental results published [27,30,31,32,50,61]. However, it is in accordance with ab initio data published using similar methods [34,35,62], although no work before has predicted this rate coefficient using a basis set so large. A more detailed comparison of experimental and ab initio results from the literature data is included in Supporting Information, Table S4. 

Despite the low rate coefficient predicted by ab initio results, experiments have thoroughly documented the existence of macromonomers [30,64,65,66], which are the product of β-scission; hence, the ab initio model might be misrepresenting the experimentally observed β-scission reaction. Knowing that other quantum chemical approaches predict similar rate coefficients, the discrepancy is therefore likely related to the inadequacy of the chosen pentamer model under actual polymer chain conditions; there must be an extra effect lowering the barrier, which is not included in a typical model structure. The above-mentioned approach of including all enantiomers will impact the β-scission rate coefficients less than backbiting because the spatial arrangement of the alkyl groups is similar in the reactant and transition states. Therefore, we explored other aspects (i.e., the reduced degree of freedom in an actual chain). In a real MCR, the chain segment where the β-scission reaction occurs is embedded in a longer polymer chain at both sides. This backbone reduces the degrees of freedom available for those monomer units involved in the reaction. Hence, the rest of the polymer will impose the strain, compression and limitation of the lateral movements over the segment. Thus, we investigated whether reducing the internal flexibility by constraining the geometry (i.e., mimicking the embedding in the polymer chain) can reduce the activation barrier and bring it closer to the experimental observed value.

If strain is applied to a straight polymer chain, the angles between the main-chain carbon atoms can be expected to become wider, and the covalent bonds between these carbon atoms can be expected to become slightly longer. From a thermodynamic point of view, if the covalent bond length increases, the structure’s energy does as well, easing the bond-breaking reaction. And if this effect impacts the transition state less than the reactant structure, then it results in a lower reaction barrier. On the contrary, by the compression of the polymer chain, the structure bends to reduce steric interactions and produces a twisted, more compact arrangement. This geometry will increase the reaction barrier because the structure needs to stretch first to make the β-scission possible, causing an effect that will not dominate the rate coefficient. Additionally, not only longitudinal but also transversal movements can occur in the chain, and the real effect of the transversal movement on the structure will be like strain or compression, depending on the movement of the main chain. Therefore, the transversal-movement impact is considered by the strain and compression effects. Strain and compression are effects that ease and complicate the β-scission reaction. If the rate coefficient prediction considers both, the rate-enhancing effect will dominate the final rate coefficient. Hence, the strain effect will be included in the model to better represent an actual MCR embedded in a polymer chain.

**Figure 6:**
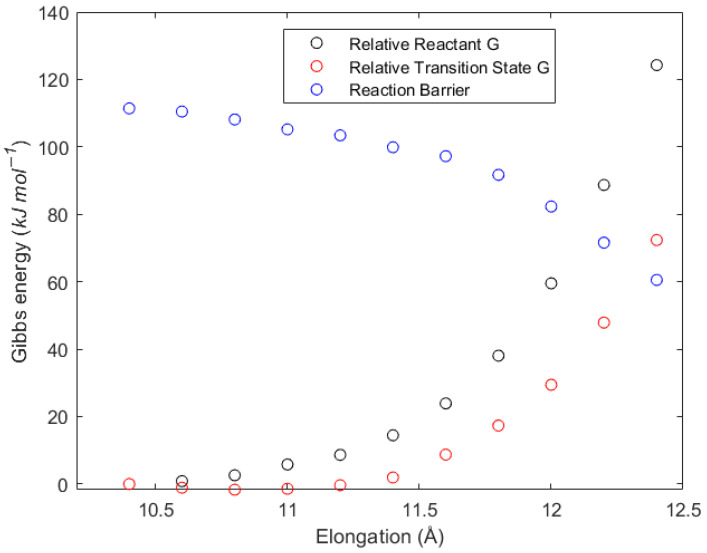
Reduced-flexibility effect on each structure involved in the reaction. The black circle (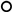) represent the Gibbs energy of a reactant structure with a certain elongation, relative to the non-elongated reactant molecular model. The red circle (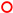) represents the Gibbs energy of the transition-state structure with a certain elongation, relative to the non-elongated transition-state molecular model. The blue circle (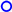) represents the Gibbs reaction barrier at a certain elongation.

To include strain in the model and mimic the interactions with the polymer backbone, we limit the flexibility of the polymer chain by imposing a maximum-elongation restriction on both the reactant- and transition-state structures. For this, we fix the distance between the first and last carbon atoms in the main chain of the pentamer model and increase the distance gradually. This geometry restriction increases the energy in both the reactant and transition states, as Figure 6 shows. The electronic energies of both structures become larger as the distance grows, yet the increase is much larger for the reactant structure than for the transition state. The rigid nature of the MCR structure causes the higher increase in energy, while, in the transition state, the increased flexibility of the breaking bond makes the energy less susceptible to elongation. Therefore, the effect of the flexibility reduction decreases the reaction barrier for the β-scission, as shown in Figure 6. Thus, we confirmed that the imposed strain lowers the barrier for the β-scission reaction and contributes to the better agreement of the calculated and experimental values of the rate coefficient.

The actual strain present in a polymer chain will be a strongly time-dependent phenomenon, as the polymer chain mobility, chain length, temperature, dilution, stirring rates, etc., will affect it. If the reactant is stretched to the extreme, at some point, one main-chain bond will break, and the structure will resemble the products. Because no strict criterion exists for the optimal extension distance, the elongation that replicates an activation energy like the experimental reported values is chosen. Table 4 displays the rate coefficients and activation energies depending on the distance restriction. At 12.0 Å, an elongation of 20.8% of the reactant and 15.4% of the transition state is reached, and the activation energy decreases to 86.9 kJ mol^−1^. The rate coefficient at said elongation is 86.3 s^−1^ at 383.15 K, very similar to the high-accuracy experimental values of Vir et al. [50] (71.5 ± 9.7 s^−1^), reported in Table S4, indicating that the constrained geometry is a realistic representation of an actual polymer mid-chain radical. The rate coefficient based on an elongation of 12.0 Å was used in the *k*MC simulations (see Section 3.8).

Introducing strain to predict the rate coefficient of the β-scission is valid for radicals that are in the middle of the backbone, such as the products of radical migration or CTP. Those MCRs produced directly from ECR backbiting (bbMCR) have more freedom of movement on one side of the backbone, compared to the model we are discussing in this section. Hence, this bbMCR undergoes β-scission at a slower rate, similar to Equation (5). Despite this difference, the overall rate coefficient for the β-scission will be dominated by the middle-of-the-backbone MCR because its rate coefficient is several orders of magnitude higher than its bbMCR counterpart, even if the fraction is less than 0.1% of the total MCRs. Because the restricted-mobility approach gives good results for the acrylates, it can improve the accuracy of ab initio rate coefficient prediction for other monomers as well.

### 3.3. Migration

**Figure 7:**
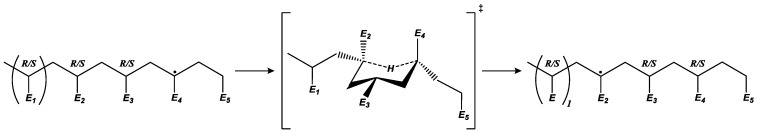
General methyl acrylate pentamer structure with the mid-chain radical on position 4, as used for the migration reaction model.

During the migration reaction, the MCR changes its position on the main chain through a hydrogen transfer mechanism, forming a six-membered ring structure similar to that formed via backbiting. A pentamer model was chosen to maintain consistency with the other secondary reactions. The reactant structure possesses the radical in the fourth unit, as Figure 7 shows, instead of in the fifth unit, as performed to study the backbiting (Figure 2). Afterwards, this radical transfers towards the second unit during the migration reaction.

Similar to backbiting, for the migration reaction, the presence of multiple optical isomers can have a significant influence on the values of the predicted rate coefficients. Therefore, we applied the same weighted-average approach to evaluate the value of the $k_{MIG}$. For this case, only four optical isomers can be defined because the MCR eliminates one chiral center (Figure 7), making this model simpler than the eight permutations used for backbiting. Table 5 shows the rate coefficients, the weighting factors derived from the propagation probabilities and the final weighted averages for these optical isomers.

Table 5 shows that, as in the backbiting prediction, the rate coefficients for the migration range over different orders of magnitude depending on the chirality. According to these results, the rate coefficient decreases opposite to the number of alternating chiralities in the optical isomer. This means that the largest migration rate coefficient is observed for the isotactic sequence (RRR/SSS), which is the most unstable optical isomer and the most stable transition state. Conversely, the slowest rate coefficient is predicted for the syndiotactic sequence (RSR/SRS), which is the most stable enantiomer. Both structures with one alternating and one identical chirality (SRR/RSS and RRS/SSR) are within these two extremes, although the SRR/RSS enantiomer rate coefficient is similar to that of the syndiotactic sequence (RSR/SRS) (Table S3 in Supporting Information shows a thorough comparison of the energies). Eventually, we calculated the value of the rate coefficient as the weighted average of the rate coefficients for the individual isomers, in which the weighting factors are given by the abundances of the enantiomers presented in Table 1. (Table 5; $k_{MIG}=$ 2.92 × 10^5^ s^−1^ at 413 K). Interestingly, the isotactic sequence (RRR/SSS) dominates the weighted-average rate coefficients and Arrhenius parameters despite being the less probable isomer, showing the importance of considering all possible enantiomers within a reaction.

In contrast to backbiting, the rate coefficient predicted for migration using the weighted-average approach does not agree with the literature data. At the temperature of 413 K, Van Steenberge et al. [54] reported 1.6 × 10^2^ s^−1^, obtained from fitting the experimental data via kinetic Monte Carlo simulations, while Ballard et al. [67] reports 3 × 10^3^ s^−1^, obtained through experimental measurements. Both these reported rate coefficients disagree with the value predicted in this work, as they are from two to three orders of magnitude lower. The predicted rate coefficient for migration is even faster than the calculated rate coefficient for backbiting at said temperature. This is not what one would expect thermodynamically: the radical transfer from a tertiary-to-tertiary carbon atom, such as in migration, is expected to be slower than a radical transfer from the secondary to tertiary carbon atoms, as in backbiting. This inconsistency is addressed in the next paragraph.

**Figure 8:**
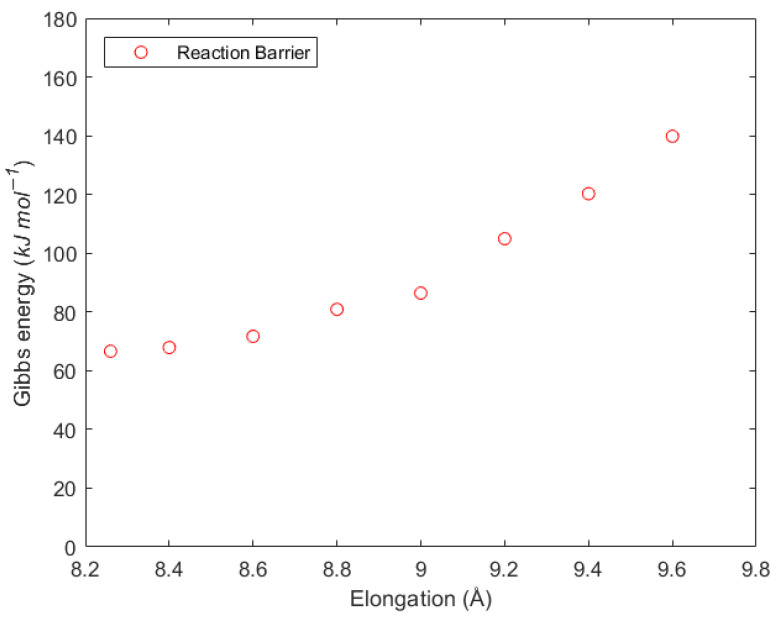
Δ^‡^G (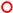) vs. elongation of the radical migration reaction barrier applying the reduced-flexibility approach.

In the calculations above, we allowed the free motion of the model polymer chain. However, similar to β-scission, the migration reaction proceeds in an MCR embedded in a polymer backbone on both sides. The rest of the chain will limit the flexibility of the model segment, making it an interesting case to test the procedure previously used for the β-scission. The main chain must contract to form the six-membered ring structure representing the transition state. In this process, the rest of the backbone will restrict the movement in the real polymer, while this effect lacks representation in the computational model. Figure 8 shows the effect of the reduced flexibility in the G^‡^ for the migration reaction. In this case, the Gibbs energy barrier increases as the flexibility is reduced, in contrast to the β-scission behavior, where the barrier decreases as the flexibility is reduced. Therefore, the reduced-flexibility approach correctly predicts the impact of the strain in the transition state, demonstrating that the influence of the backbone can explain the difference between the experimental and predicted rate coefficients. Similar to the β-scission reaction, the Gibbs energy graph lacks an objective criterion for selecting an elongation that can reproduce the correct barrier. For the migration reaction, an approach based on thermodynamic reasoning is proposed: the rate coefficient of the migration reaction should be in the same order of magnitude as that of the backbiting, as they are similar reactions, but the strain imposed by the backbone should make the rate coefficient reasonably smaller than that obtained via backbiting.

Finally, the elongation value chosen corresponds to 8.80 Å. This provides a G^‡^ value of 82.2 kJ mol^−1^ and a rate coefficient of 1.60 × 10^3^ s^−1^ at 413.15 K, as seen in Table 6. An elongation of 6.5% provides a reasonable value for the rate coefficient instead of the 11.56% required for β-scission. This difference relates to the fact that the six-membered-ring transition state (Figure 6) for migration is rigid and not affected by the elongation. Therefore, the effects of elongation are distributed over less C-C bonds in comparison to β-scission, explaining the smaller elongation required.

### 3.4. Macromonomer Propagation

Macromonomers are the products of a β-scission reaction and are therefore chains that contain unsaturated ends. Their reactivities are similar but not identical to acrylate monomers, as they can undergo a propagation reaction that produces a polymer chain with an MCR and a side branch. 

**Figure 9:**
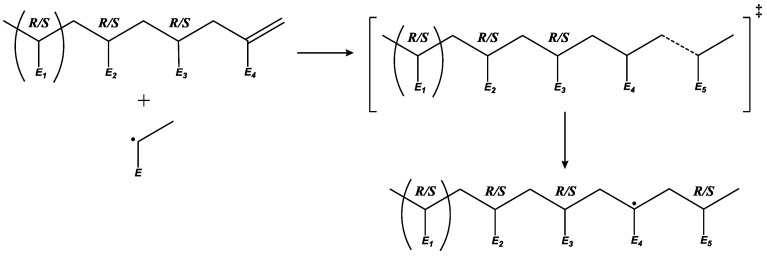
Macromonomer propagation model: unimer radical propagating to a tetramer macromonomer.

Figure 9 displays the molecular model, which consists of a unimer radical added to an unsaturated methyl acrylate tetramer. Because the backbone spatial disposition remains through the reaction, the influence of the chiralities of the nearby units on the reaction can be expected to be negligible. Consequently, no further considerations were made.

Table 7 shows the predicted rate coefficient at 413.15 K and the pre-exponential factor and activation energy for macromonomer propagation. This table includes, for comparison, the rate coefficient used by Van Steenberge et al. [54] in their kinetic Monte Carlo simulation based on the experimental electron spray ionization mass spectra measured by Junkers and Barner-Kowollik [25] for macromonomer synthesis. Additionally, we included the rate coefficient determined by Wang et al. [30] The comparison in Table 7 clearly shows that we predicted a rate coefficient 20% higher than those of other authors, which can be considered a good agreement. Moreover, it is within the same order of magnitude as the predicted ECR propagation rate coefficient: 2.18 × 10^5^ vs. 3.02 × 10^5^ L mol^−1^ s^−1^ for the ECR and macromonomer propagation, respectively. From a thermodynamic point of view, the macromonomer propagation is slightly faster because, in this reaction, a more stable MCR, (i.e., a tertiary carbon radical) is formed instead of the secondary radical formed during regular propagation. The associated more-negative-reaction enthalpy typically results in a decrease in the activation energy, following the Brønsted−Evans−Polanyi relation, as was also observed in this work.

### 3.5. MCR Propagation

**Figure 10:**
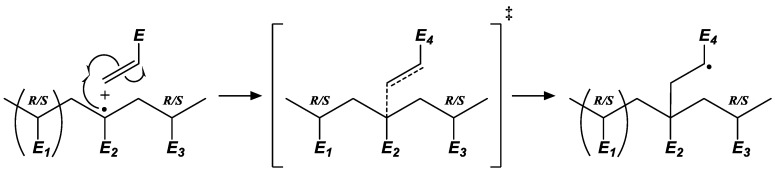
Mid-chain radical propagation model. A trimer structure with an MCR in the middle unit propagating to an MA monomer.

The selected molecular model for the MCR propagation is a trimer structure with a radical in the central unit that can be added to a monomer, as shown in Figure 10. To account for the influence of optical isomerism, we considered two possibilities for the adjacent monomer units: one with the same chirality (R-MCR-R) and another with different chiralities (R-MCR-S). The results are shown in Table 8 along with the literature data, which include the rate coefficients from multiple acrylates.

The experimental results agree quite well with those of different authors in the literature [27,50,58,60,68,69,70], independently of the method used to measure the rate coefficient. In this work, the predicted rate coefficient is in decent agreement with the experimental values because it is less than a factor of two larger than the mid-chain radical propagation for the methyl and n-butyl acrylate experimental values. Thus, our method accurately predicts the rate coefficient of MCR propagation.

**Table 8:** MCR propagation predicted rates and published results.

Acrylate	*k* @ 298.15K [L mol^−1^ s^−1^]	*k* @ 413.15K [L mol^−1^ s^−1^]	*A* [L mol^−1^ s^−1^]	*E_a_* [kJ mol^−1^]	Source
methyl R-MCR-R	3.11 × 10^1^	6.78 × 10^2^	6.5 × 10^6^	30.0	This work
R-MCR-S	3.66 × 10^1^	1.06 × 10^3^	2.0 × 10^6^	27.5	
WA	3.36 × 10^1^	8.49 × 10^2^	3.7 × 10^6^	28.8	
methyl	1.79 × 10^1^	3.64 × 10^2^	8.9 ± 0.5 × 10^5^	26.8 ± 1.5	[60]
*n*-butyl	1.31 × 10^1^	3.37 × 10^2^	1.52 ± 0.14 × 10^6^	28.9 ± 3.2	[58]
*n*-butyl	1.05 × 10^1^	2.51 × 10^2^	9.2 × 10^5^	28.3	[71]
*n*-butyl	1.05 × 10^1^	3.10 × 10^2^	1.98 × 10^6^ *	30.1 ± 9.7	[50]
*t*-butyl	2.87 × 10^0^	3.21 × 10^1^	1.68 × 10^4^ **	21.5 ± 3.6	[69]
dodecyl	4.57 × 10^0^	1.12 × 10^2^	4.5 ± 0.8 × 10^5^	28.5 ± 1.4	[60]

* Recalculated from the original report stating ln(A) = 14.5 ± 3.7. ** Recalculated from the original report stating ln(A) = 9.73 ± 0.70.

### 3.6. Chain Transfer to Monomer

**Figure 11:**
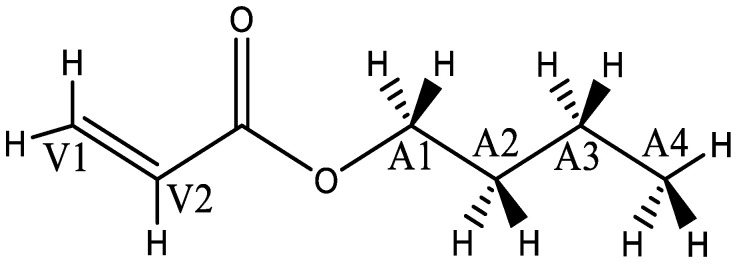
Carbons atoms possessing abstractable hydrogens in the CTM model of a butyl acrylate monomer molecule.

Chain transfer to monomer (CTM) is a reaction in which a hydrogen is abstracted by an ECR from an acrylate monomer, leading to the termination of the growing radical polymer chain and the formation of a new unimer radical, as the monomer molecule contains several hydrogen atoms that can be abstracted (Figure 11). For carbon atoms possessing abstractable hydrogens in the CTM model of a butyl acrylate monomer molecule, the chain transfer to monomer can yield different types of products. In most of the experimental studies, it is not specified which H atom is being abstracted, as this is difficult to determine experimentally. Therefore, the rate coefficient is an overall rate coefficient considering all the abstraction reaction possibilities. To simulate this with ab initio methods, all reaction possibilities between these two species should be taken into consideration, and the $k_{CTM}$ should be calculated as a sum of all the contributions. 

The *n*-butyl acrylate monomer possesses twelve hydrogen atoms in total that are available for abstraction (Figure 11): within the vinylic group, carbon atom V1 possesses two H atoms and V2 has one H atom; within the alkyl substituent, the secondary carbon atoms A1–A3 possess two H atoms each, and the primary carbon atom A4 has three hydrogen atoms. It should be noted that the molecular model for all previous reactions is represented with methyl acrylate for computational efficiency. However, for the chain-transfer-to-monomer reaction, we need to use a butyl substituent in our model to consider all the H atoms of the side chain in the chain-transfer-to-monomer reaction. The structure and side chain of the model radical representing the abstracting radical are less relevant, so a dimer methyl acrylate end-chain radical suffices. Table 9 shows the rate coefficients and Arrhenius parameters predicted by this work along with those in literature for H abstracting. As expected, the abstraction of hydrogens within the vinylic group (V1 and V2) has a high barrier because of the unstable vinylic radical products; hence, disregarding these reactions even at high temperatures induces little error. The results for abstractions from the *n*-butyl acrylate in atoms A1–A3 are similar, which is expected because of the resemblance between the reactions. The chain transfer to monomer of the primary carbon A4 has a higher activation energy than those of the secondary carbon abstractions (A1–A3), but the abstraction remains faster than those from V1 and V2. This reaction in atom A4 proceeds faster than those in the vinylic group. Consequently, the computational results show that the abstraction of hydrogen preferably occurs from carbon atoms A1 to A3.

In Table 9, we also compare the literature values for the chain-transfer-to-monomer-reaction rate coefficients. The main difference between ab initio and experimentally derived data is that the former predict elementary reactions and the latter measure the apparent rate coefficients. To carefully consider each possible abstraction, each rate coefficient must be multiplied by the number of hydrogen atoms via Equation (5), except for the last three hydrogen atoms in the alkyl chain, as these will already be included in the threefold symmetry for the methyl rotation. The results are shown in Table 9.

(6) $$k_{CTM}=k_{V1}+k_{V2}+2\times \left(k_{A1}+k_{A2}+k_{A3}\right)+k_{A4}$$

Other authors have performed ab initio predictions in the gas phase in chain transfer to monomer [36,38]. Their results display lower rate coefficients than our predictions in this work, probably due to the differences in the chosen computational levels, but the trend between the abstracted hydrogen atoms is maintained independently of the method/basis set or model chosen. Most of the experimental reported data show smaller rate coefficients in comparison to our predictions, while the most recent research shows similar rate coefficients. A table with a thorough comparison is shown in Supporting Information, Table S4.

### 3.7. Chain Transfer to Polymer

**Figure 12:**
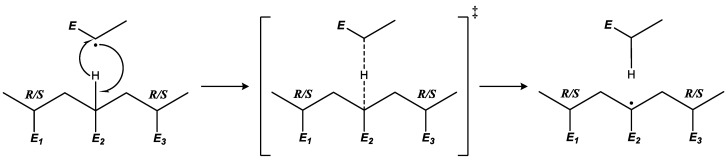
Chain-transfer-to-polymer model. An ECR monomer abstracting a hydrogen from the methyl acrylate trimer in the middle unit.

Chain transfer to polymer comprises an ECR abstracting a hydrogen atom from a random point in the middle of the polymer chain, producing an MCR and a dead-polymer chain. Figure 12 shows this reaction for the molecular model that we used for the calculations: a unimer ECR abstracting a hydrogen atom from a trimer methyl acrylate chain in the middle unit. Additionally, we predicted the kinetic parameters of the abstraction of hydrogens atoms from the alkyl chain (butyl acrylate), based on the rate coefficients predicted for the chain transfer to monomer. Hence, we calculated the chain-transfer-to-polymer rate coefficient ($k_{CTP}$) as the sum of the chain transfer to backbone ($k_{CTB}$), depicted in Figure 12, and the chain transfer to alkyl branch ($k_{CTA}$).

During chain transfer to polymer, a large polymer chain approaches the middle of another large chain. As the abstracted hydrogen bonds to a tertiary carbon in the middle of the chain, the steric interaction of nearby units impacts the space available for the reaction. Therefore, for this reaction, we considered all possible chiralities and weighted their average for the trimer molecular model. Four different chirality permutations exist for the trimer: RRR/SSS, RSR/SRS, RRS/SSR and SRR/RSS. Permutations RRS/SSR and SRR/RSS are symmetrically equivalent enantiomers, as Figure 12 shows (chain-transfer-to-polymer model). An ECR monomer abstracting a hydrogen from the methyl acrylate trimer in the middle unit shows because of the extra methyl group addition on unit 3. As a result, the rate coefficients of both permutations are equal in the weighted average. Additionally, Table 10 shows the results along with the weighted average of the chain transfer to backbone (${\bar{k}}_{CTB}$), which was calculated by using Equation (3), and the weighting factors ($\omega_{i}$) derived similarly as in the previous sections.

Chain transfer to polymer is typically measured in terms of the side branches via ^13^C NMR or SEC trace fitting. Any reaction that contributes to the number of side branches might add its effect to the apparent rate coefficients. Therefore, it is interesting to study all hydrogen atom abstractions from an embedded monomer unit and not only the abstraction from the tertiary carbon atom embedded in the main chain. For *n*-butyl acrylate, abstractions of hydrogen atoms from the ester substituent are possible. The rate coefficient for these abstractions can be extrapolated from chain transfer to monomer because their magnitude resembles the chain-transfer-to-polymer rate coefficient. Table 10 presents the rate coefficients of chain transfer to the alkyl side chain of the monomer ($k_{CTA}$).

This new secondary radical in the alkyl chain could propagate with a similar rate coefficient as ECR propagation and, after further propagation steps, resemble a side branch in the backbone. In their work, Boschmann and Vana [29] show an increase in the chain-transfer-to-polymer rate coefficient based on the number of carbons, from 0.3 to 7.1 L mol^−1^ s^−1^ between butyl and dodecyl acrylate. A possible reaction for this radical is intramolecular transfer to the main chain through a similar ring structure as in backbiting or migration, though it will be a seven-, eight- or nine-atom structure depending on the radical location. Preliminary predictions on the eight-ring structure yield a rate coefficient like chain transfer to monomer, so this radical is more likely to propagate, and its rate is excluded from this data set. In conclusion, the chain-transfer-to-polymer rate coefficient ($k_{CTP}$) is the sum of the transfer to backbone (${\bar{k}}_{CTB}$) and transfer to alkyl side chain ($k_{CTA}$).

Many groups have studied chain transfer to polymer in the last two decades but have found no general agreement in terms of the rate coefficient values. Table S5 displays a clear summary of previous works [26,29,36,54,57,73,74]. The experimentally measured rate coefficients at a single temperature could differ by three orders of magnitude, and the differences in the activation energies are as high as 20 kJ mol^−1^.

A comparison of this work’s predicted rate coefficient for chain transfer to polymer yields a general agreement, in terms of order of magnitude, with the low-side rate coefficients of Plessis et al. [73] and Arzamendi [57]. However, the activation energies differ by over 10 kJ mol^−1^, and the pre-exponential factors differ by two orders of magnitude. Nonetheless, an exact match exists between this work’s rate coefficient and that of Boshmann and Vana’s work [29] via Z-RAFT polymerization and C NMR analysis. When comparing it to the recent rate coefficient values of Ballard et al. [74] and Van Steenberge et al. [54], which are on the high end, this work’s value is lower by an order of magnitude at 413.15 K. To the best of our knowledge, this manuscript is the first to report an ab initio chain-transfer-to-polymer rate coefficient using a proper model structure product of head-to-tail propagation.

Summarizing the different methodologies reported in the literature, various authors report rate coefficients that differ by multiple orders of magnitude. No clear conclusion exists on which rate coefficient is more likely than the other reported values. In conclusion, the chain-transfer-to-polymer reaction is difficult to measure and model because diverse reactions can create side branches, and the consideration of all of them is required to predict the correct overall rate coefficient. We will benchmark whether this approach provides a reasonable prediction in the simulations shown in the following section, where the chain-transfer-to-polymer rate coefficient is applied in the simulation of ESI-MS spectra.

### 3.8. kMC Simulations

To evaluate the effect of our calculated rate coefficients on the polymerization kinetics, the *k*MC model for acrylate polymerization from Vir et al. [50] was taken. This model works as a stochastic tool to solve the kinetic system that simulates the PLP-SEC experiment as an alternative to solving the set of kinetic differential equations. Then, the results from the simulations using the rate coefficients reported above were benchmarked against the experimental and simulated data of PLP-SEC reported by Vir et al. [49,50] and Marien et al. [75], as well as ESI-MS for the synthesis of macromonomers obtained via the activation of bromine-capped p-(*n*-butyl acrylate). In order to decouple the effect of each reaction as much as possible, we used a gradual approach for the selection of the experimental conditions to be simulated, with the objective of maximizing the sensitivity towards a single particular secondary-reaction rate coefficient. The values of the propagation, backbiting and β-scission were benchmarked against PLP-SEC data, whereas the values of the migration and chain-transfer-to-polymer rate coefficients, the effects of which are difficult to derive from the SEC trace, were benchmarked against ESI-MS.

Table 11 reports the data sets used for the simulation of the PLP-SEC experiments. We selected three sufficiently different experimental conditions: (1) 306 K, in bulk, at a laser frequency of 500 Hz; (2) 306 K, with a solvent fraction of 0.75 and at a laser frequency of 50 Hz; (3) 413 K, in bulk, at a laser frequency of 10 Hz. The first data set uses the reference parameters reported by Vir et al. [50] The second data set uses this work’s predicted rate coefficients without including special considerations: the backbiting Arrhenius parameters are chosen with a single enantiomer (RSSS/SRRR) in a narrow low-temperature range and the β-scission excludes the reduced-flexibility approach. In the third data set, the backbiting rate coefficient is replaced by the weighted average of all the enantiomers in a narrow temperature range. Finally, in the fourth data set, the unconstrained β-scission rate coefficient replaces the reduced-flexibility one. Hence, the expectation is that Data Set 4 should approximate Data Set 1 the best, while Data Sets 2 and 3 should only provide moderate or qualitative agreement with the experiment.

**Figure 13:**
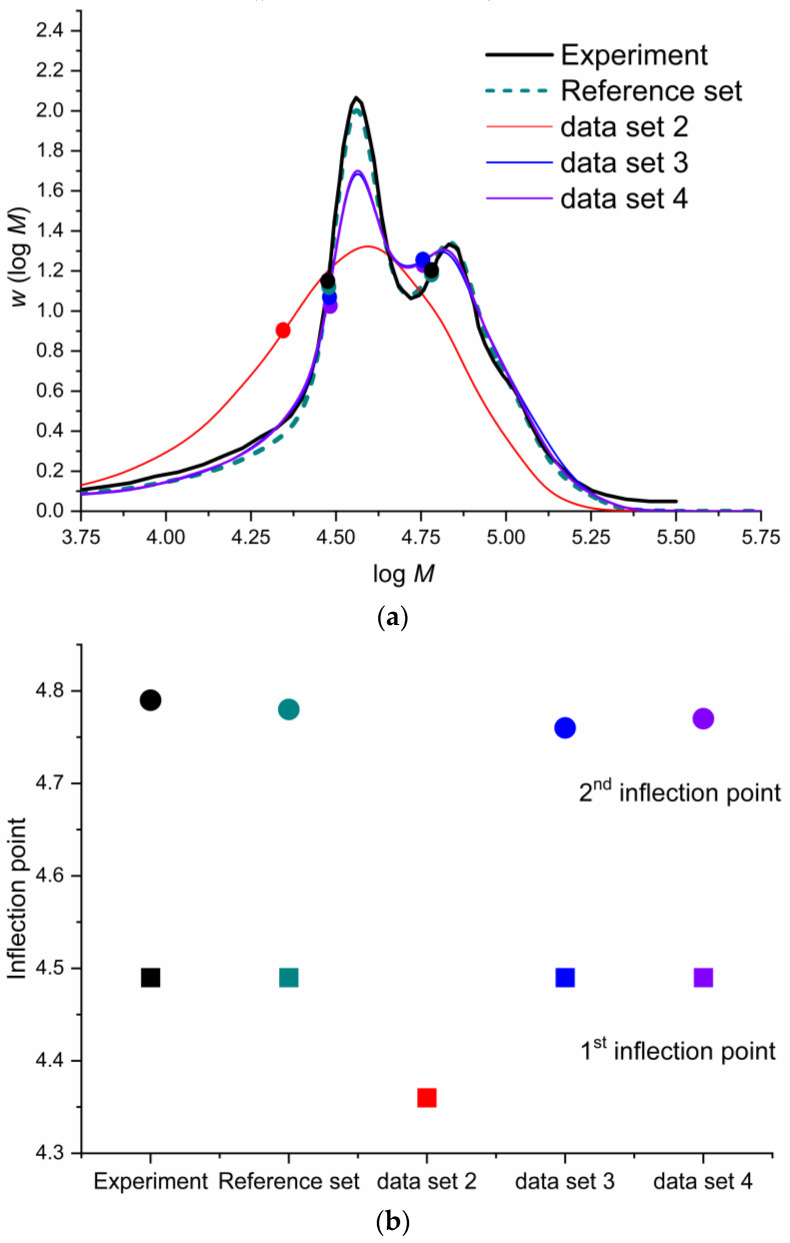
Experiment (1): PLP-SEC trace experiment of n-butyl acrylate at 306 K, in bulk, at laser frequency of 500 Hz. Comparison between experiment [49], *k*MC-simulated SEC trace with parameters derived from [50] and this work’s predicted rate coefficients via different methods.

The first experiment (Figure 13) is sensitive to the value of the ECR propagation because the laser frequency is high, and it lowers the impact of all the secondary (slow) reactions. The fitted parameters published by Vir et al. [50] reproduce a perfect fit of the experimental SEC trace displayed in Figure 13a, including a perfect match for the first and second inflection points (the points in Figure 13a,b). Data Set 2, without special considerations, incorrectly predicts the SEC trace, as depicted in Figure 13a. The simulated trace (red line) loses the bimodality of the experimental trace because the backbiting reaction frequency (*k*_bb_) is higher than the laser frequency. This allows backbiting to proceed even under these conditions, limiting the further propagation of radicals surviving the next pulse that produces the bimodality. This effect limits the chain size and impedes the second-peak formation in the SEC trace. Data Set 3 includes the rate coefficients predicted by the weighted average for backbiting, which massively improves the SEC trace (blue) simulation because the backbiting rate coefficient is now smaller than the pulsed-laser frequency. This data set predicts the first inflection point accurately, and the second inflection point is slightly lower than the experimental value. Data Set 4 includes reduced flexibility for β-scission reactions, and although it has no apparent effect on the SEC trace (purple), it slightly improves the prediction of the second-inflection-point prediction. In conclusion, including these methods shows improvement in the simulated SEC trace because they predict the experimental bimodality. Although they do not produce a perfect match, the inflection points shown in Figure 13b have minimal errors in their predictions.

**Figure 14:**
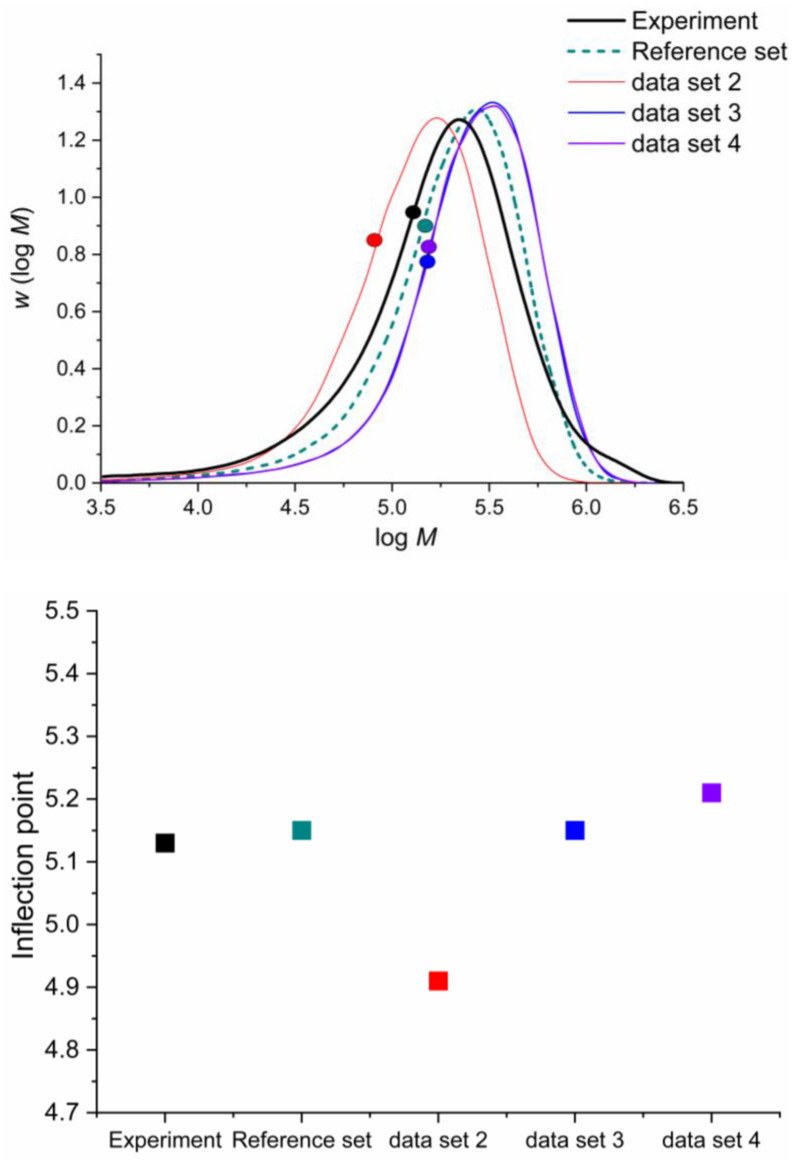
Experiment (2): PLP-SEC trace experiment of n-butyl acrylate at 306 K, a solvent fraction of 0.75 and a laser frequency of 50 Hz. Comparison between experiment [75] and *k*MC-simulated SEC trace with parameters derived from Vir et al. [50] (reference set) and this work’s predicted rate coefficients via different methods (data 2, 3 and 4).

Experiment (2) provides the ideal conditions for enhanced the backbiting sensitivity to the SEC trace because it features a low pulse frequency and low monomer concentration. Figure 14 presents the simulation results. Experiment (2) involved a PLP-SEC trace experiment of n-butyl acrylate at 306 K, a solvent fraction of 0.75 and a laser frequency of 50 Hz. A comparison was made between the experiment [75] and *k*MC-simulated SEC trace with the parameters derived from Vir et al. [50] Data Set 2, without special considerations, underestimates the inflection point. Although the SEC trace (red) has the desired shape, it is shifted to the left, pointing towards a lower molecular mass induced by a higher backbiting rate, which slows the apparent propagation rate. Data Set 3 shows that including the consideration of all enantiomers for backbiting greatly improves the inflection point prediction, which is almost in perfect agreement with the experiment, although the SEC trace (blue) is slightly right-shifted. Data Set 4, including the β-scission reduced flexibility, seems to worsen the inflection point prediction, although it is within an acceptable range for ab initio prediction.

**Figure 15:**
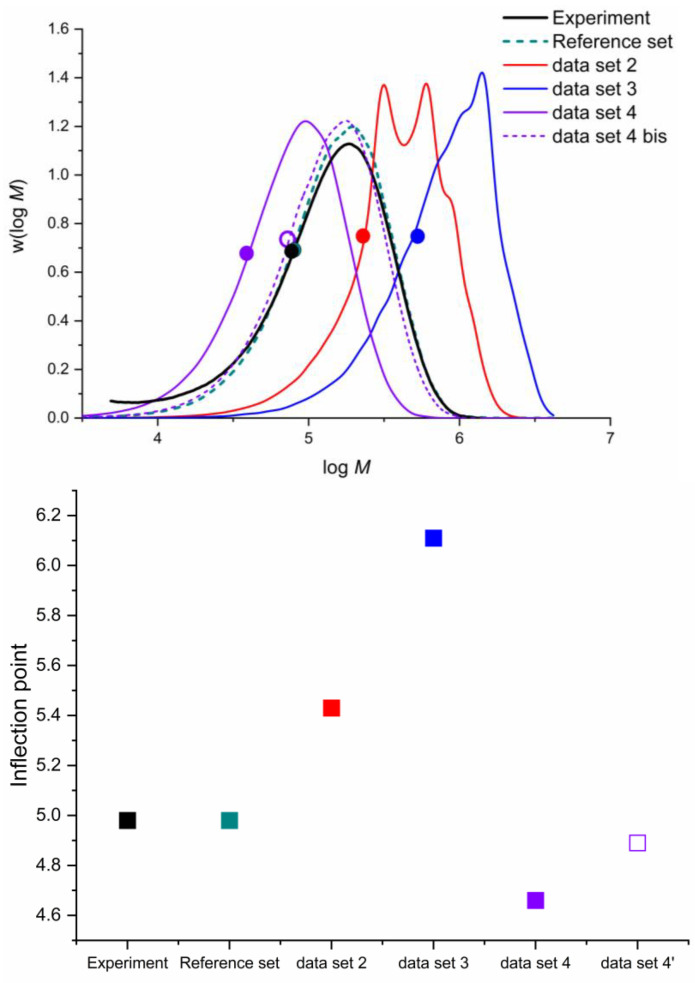
Experiment (3): PLP-SEC trace experiment of n-butyl acrylate at 413 K, in bulk, at laser frequency 10 Hz. Comparison between experiment [50] and *k*MC-simulated SEC trace with parameters derived from Vir et al. [50] (reference set) and this work’s predicted rate coefficients via different methods (data 2, 3 and 4).

Figure 15 shows the simulation results compared to the experiment (3). The experimental design enhances the secondary reaction’s effect by increasing the temperature and lowering the laser frequency. Data Set 2 simulates an SEC trace (red), which is far right-shifted to the experimental one and even possesses a bimodality that is nonexistent in the experimental results. This effect is probably the result of a slower β-scission, which reduces the chain-breaking effect, allowing the chains to grow further, which right-shifts the SEC trace. Data Set 3, which includes the consideration of all enantiomers for backbiting, also predicts a poor agreement between the ab initio and experiment results, shifting even more to the right of the SEC trace (blue) and increasing the inflection point even more. Data Set 4 includes the reduced flexibility for the β-scission, resulting in a decent agreement with the experiment. The approach applied in this data set recovers the unimodality of the SEC trace and lowers the inflection point at least to the same order of magnitude as the experimental result (black line). The SEC trace of Data Set 4 (purple) left-shifts the points towards lower molecular masses. This effect might be induced by the model predicting a higher β-scission rate coefficient, which is because the said reaction causes a chain-breaking effect that left-shifts the SEC trace. Further adjustment of the β-scission rate coefficient by multiplying it with a factor of 0.5, which is within the typical confidence interval for ab initio predicted rate coefficients, produces Data Set 4′ shown in Figure 15 (purple dotted line for the SEC trace and purple void square for the inflection point). This improves the prediction greatly, as now both the SEC trace and the inflection point are in great agreement with the experiment, implying that the ab initio prediction is relatively good but slightly overestimates the β-scission rate coefficient. This overestimation of the rate coefficient could be related to the previously discussed difference between middle-of-the-backbone MCRs and recently backbit MCRs, where the former reacts much faster than the latter. Multiplying the β-scission rate coefficient with a factor of 0.5 is, in this interpretation, equivalent to assuming that 50% of the MCRs are “recently backbit MCRs” that decompose much slower. The large impact of this small change in the β-scission rate coefficient on the prediction of the SEC trace and inflection point prediction highlights the strong sensitivity of these reaction conditions to the β-scission rate coefficient.

**Figure 16:**
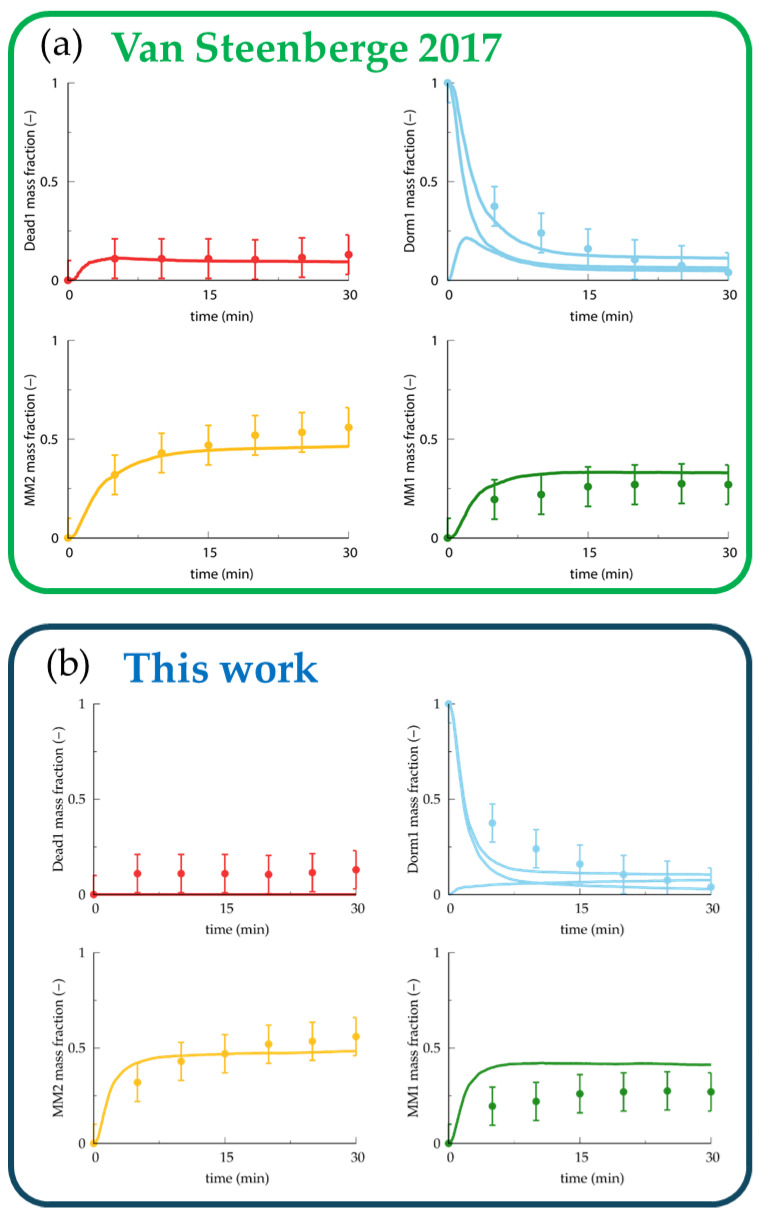
Simulated (full lines) and experimental (points) mass fractions corresponding to the main ESI-MS signals of the synthesis of macromonomers (MMs) via the activation of bromine-capped poly(*n*-butyl acrylate) in Van Steenberge et al. [54], top left, red; dead-polymer product of CTP, top right, blue; dormant Br-capped p-(n-BA) including the fraction of activated ECRs and MCRs, bottom left, yellow; MM product of right β-scission, bottom right, green; MM product of left β-scission.

The last set of simulated experiments (i.e., the ESI-MS data for the synthesis of macromonomers (MMs) via the activation of bromine-capped poly(*n*-butyl acrylate)) is sensitive to the values of the macromonomer propagation, chain-transfer-to-polymer and migration rate coefficients. We compared the data sets for these rate coefficients used by Van Steenberge et al. [54] and report them here in Table 12. Figure 16 shows the ESI-MS simulation. In general, the simulations with our data set agree with the experimental behavior, except for the “dead”-polymer-fraction prediction.

As seen in Figure 16b, the “dead”-polymer red curve does not describe the experimental data points. The calculated rate coefficient with the largest uncertainty is the chain-transfer-to-polymer reaction because of the large spread within the experimental data and between the experimental and predicted data. The red curve is not sensitive to the chain-transfer-to-polymer rate coefficient, as an increase of 1000 times in the predicted rate coefficient barely modifies the red curve (see Supporting Information, Figure S4). Moreover, the light-blue curve shows a steeper decay in the ECR species with the parameters presented in this work. The difference relates to the faster backbiting and migration reactions, tilting the initiation equilibria towards more MCRs compared to ECRs at late reaction times. These MCRs in the present data set would rather undergo β-scission than chain transfer to polymer. This increases the amount of macromonomers, as depicted in Figure 16b, bottom right, and might be a key factor in the number of “dead” polymers in the mixture.

It is important to mention that the reliability of the ab initio prediction is enhanced by testing the results against the experimental data under very different conditions with sensitivities towards different types of reactions. This task could not be possible without the integration of the ab initio predictions into the *k*MC simulations, showing that the combination of both techniques could provide further insight into the polymerization kinetics. The results are far from a perfect fit but provide decent agreement.

## 4. Conclusions

In this article, we report a complete and consistent calculated set of rate coefficients predicted through ab initio methods for acrylate radical polymerization. To enhance the accuracy of the prediction, we apply two new approaches: (1) calculation of the weighted average for the reactions influenced by the optical isomerism of the molecular model, and (2) geometrical constrains to mimic the restricted motion of the segments embedded in the middle of the polymer chain. Instead of comparing the rate coefficients one on one to the literature values, the obtained rate coefficients were benchmarked against experimental data via *k*MC simulations. The actual experiments and applied experimental conditions varied widely in order to selectively address the isolation of the secondary reactions as much as possible, a methodology that proves suitable for comparing ab initio rate coefficients.

We showed that this work’s data set simulates the MMDs of acrylate polymers with an acceptable error, although the predicted rate coefficients differ from those measured via experimental work or with model assistance. Additionally, our two new approaches provide a substantial improvement in the prediction of the experimental SEC traces and their inflection points. 

In any secondary reaction involving significant changes in the chain’s shape during the transition state, considering all enantiomers is crucial. The ab initio predictions of the rate coefficients for the backbiting and migration results show that the reactivities are dependent on the molecular-model chirality. Optical isomers with alternating chiralities have lower rate coefficients for secondary reactions thanks to the increased reactant stability, while, in general, sequential identical chiral permutations possess higher values. This strategy should prove useful for any type of atactic polymer, while, for polymers presenting tacticity, it should suffice to use a model describing the tacticity.

The reduced-flexibility approach shows an improvement in the rate coefficient prediction of the β-scission reaction. Without this approach, the predictions of the SEC trace and inflection points are far off the experimental results. The reduced-flexibility-approach applicability is easily extendable to any other type of macromolecular chemistry.

This work serves as an improvement in the techniques used to predict rate coefficients for the polymerization process that can aid in polymerization modeling. Further development is available for these methods because considering all the optical isomers requires testing against the experimental data of various atactic polymers. The reduction in flexibility requires a proper criterion for the geometry restriction, which could be studied by applying the method to different polymers and comparing the results to high-quality experimental measurements.

## Figures and Tables

**Table 1:** Addition chirality probabilities for ECR propagation in methyl acrylate polymerization.

Addition Probabilities	Experimental Work [55]	This Work
Identical chirality $\left(P_{IC}\right)$	0.52	0.34
Alternating chirality $\left(P_{AC}\right)$	0.48	0.66

**Table 2:** Statistical occurrences of chiral sequences in the applied methyl acrylate pentamer model, based on the addition ratios from Table 1. Experimental values are taken from Satoh et al. [55].

Enantiomer	Experiment (%)	This Work (%)
SSSS/RRRR	14.1	3.9
SSSR/RRRS	13.0	7.6
SSRS/RRSR	12.0	14.8
SRSS/RSRR	12.0	14.8
RSSS/SRRR	13.0	7.6
SSRR/RRSS	13.0	7.6
SRSR/RSRS	11.1	28.7
RSSR/SRRS	12.0	14.8

**Table 3:** Weighted-average approach applied to methyl acrylate backbiting using Equation (4).

Enantiomer	$\mathrm{Simulated}\;\omega_{i}$	*k* [s^−1^] 298.15K	*k* [s^−1^] 413.15K	*A* [s^−1^]	*E_a_* [kJ/mol]
SSSS/RRRR	0.039	2.40 × 10^2^	3.96 × 10^4^	2.29 × 10^10^	45.8
SSSR/RRRS	0.076	5.64 × 10^2^	7.87 × 10^4^	2.96 × 10^10^	44.3
SSRS/RRSR	0.148	2.35 × 10^2^	2.16 × 10^4^	2.75 × 10^9^	40.5
SRSS/RSRR	0.148	3.16 × 10^1^	1.04 × 10^3^	1.23 × 10^7^	33.8
RSSS/SRRR	0.076	1.08 × 10^3^	2.94 × 10^5^	5.63 × 10^11^	49.4
SSRR/RRSS	0.076	3.38 × 10^2^	5.90 × 10^4^	3.92 × 10^10^	46.2
SRSR/RSRS	0.287	4.92 × 10^1^	1.55 × 10^4^	4.88 × 10^10^	51.6
RSSR/SRRS	0.148	2.89 × 10^2^	4.82 × 10^4^	2.85 × 10^10^	45.8
${\bar{k}}_{BB}$		2.57 × 10^2^	4.95 × 10^4^	4.09 × 10^10^ *	46.8 *

* If the weighted-average backbiting (${\bar{k}}_{BB})$ is calculated by experimental weighting factors instead of ab initio results, the changes are negligible: the pre-exponential factor changes to 6.34 × 10^10^ and the activation energy changes to 46.9 kJ mol^−1^.

**Table 4:** Activation energies and rate coefficients for the β-scission reaction by restricting the distance between the first and last carbon atoms in the model structures.

Elongation between First and Last Carbon Atoms	*E*_a_ [kJ mol^−1^ s^−1^]	*k* @ 383.15 K [s^−1^]
Non-strained	116.0	5.14 × 10^−3^
10.6	115.1	1.38 × 10^−2^
10.8	112.6	2.89 × 10^−2^
11.0	109.4	7.26 × 10^−2^
11.2	108.0	1.26 × 10^−1^
11.4	104.5	3.84 × 10^−1^
11.6	101.2	8.83 × 10^−1^
11.8	95.8	5.01 × 10^0^
12.0	86.3	9.85 × 10^1^
12.2	75.6	2.77 × 10^3^
12.4	64.5	8.92 × 10^4^

**Table 5:** Migration rate coefficients in high-temperature range (top) and comparison with literature values (bottom).

Enantiomer	$\mathrm{Simulated}\;\omega_{i}$	*k* [s^−1^] 298.15K	*k* [s^−1^] 413.15K	*A* [s^−1^]	*Ea* [kJ mol^−1^]
SSS/RRR	0.116	5.24 × 10^4^	2.46 × 10^6^	6.40 × 10^9^	29.5
SSR/RRS	0.224	2.52 × 10^1^	2.72 × 10^4^	2.13 × 10^10^	51.9
SRS/RSR	0.436	6.43 × 10^−1^	1.07 × 10^3^	2.13 × 10^9^	55.4
RSS/SRR	0.224	1.58 × 10^0^	2.31 × 10^2^	9.05 × 10^6^	39.1
*k_mig_* (weighted average)		6.08 × 10^3^	2.92 × 10^5^	7.85 × 10^8^	29.6
Van Steenberge et al. [54]			1.6 × 10^2^		
Ballard et al. [67]			3 × 10^3^		
Cuccato et al. [62]		6.24 × 10^0^	6.62 × 10^−4^	2.86 × 10^10^	63.3

**Table 6:** Activation energies and rate coefficients for the migration reaction by imposing a restriction on the distance between the first and last carbon atoms in the model structures. The values selected for the simulations are shown in bold.

Elongation between First and Last Carbon Atoms	G‡ 413.15 K [kJ mol^−1^ s^−1^]	k 413.15 K [s^−1^]
Non-strained	63.4	3.84 × 10^5^
8.4	64.8	2.55 × 10^5^
8.6	70.0	5.64 × 10^4^
**8.8**	**82.2**	**1.60 × 10^3^**
9.0	93.7	5.76 × 10^1^
9.2	113.5	1.80 × 10^−1^
9.4	139.1	1.03 × 10^−4^
9.6	171.3	8.87 × 10^−9^

**Table 7:** Macromonomer propagation Arrhenius parameters and comparison with ECR propagation.

Reaction	Source	*k* @ 413.15K [L mol^−1^ s^−1^]	*A* [L mol^−1^ s^−1^]	*E_a_* [kJ mol^−1^]
MM Propagation	This work	3.02 × 10^5^	3.97 × 10^7^	16.8
MM Propagation	Van Steenberge et al. [54]	2.5 × 10^5^		
MM Propagation	Wang et al. [30]	6.63 × 10^4^		
ECR Propagation	This work	2.18 × 10^5^	1.77 × 10^8^	23.0

**Table 9:** CTM kinetic parameters for the five different possibilities of hydrogen abstraction and the average sums.

CTM	*k* @ 333.15K [L mol^−1^ s^−1^]	*A* [L mol^−1^ s^−1^]	*E*_a_ [kJ mol^−1^]
V1	2.43 × 10^−8^	2.44 × 10^6^	89.1
V2	8.11 × 10^−8^	5.72 × 10^7^	94.7
A1	3.82 × 10^−2^	1.97 × 10^6^	49.0
A2	6.17 × 10^−2^	3.69 × 10^6^	49.5
A3	2.24 × 10^−2^	1.87 × 10^6^	50.3
A4	3.59 × 10^−4^	1.68 × 10^6^	61.5
k_CTM_	2.45 × 10^−1^	1.82 × 10^7^	49.6
Maeder and Gilbert [72]	2.24 × 10^0^	2.9 ± 0.9 × 10^5^	32.6 ± 0.8
Laki et al. [61]	1.48 × 10^1^	4.88 × 10^6^	35.2 ± 0.61

**Table 10:** Chain-transfer-to-polymer rate coefficients predicted via chirality considerations and inclusion of weighted averages for structures depending on ECR propagation rates.

Reaction	$\mathrm{Simulated}\;\omega_{i}$	*k* [s^−1^] 298.15K	*k* [s^−1^] 413.15K	*A* [s^−1^]	*E_a_* [kJ mol^−1^]
RRR/SSS	0.1156	1.72 × 10^−1^	1.92 × 10^1^	3.91 × 10^6^	42.0
RRS/SSR	0.4488	6.97 × 10^−4^	3.25 × 10^−1^	2.70 × 10^6^	54.7
RSR/SRS	0.4356	4.67 × 10^−4^	1.77 × 10^−1^	8.48 × 10^5^	52.9
Chain transfer to backbone ${\bar{k}}_{CTB}$		2.03 × 10^−2^	2.43 × 10^0^	5.97 × 10^5^	42.6
Chain transfer to alkyl branch $k_{CTA}$		3.32 × 10^−2^	8.47 × 10^0^	1.50 × 10^7^	49.5
${\bar{k}}_{CTP}$		5.20 × 10^−2^	1.07 × 10^1^	1.05 × 10^7^	47.4

**Table 11:** Reference data set and data sets used for three *k*MC simulations under different conditions. The pre-exponential factor units are L mol^−1^ s^−1^ or s^−1^ depending on the molecularity of the reaction. The activation energies units are kJ mol^−1^ K^−1^.

	$k_{p}$		$k_{BB}$		$k_{\beta SC}$		$k_{p,MCR}$	
	*A*	*E*_a_	*A*	*E*_a_	*A*	*E*_a_	*A*	*E*_a_
Data Set 1: reference set (Vir et al. [50])	2.2 × 10^7^	17.9	5.4 × 10^7^	30.6	7.9 × 10^12^	81.1	1.9 × 10^6^	30.1
Data Set 2: no special approaches	1.8 × 10^8^	23.0	3.1 × 10^10^	49.4	1.2 × 10^14^	112.9	3.7 × 10^6^	28.8
Data Set 3: all enantiomers considered for backbiting–weighted-average (WA) approach	1.8 × 10^8^	23.0	4.1 × 10^10^	46.8	1.2 × 10^14^	112.9	3.7 × 10^6^	28.8
Data Set 4: reduced flexibility applied to β-scission	1.8 × 10^8^	23.0	4.1 × 10^10^	46.8	2.8 × 10^13^	86.3	3.7 × 10^6^	28.8

**Table 12:** Rate coefficient comparison between Van Steenberge et al. [54] and this work for the electron spray ionization–mass spectrometry experiment. The simulation made with this work’s data set includes rate coefficients used by Van Steenberge et al. [54] for activation, deactivation and termination.

Reaction	Rate Used in Van Steenberge et al. [54] *k* [L mol^−1^ s^−1^] or [s^−1^] @ 413.15K	This Work’s Data Set *k* [L mol^−1^ s^−1^] or [s^−1^] @ 413.15K
Activation	4.0 × 10^3^	4.0 × 10^3^
Deactivation	1.0 × 10^6^	1.0 × 10^6^
Reduction	3.0 × 10^−1^	3.0 × 10^−1^
Backbiting	6.5 × 10^3^	9.4 × 10^4^
Migration	1.6 × 10^2^	1.6 × 10^3^
Chain transfer to polymer	6.0 × 10^2^	1.1 × 10^1^
β-scission	1.2 × 10^0^	3.4 × 10^2^
Macromonomer addition	2.5 × 10^5^	3.0 × 10^5^
Termination	1.0 × 10^8^	1.0 × 10^8^

## Data Availability

The data presented in this study is publicly available in the supporting information. All the information required to reproduce our results is the optimized geometries of reactants structures and transition states, which are included in the supporting information. All shown experimental data is extracted from previous work published elsewhere.

## Supplementary Materials

The following supporting information can be downloaded at: https://www.mdpi.com/article/10.3390/polym16070872/s1, Figure S1: Determination of the chirality in a pentamer end-chain radical; Figure S2: Methyl acrylate pentamer structure: RRRR/SSSS optical isomer straight vs curled; Figure S3: Transition state structure for the backbiting reaction; Figure S4: ESI-MS of the synthesis of macromonomers via activation of bromine-capped poly(n-butyl acrylate) including chain transfer to polymer multiplied by a factor of 1000; Table S1: Relative energies of all end-chain radicals optical isomers pentamer structures and their respective transition state for the backbiting reaction; Table S2: Predicted rate coefficients for the propagation reaction of methyl acrylate in bulk; Table S3: Relative energies of all end-chain radicals optical isomers pentamer structures and their respective transition state for the migration reaction; Table S4: β-scission of mid-chain radicals experimental and predicted published rate coefficients; Table S5: CTM rate coefficients published in literature; Table S6: Chain transfer to polymer rate coefficient and Arrhenius parameters from previously published results by different authors. Table S7: Effect of the solvation energy on different secondary reactions; List of optimized geometries.
