# Supplementary material for: Improved Approach for ab Initio Calculations of Rate Coefficients for Secondary Reactions in Acrylate Free-Radical Polymerization
Source: Polymers (Basel). 2024 Mar 22;16(7):872. doi: 10.3390/polym16070872 (PMC11013146; doi:10.3390/polym16070872)
Supplement: Supplementary file 1 [file polymers-16-00872-s001.zip › polymers-2908834-supplementary.pdf]

Supporting Information

An improved approach for *ab initio* calculations of rate coefficients for the secondary reactions in acrylate free-radical polymerization

Fernando A. Lugo, Mariya Edeleva, Paul H. M. Van Steenberge and Maarten K. Sabbe

Contents

Figure S1: Determination of the chirality in a pentamer end-chain radical 2

Figure S2. Methyl acrylate pentamer structure: RRRR/SSSS optical isomer straight (right) and curled (left) configuration. 3

Figure S3: Transition state structure for the backbiting reaction: In red those atoms belonging to the six-ring structure. 4

Table S1: Relative energies of all end-chain radicals optical isomers pentamer structures and their respective transition state for the backbiting reaction. All differences are based on the minimum energy optical isomer (RSRS/SRSR) 6

Table S2: Predicted rate coefficients for the propagation reaction of methyl acrylate in bulk. 7

Table S3: Relative energies of all end-chain radicals optical isomers pentamer structures and their respective transition state for the backbiting reaction. All differences are based on the minimum energy optical isomer (RSRS/SRSR) 8

Table S4. β-scission of mid-chain radicals experimental and predicted published rate coefficients. Dashed line divides experimental vs ab initio based results. Rate coefficients at 383 K are derived from the Arrhenius parameters if no k(383 K) was reported, these values are marked with the superscript Δ. 9

Table S5. CTM rate coefficients published in literature. 10

Table S6. Chain transfer to polymer rate coefficient and Arrhenius parameters from previously published results by different authors. Ordered by method and then chronologically. Dashed line divides experimental vs ab initio based results. 12

Table S7. Effect of the solvation energy on different secondary reactions. The interaction is given by the relation: $Ratio = kgaskcondensed$ for each reaction. 13

List of optimized geometries 14

Figure S1: Determination of the chirality in a pentamer end-chain radical

On the figure M_{1-4} represents the carbon atoms corresponding to each monomer unit in the chain that holds chirality. The priorities for assigning chirality to carbon M_1 are the following:

- 1st The carbon belonging to the acrylate side chain, as it has the closest oxygen group attached.
- 2nd The leftmost methyl group, as it represents the rest of the backbone of the chain.
- 3rd The carbon atom linking with the head of the chain
- 4th The hydrogen attached to M_1

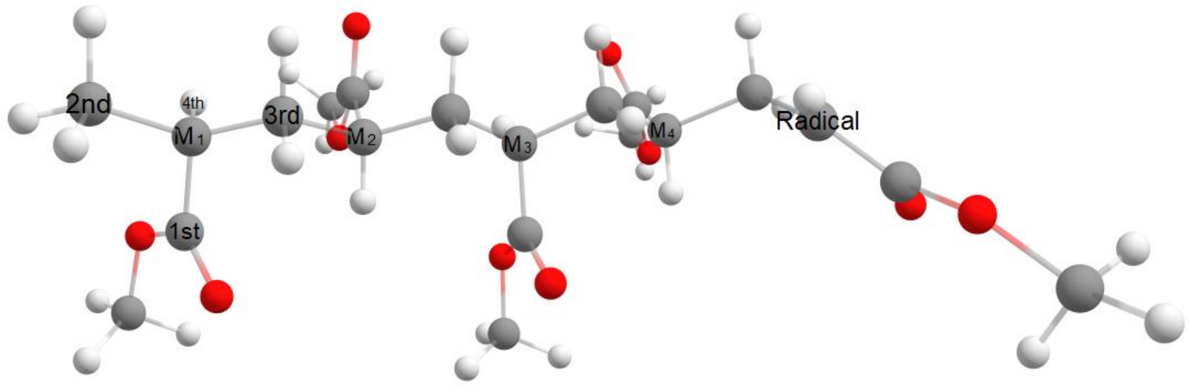

Following this set of rules applied to all carbon atoms on the backbone results in an optical isomer with the following chirality: SRSR-Radical.

Figure S2. Methyl acrylate pentamer structure: RRRR/SSSS optical isomer straight (right) and curled (left) configuration.

The following figure shows two configurations for the same methyl acrylate pentamer isomer: RRRR/SSSS. Usually, the configuration in which the backbone is a straight line (right) has less energy than the backbone in a "curled" shape (left). But, in this specific case, where the acrylate substituents are all in the same disposition, the steric repulsion between them makes it energetically more favorable to bend the main chain like in the left example, increasing the distance between acrylate units. This "curled" structure favors the backbiting reaction because the soon to be abstracted hydrogen atom is closer to the end chain radical.

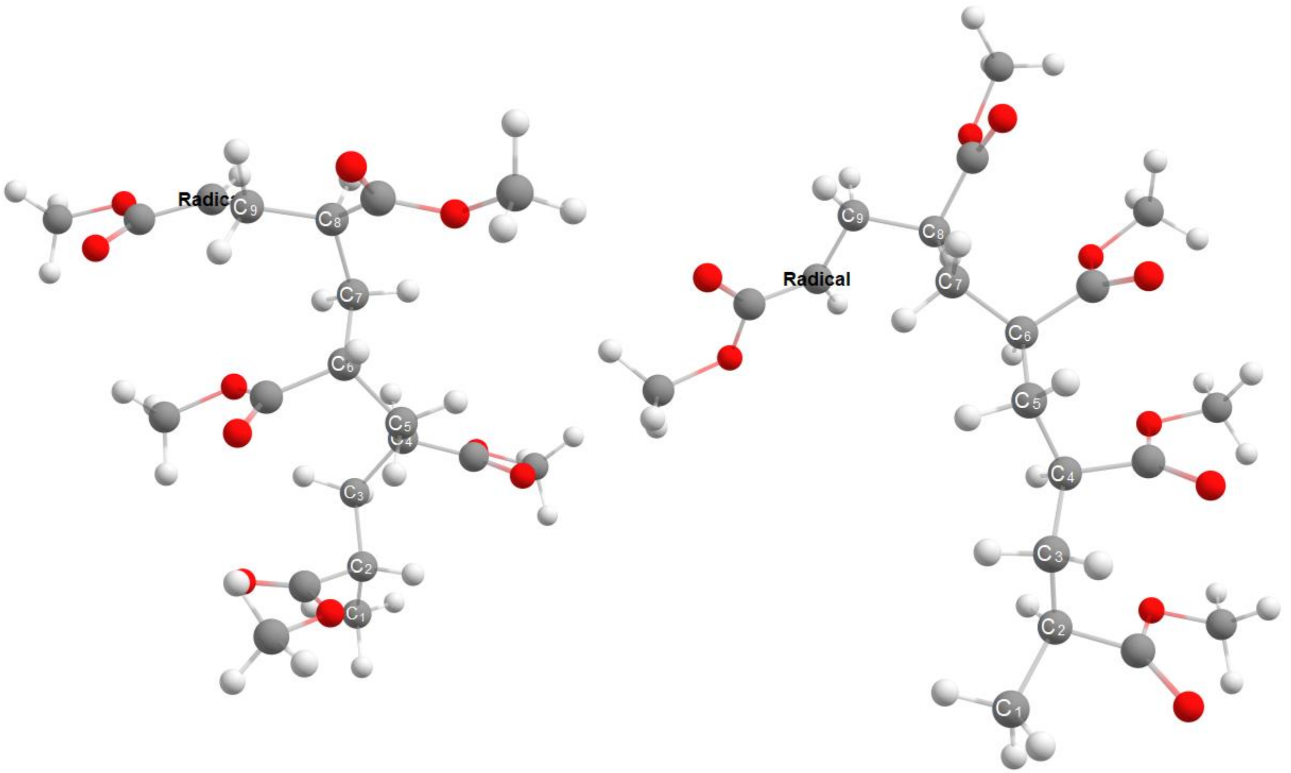

Figure S3: Transition state structure for the backbiting reaction: In red those atoms belonging to the six-ring structure.

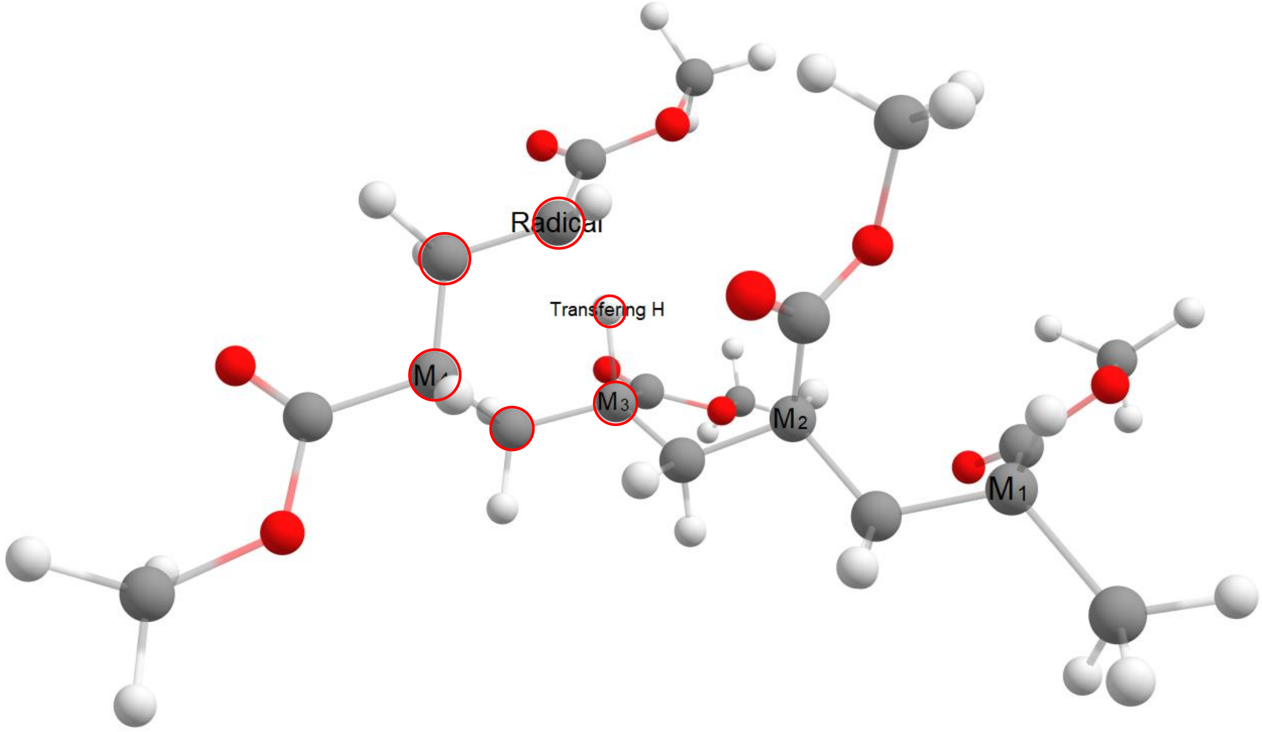

Side view version of the same molecule. This helps to visualize the disposition of acrylate substituents relative to the six-atom ring structure.

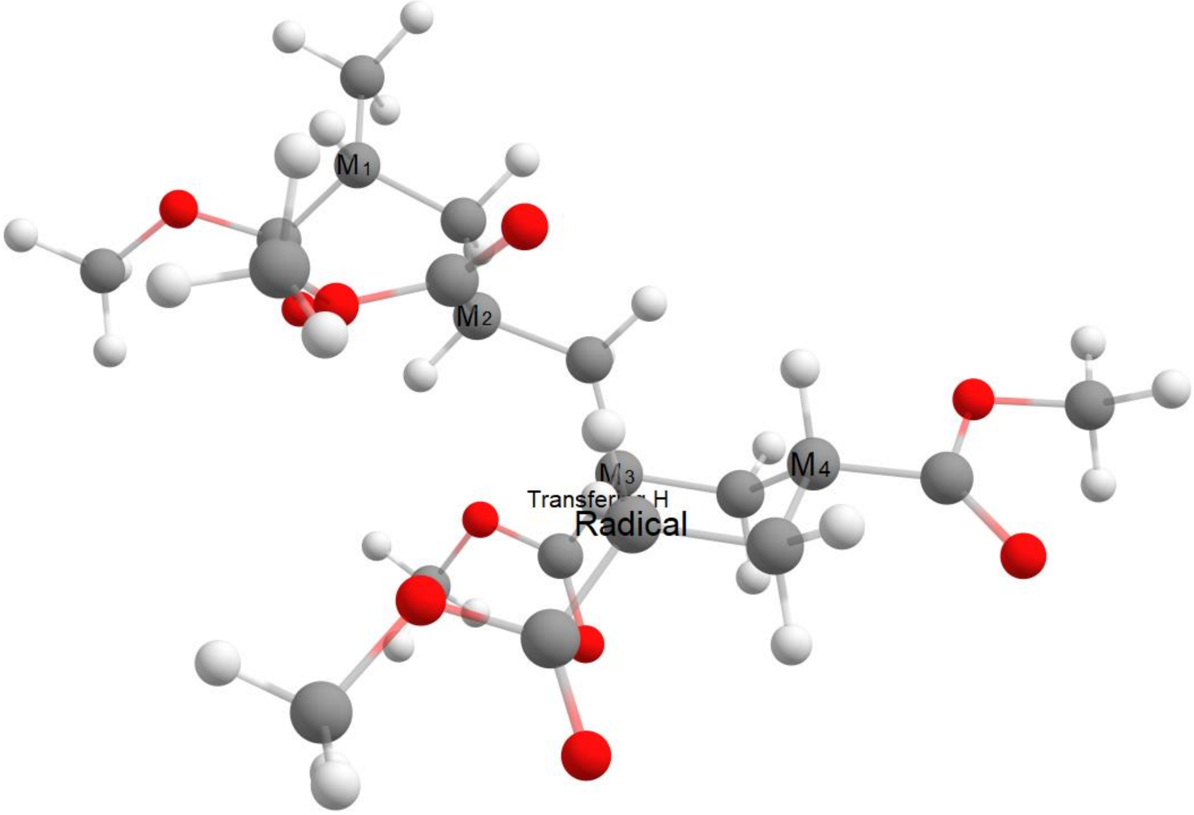

Figure S4: ESI-MS of the synthesis of macromonomers *via* activation of bromine-capped poly(*n*-butyl acrylate) including chain transfer to polymer multiplied by a factor of 1000.

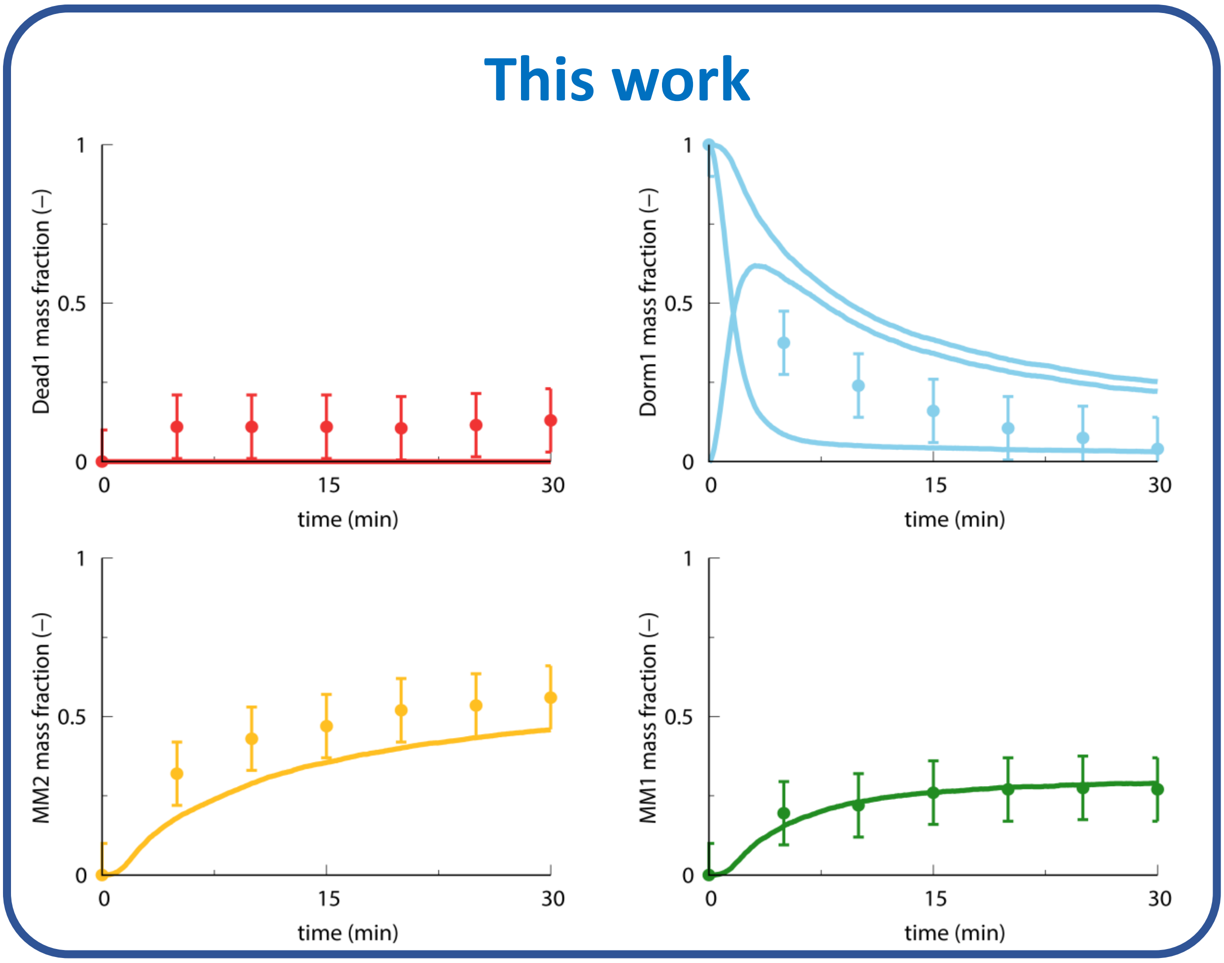

Table S1: Relative energies of all end-chain radicals optical isomers pentamer structures and their respective transition state for the backbiting reaction. All differences are based on the minimum energy optical isomer (RSRS/SRSR)

Optical Isomer	ΔElectronic Energy B3LYP/6-311+G(d,p) (kJ mol^{-1})	ΔElectronic Energy M062X/6-311+G(d,p) (kJ mol^{-1})	ΔGibbs free energy including COSMO-RS solvation effects BP86/TZVP (kJ mol^{-1})	ΔGibbs free energy including COSMO-RS solvation effects. For Transition States. BP86/TZVP (kJ mol^{-1})
RRRR/SSSS	14.62	12.24	4.19	1,35
RRRS/SSSR	18.40	29.67	11.10	5,32
RRSR/SSRS	11.05	23.35	7.16	4,42
RSRR/SRSS	14.89	15.76	3.44	6,10
SRRR/RSSS	15.02	22.98	7.43	0,00
RRSS/SSRR	12.81	19.60	6.51	2,36
RSRS/SRSR	0.00	0.00	0.00	2,28
RSSR/SRRS	7.48	22.59	4.23	0,19

Table S2: Predicted rate coefficients for the propagation reaction of methyl acrylate in bulk.

As a molecular model we used a dimer radical propagating to a monomer. Said dimer radical only chiral atom has chirality R. Hence, it can produce an RR trimer or an RS trimer. Table S2 shows the rate coefficient predicted for this reaction along with the reaction barrier for comparison.

Propagation reaction producing:	ΔG‡ (kJ/mol) 298.15K	k (L mol^{-1} S^{-1}) 298.15K	k (L mol^{-1} S^{-1}) 333.15K	k (L mol^{-1} S^{-1}) 413.15K
RR Trimer k_{IC}	64.99	7.22 x 10^2	1.66 x 10^3	6.91 x 10^3
RS Trimer k_{AC}	63.39	1.37 x 10^3	3.34 x 10^3	1.50 x 10^4

Table S3: Relative energies of all end-chain radicals optical isomers pentamer structures and their respective transition state for the backbiting reaction. All differences are based on the minimum energy optical isomer (RSRS/SRSR)

Optical Isomer	ΔElectronic Energy B3LYP/6-311+G(d,p) (kJ mol^{-1})	ΔElectronic Energy M062X/6-311+G(d,p) (kJ mol^{-1})	ΔGibbs free energy including COSMO-RS solvation effects BP86/TZVP (kJ mol^{-1})	ΔGibbs free energy including COSMO-RS solvation effects. For Transition States. BP86/TZVP (kJ mol^{-1})
RRR/SSS	22,2	14,9	18,9	0
RRS/SSR	14,6	11,7	13,8	19,2
RSR/SRS	0	0	0	29,5
SRR/RSS	11,9	4,8	5,9	28,1

Table S4. β-scission of mid-chain radicals experimental and predicted published rate coefficients. Dashed line divides experimental vs ab initio based results. Rate coefficients at 383 K are derived from the Arrhenius parameters if no k(383 K) was reported, these values are marked with the superscript Δ.

Year	Source	Acrylate/molecular model	Method	*A* s^{-1}	E_a kJ mol^{-1}	*k* 383K s^{-1}	*k* 411 or 413K s^{-1}
2004	Peck and Hutchinson [27]	Methyl	^{13}C and ^{1}H NMR	-	-	-	6.00×10^{0}
2009	Wang *et al.* [29]	Butyl	^{13}C and ^{1}H NMR - SEC	-	-	-	1.20×10^{1}
2010	Nikitin *et al.* [30]	Butyl	^{13}C and ^{1}H NMR - SEC	$1.49 \pm 0.28 \times 10^{9}$	63.9 ± 0.9	$2.80 \times 10^{0\Delta}$	1.37×10^{1}
2016	Hamzehlou *et al.* [31]	Butyl	^{13}C and ^{1}H NMR - SEC/MALS	$1.47 \pm 0.40 \times 10^{9}$	55.4	$4.09 \times 10^{1\Delta}$	1.45×10^{2}
2019	Vir *et al.* [33]	Butyl	PLP-SEC-*k*MC	7.92×10^{12}*	81.1 ± 18.2	$7.15 \pm 0.97 \times 10^{1}$	$4.34 \pm 0.41 \times 10^{2}$
2020	Laki *et al.* [75]	Ethyl	Gravimetry/GPC	9.64×10^{13}**	91.4 ± 1.8	$3.30 \times 10^{1\Delta}$	2.65×10^{2}
2013	Cuccato *et al.* [35, 36]	Tetramer methyl acrylate	B3LYP//MPWB1K /6-31G(d,p)	1.02×10^{14}	115.6	1.75×10^{-2}	2.44×10^{-1}
		Tetramer butyl acrylate		2.88×10^{12}	116.9	3.28×10^{-4}	4.73×10^{-3}
2013	Liu *et al.*[39]	Pentamer methyl acrylate	B3LYP//6-31G(d,p)	L 1.03×10^{14}<br>R 6.78×10^{14}	96.89<br>95.32	6.31×10^{0}<br>6.77×10^{1}	5.75×10^{1}<br>5.96×10^{2}

* Reported *A* value with an error margin as ln(A) = (29.7 ± 5.5), this translates to an error factor of 244.7.
** Reported *A* value with an error margin as ln(A) = (33.2 ± 0.49), this translates to an error factor of 1.63.

Table S5. CTM rate coefficients published in literature.

Chain transfer to monomer constants ($C_m = k_{CTM} k_p$) *and rate coefficients* (k_{CTM})*, including experimental and computational results. Dashed line divides experimental vs ab initio based results.*

Year	Source	Methods	Acrylate	Temperature °C	C_m	k L mol^{-1} s^{-1}	A L mol^{-1} s^{-1}	E_a kJ mol^{-1}
1955	Santhappa and Mahadevan [99]	Dried polymer weight and viscometry	methyl	55-70	1×10^{-6}	-		
1955	Mahadevan and Santhappa [100]	Dried polymer weight and viscometry	methyl	65	$1.1\text{-}10 \times 10^{-5}$*	-		
				70	$0.7\text{-}4.0 \times 10^{-5}$*			
				75	$2.2\text{-}2.5 \times 10^{-5}$*			
1957	Gopalan and Santhappa [101]	Dried polymer weight and viscometry	methyl	65	1.1×10^{-5}	-		
				75	2.5×10^{-5}			
1968	Patra and Mangaraj [102]	Dried polymer weight and viscometry	benzyl	55	8.83×10^{-5}	-		
				60	9.05×10^{-5}			
				65	9.55×10^{-5}			
1974	Rätzsch and Zschach [103]	Dried polymer weight and viscometry	ethyl	50	-	3.28×10^{-2}		
				60		5.53×10^{-2}		
				70		8.81×10^{-2}		
			2EHA	60	-	9.56×10^{-2}		
				65		1.28×10^{-1}		
				70		2.45×10^{-1}		
1981	Fehervari *et al.* [104]	Osmometry and viscometry	methyl	50	9.00×10^{-5}	-		
			ethyl		8.00×10^{-5}			
1996	Beuermann *et al.* [78]	PLP-SEC	butyl	40-80	$1.3 \pm 0.5 \times 10^{-4}$	-		
1998	Maeder and Gilbert [105]	SEC	butyl	60	$4.3\text{-}9.8 \times 10^{-5}$	$0.39\text{-}1.59 \times 10^{1}$	$2.9 \pm 0.9 \times 10^{5}$	32.6 ± 0.8
2019	Laki *et al.* [75]	Gravimetry/GPC	ethyl ECR	60		1.48×10^{1}	4.88×10^{6}**	35.2 ± 0.61

			MCR			2.55×10^{-3}	3.30×10^{2}***	32.6 ± 0.07
2013	Moghadam *et al.* [40]	M06-2X/6-311G(d, p)	butyl	60	3.49×10^{-6}	1.22×10^{-1}	2.86×10^{4}	34.2****
2014	Mavroudakis *et al.* [37]	B3LYP/(6-31G(d,p))// MPWB1K/(6-31G(d,p))	butyl	60	2.88×10^{-8}	1.01×10^{-3}	2.78×10^{6}	60.2****

* Depending on the used initiator: benzoyl peroxide, Di-Tert-Butyl peroxide, Methyl Ethyl ketone peroxide, Tertiary Butyl Hydroperoxide. The results show no clear trend between initiator and the rate coefficients should be independent from the initiator, so experimental error might be causing the differences.

** The reported *A* value has an error margin as $\ln(15.4 \pm 0.16)$, this translates to an error factor of 1.17.

*** The reported *A* value has an error margin as $\ln(5.8 \pm 0.02)$, this translates to an error factor of 1.02.

Table S6. Chain transfer to polymer rate coefficient and Arrhenius parameters from previously published results by different authors. Ordered by method and then chronologically. Dashed line divides experimental vs ab initio based results.

Year	Source	Method	*k* @333K L mol^{-1} s^{-1}	*k* @348K L mol^{-1} s^{-1}	*k* @353K L mol^{-1} s^{-1}	*k* @413K L mol^{-1} s^{-1}	*A* L mol^{-1} s^{-1}	E_a kJ mol^{-1}
2001	Plessis *et al.* [83]	Emulsion Polymerization/SEC		1.78×10^{-1}				
2003	Arzamendi *et al.* [71]	PLP/*k*MC	1.14×10^{-1}	1.79×10^{-1}	2.06×10^{-1}	8.64×10^{-1}	4.01×10^{3}	29
2006	Boschmann and Vana [28]	Z-RAFT Polymerization ^{13}C NMR	3.30×10^{-1}					
2016	Ballard *et al.* [106]	RAFT/SEC	1.09×10^{1}	2.13×10^{1}	2.64×10^{1}	2.25×10^{2}	6.70×10^{7}	43.3
2017	Van Steenberge *et al.* [92]	ESI-MS/*k*MC				6.0×10^{2}		
2014	Moghadam *et al.* [41]	M06-2X/6-311G(d,p) IEF-PCM in p-xylene and n-butanol	6.30×10^{-1}<br>4.43×10^{-2}	1.02×10^{0}<br>7.75×10^{-2}	1.19×10^{0}<br>9.24×10^{-2}	5.50×10^{0}<br>5.49×10^{-1}	4.57×10^{4}<br>1.95×10^{4}	31<br>36
		M06-2X/6-311G(d,p) COSMO in p-xylene and n-butanol	4.87×10^{0}<br>3.02×10^{0}	7.19×10^{0}<br>4.45×10^{0}	8.13×10^{0}<br>5.03×10^{0}	2.80×10^{1}<br>1.73×10^{1}	4.05×10^{4}<br>2.51×10^{4}	25<br>25

Table S7. Effect of the solvation energy on different secondary reactions. The interaction is given by the relation: $Ratio = \frac{k_{gas}}{k_{condensed}}$ for each reaction.

Reaction		Ratio between k at gas phase and k in condensed phase		
		298 K	333 K	413 K
Backbiting	RRRR	0.36	0.38	0.47
	RRRS	0.71	0.76	0.86
	RRSR	0.43	0.52	0.72
	RSRR	0.61	0.31	0.39
	SRRR	0.08	0.26	0.43
	RRSS	0.04	0.06	0.14
	RSRS	18.16	13.22	7.77
	RSSR	0.21	0.28	0.44
β-scission		1.60	1.29	1.23
Migration	RRR	2.43	2.28	2.03
	RRS	0.51	0.59	0.74
	RSR	0.86	0.90	0.97
	RSS	14.77	10.88	6.72
MM propagation		0.74	1.03	1.57
MCR propagation		0.85	1.19	1.86
Chain transfer to monomer	V2	0.39	0.58	0.94
	A1	0.23	0.36	0.68
	A2	0.47	0.85	1.12
	A3	0.38	0.54	0.93
	A4	1.17	1.40	1.78
	CTM	0.34	0.58	0.91
CTP	RRR	0.70	0.99	1.61
	RRS	0.25	0.40	0.79
	RSR	0.12	0.23	0.57

List of optimized geometries

The following section contains the optimized geometries for every reactant and transition state used within this work. This includes the 86 structures along with the B3LYP/6-311+G(d,p) electronic energy.

Methyl acrylate ECR pentamer with RRRR chirality
Standard orientation Energy= -1533.48782958

C	3.122754	-1.487156	1.349714
O	3.714771	-2.226425	0.600294
O	3.433002	-1.371619	2.661278
C	1.966418	-0.563513	0.977955
C	2.450917	0.910391	1.067178
H	1.591202	1.574690	0.975617
H	2.841638	1.059085	2.076353
C	4.534604	-2.174579	3.125805
H	4.629626	-1.951243	4.186191
H	5.450408	-1.912122	2.594278
H	4.324903	-3.234165	2.973716
H	1.230011	-0.684050	1.778233
C	2.930729	2.099201	-1.115408
O	2.290477	3.118367	-0.989797
O	3.196968	1.532105	-2.304325
C	3.544202	1.328049	0.045365
C	2.627560	2.183506	-3.463274
H	2.981323	1.608505	-4.316105
H	2.973999	3.215772	-3.523434
H	1.539831	2.157608	-3.402177
H	4.037789	0.443625	-0.363630
C	4.607495	2.226016	0.707532
H	4.146422	3.128447	1.115516
H	5.097390	1.687046	1.522252
H	5.375385	2.528985	-0.009204
C	-0.465691	0.687484	-0.913423
O	-0.368228	1.131957	-2.034225
O	-0.800417	1.435189	0.152346
C	-0.175775	-0.770630	-0.572771
C	1.349588	-1.007929	-0.366246
H	1.501188	-2.084960	-0.462408
H	1.893909	-0.563942	-1.202786
C	-1.044539	2.841713	-0.108260
H	-1.232713	3.280244	0.869533
H	-1.919925	2.949160	-0.747418
H	-0.166876	3.290006	-0.571780
C	-2.354942	-2.939140	-0.969499
O	-3.057914	-2.970466	-1.949462
O	-1.411975	-3.870068	-0.709064
C	-2.427339	-1.896453	0.144447
C	-1.027078	-1.382392	0.561638
H	-1.153955	-0.659650	1.370220
H	-0.480318	-2.232774	0.974228
C	-1.281710	-4.928857	-1.679189
H	-0.479376	-5.563109	-1.309251
H	-1.028607	-4.519293	-2.657659
H	-2.213475	-5.490651	-1.755495
H	-2.817387	-2.449923	1.009891
C	-4.373346	1.331674	0.874739
O	-4.344397	2.016254	-0.133760
O	-4.847897	1.790238	2.066933
C	-3.925472	-0.040609	0.973678
C	-3.449777	-0.799678	-0.208887
H	-3.071997	-0.114081	-0.971617
H	-4.313498	-1.286866	-0.686660
C	-5.336595	3.140132	2.066385
H	-5.674230	3.328472	3.083416
H	-6.163020	3.249104	1.361830
H	-4.543214	3.837578	1.791569
H	-4.021536	-0.528037	1.938094
H	-0.402034	-1.291099	-1.507935

Methyl acrylate ECR pentamer with RRRS chirality
Standard orientation Energy= -1533.49495313

C	2.886028	-0.316908	-0.704393
O	3.336969	0.251540	-1.668334
O	3.059784	-1.635477	-0.470599
C	2.052467	0.347136	0.382565
C	2.714274	1.652231	0.872721
H	2.758775	2.376852	0.054931
H	2.050202	2.073341	1.633774
H	1.983156	-0.347740	1.224834
C	5.213712	1.655767	0.452926
O	5.443394	2.676175	-0.148074
O	5.929085	0.521280	0.289110
C	4.121600	1.499325	1.503241
C	6.964155	0.578930	-0.712024
H	7.445292	-0.396838	-0.689653
H	7.681541	1.365416	-0.474732
H	6.525489	0.773067	-1.691505
H	4.217123	0.503816	1.945329
C	4.349200	2.557716	2.597912
H	4.260507	3.563236	2.180936
H	3.608260	2.441303	3.392891
H	5.342867	2.462678	3.043566
C	-0.094057	-1.697424	-0.837668
O	0.206481	-1.664759	-2.006975
O	-0.245263	-2.847104	-0.143694
C	-0.409865	-0.483959	0.022750
C	0.623738	0.645579	-0.147986
H	0.678950	0.946111	-1.197884
H	0.239486	1.507067	0.406273
C	-0.016817	-4.065552	-0.876580
H	-0.209343	-4.870205	-0.170472
H	1.013417	-4.105716	-1.231264
H	-0.692767	-4.132372	-1.730211
H	-0.419849	-0.803357	1.067183
C	-3.151234	-1.301487	1.335486
O	-2.823961	-0.612367	2.273210
O	-3.767548	-2.492862	1.473264
C	-2.977339	-0.938711	-0.132182
C	-1.817742	0.044192	-0.347663
H	-1.812923	0.345585	-1.399789
H	-2.002195	0.942813	0.246796
C	-4.030950	-2.920242	2.824376
H	-4.518132	-3.888253	2.732097
H	-4.684426	-2.207738	3.329526
H	-3.098086	-3.010399	3.381989
H	-2.793089	-1.871725	-0.670545
C	-5.397831	1.935367	-0.851274
O	-5.560862	1.901446	-2.059215
O	-5.788689	2.984034	-0.079041
C	-4.776234	0.891470	-0.061546

C	-4.315298	-0.372999	-0.692151
H	-4.221672	-0.224552	-1.770866
H	-5.089223	-1.144373	-0.558183
C	-6.411222	4.074736	-0.776277
H	-6.648885	4.811022	-0.011459
H	-7.318769	3.739326	-1.281318
H	-5.728687	4.496599	-1.516108
H	-4.688754	1.046372	1.007771
C	3.790981	-2.355724	-1.481621
H	3.855494	-3.380071	-1.120000
H	3.257926	-2.311889	-2.432128
H	4.787808	-1.931278	-1.602676

Methyl acrylate ECR pentamer with RRSR chirality
Standard orientation Energy= -1533.49815306

C	2.591281	-0.525794	-0.531638
O	2.918559	-0.436257	-1.690616
O	2.601293	-1.689015	0.154062
C	2.069268	0.620553	0.320770
C	2.906294	1.903142	0.155876
H	2.902417	2.219563	-0.891089
H	2.396412	2.685788	0.725637
H	2.106766	0.306698	1.367477
C	5.300458	1.302016	-0.412578
O	5.632826	1.918281	-1.394827
O	5.738466	0.052382	-0.139031
C	4.367607	1.831108	0.668340
C	6.591833	-0.539046	-1.138357
H	6.869243	-1.514555	-0.743808
H	7.478389	0.076318	-1.295793
H	6.050175	-0.640210	-2.079640
H	4.415759	1.146944	1.519998
C	4.860014	3.220625	1.107125
H	4.821191	3.921975	0.271032
H	4.232213	3.602938	1.915840
H	5.891628	3.182240	1.466530
C	-0.509612	-0.441297	1.746752
O	-0.520389	0.432521	2.582004
O	-0.626920	-1.751980	2.052059
C	-0.406327	-0.220488	0.245064
C	0.596757	0.904584	-0.066216
H	0.554654	1.122982	-1.137433
H	0.278279	1.805891	0.464699
C	-0.754932	-2.068519	3.452006
H	-0.838591	-3.151978	3.498216
H	-1.643587	-1.595915	3.872882
H	0.124496	-1.727594	3.999559
H	-0.084002	-1.157047	-0.211982
C	-2.629709	-2.201037	-0.770877
O	-1.873282	-2.371647	-1.695871
O	-3.363900	-3.191377	-0.221146
C	-2.920883	-0.867307	-0.097349
C	-1.795533	0.150605	-0.329834
H	-1.678262	0.301688	-1.406243
H	-2.105504	1.108982	0.098989
C	-3.209704	-4.496944	-0.811364
H	-3.869925	-5.150792	-0.245952
H	-2.174623	-4.830496	-0.728456
H	-3.497131	-4.476328	-1.863357
H	-3.043940	-1.070701	0.970586
C	-5.238075	2.044188	-0.570185
O	-5.225966	2.215171	-1.777052
O	-5.689786	2.978713	0.308134
C	-4.784111	0.847937	0.110377
C	-4.284077	-0.334617	-0.634554
H	-4.205001	-0.092506	-1.697280
H	-5.019880	-1.145875	-0.538958
C	-6.158067	4.206502	-0.272507
H	-6.471770	4.823895	0.566475
H	-6.996738	4.017463	-0.944839
H	-5.358444	4.695953	-0.831201
H	-4.843698	0.827649	1.193252
C	3.014434	-2.854661	-0.584178
H	2.937132	-3.683860	0.115650
H	2.361254	-3.013448	-1.443192
H	4.042384	-2.739306	-0.929351

Methyl acrylate ECR pentamer with RSRR chirality
Standard orientation Energy= -1533.49877398

C	2.010146	0.720831	1.369234
O	1.532104	1.635978	1.999093
O	2.622965	-0.332244	1.952122
C	2.008471	0.609715	-0.147361
C	2.866476	1.742418	-0.753694
H	2.825562	1.650215	-1.842353
H	2.413873	2.701975	-0.485034
H	2.439634	-0.353602	-0.425910
C	5.090135	0.540116	-0.806137
O	4.904297	-0.018267	-1.860259
O	6.045701	0.163177	0.071983
C	4.338705	1.768102	-0.315356
C	6.860236	-0.956248	-0.325212
H	7.557877	-1.113333	0.494596
H	7.396326	-0.730255	-1.248103
H	6.241531	-1.841235	-0.479019
H	4.409763	1.782995	0.775829
C	5.052959	3.024988	-0.855035
H	5.022815	3.048048	-1.947547
H	4.559084	3.924421	-0.479442
H	6.097791	3.053804	-0.537619
C	0.038747	-1.757121	-0.593426
O	0.578636	-2.016797	-1.639149
O	-0.252313	-2.689565	0.343217
C	-0.407092	-0.368714	-0.155560
C	0.565112	0.702020	-0.683256
H	0.603475	0.638113	-1.773783
H	0.163486	1.684713	-0.419643
C	0.075026	-4.047837	-0.010840
H	-0.205643	-4.648760	0.851913
H	1.142888	-4.141050	-0.211412
H	-0.486663	-4.349426	-0.895780
H	-0.415270	-0.350397	0.938793
C	-3.208274	-2.198443	-0.303876
O	-2.931856	-2.665247	-1.381459
O	-3.829192	-2.898198	0.670742
C	-2.984937	-0.755572	0.124930
C	-1.847365	-0.066085	-0.646088
H	-1.934456	-0.299193	-1.710979
H	-1.990583	1.013580	-0.545631
C	-4.182101	-4.257061	0.349918
H	-4.676116	-4.646259	1.237445
H	-3.288674	-4.839643	0.122440
H	-4.854930	-4.284461	-0.508287
H	-2.760690	-0.768162	1.196167
C	-4.807954	2.520952	-0.144141
O	-5.176119	2.545532	-1.305856
O	-4.778065	3.625190	0.649184
C	-4.352212	1.342305	0.565594
C	-4.336348	0.000926	-0.069621
H	-4.569707	0.093116	-1.133185
H	-5.130497	-0.608368	0.385483
C	-5.209471	4.848740	0.032517

H	-5.116352	5.611280	0.802819
H	-6.244997	4.765380	-0.302034
H	-4.577996	5.089903	-0.824362
H	-4.049093	1.460493	1.600388
C	2.689855	-0.311638	3.391191
H	3.213941	-1.222919	3.670280
H	3.237068	0.567023	3.735164
H	1.686605	-0.298839	3.819198

Methyl acrylate ECR pentamer with SRRR chirality
Standard orientation Energy= -1533.49658317

C	2.696000	-1.471485	-0.814027
O	3.241180	-1.840596	-1.825234
O	2.544986	-2.259223	0.274532
C	2.108953	-0.084675	-0.591110
C	3.001583	0.976405	-1.261678
H	3.065783	0.755166	-2.330494
H	2.517736	1.950550	-1.150982
H	2.073725	0.099520	0.486053
C	4.429199	1.540522	0.743750
O	3.603098	2.261171	1.253551
O	5.509582	1.075742	1.408655
C	4.428680	1.074981	-0.704079
C	5.648970	1.508728	2.774788
H	6.562533	1.041325	3.135695
H	4.792145	1.184441	3.366904
H	5.727609	2.595692	2.825014
H	4.914006	0.096119	-0.737394
C	5.284626	2.053315	-1.536250
H	5.337409	1.708774	-2.571569
H	4.846551	3.055286	-1.530912
H	6.302909	2.118550	-1.147049
C	-0.482156	-2.113734	-0.358517
O	-0.128277	-2.767205	-1.307615
O	-0.999626	-2.661207	0.765880
C	-0.446560	-0.595315	-0.263306
C	0.662438	0.010128	-1.145140
H	0.618689	-0.429102	-2.145505
H	0.443459	1.075846	-1.254981
C	-1.156697	-4.092725	0.748674
H	-1.585421	-4.349776	1.715235
H	-0.189875	-4.580256	0.619605
H	-1.820761	-4.388614	-0.064368
H	-0.256887	-0.337956	0.784007
C	-3.692842	-1.548762	0.110250
O	-3.688740	-2.195114	-0.908500
O	-4.399841	-1.899749	1.206952
C	-2.982263	-0.220310	0.327115
C	-1.823212	0.007919	-0.658593
H	-2.115153	-0.350414	-1.649680
H	-1.671939	1.087045	-0.745356
C	-5.199291	-3.092191	1.092467
H	-5.693851	-3.203861	2.054863
H	-4.568717	-3.957031	0.883197
H	-5.933586	-2.984195	0.293016
H	-2.611780	-0.221059	1.356740
C	-3.678333	3.429269	-0.110361
O	-4.128076	3.483247	-1.242063
O	-3.210924	4.518485	0.556163
C	-3.567968	2.226448	0.690787
C	-4.053645	0.910295	0.206590
H	-4.393767	0.999755	-0.827979
H	-4.920864	0.615410	0.813606
C	-3.274498	5.763118	-0.158512
H	-2.862739	6.508509	0.518453
H	-4.307249	6.005765	-0.414797
H	-2.684370	5.709763	-1.075042
H	-3.140190	2.317909	1.683437
C	3.007225	-3.616486	0.129284
H	2.824956	-4.090035	1.091953
H	2.450213	-4.117078	-0.663523
H	4.071277	-3.633130	-0.109504

Methyl acrylate ECR pentamer with RRSS chirality
Standard orientation Energy= -1533.49742499

C	2.528348	-0.075304	-1.082937
O	2.089580	0.325883	-2.133443
O	3.460779	-1.050254	-0.996086
C	2.117411	0.430889	0.292392
C	2.994855	1.634095	0.728868
H	3.044069	2.369564	-0.079461
H	2.472019	2.112362	1.562405
C	3.968468	-1.542179	-2.251992
H	4.680271	-2.322856	-1.991320
H	4.457719	-0.735155	-2.798612
H	3.157815	-1.950222	-2.856751
H	2.274894	-0.378092	1.010852
C	5.392040	1.049975	0.077267
O	5.320884	1.516363	-1.034626
O	6.418135	0.272302	0.487182
C	4.416426	1.306910	1.214742
C	7.464773	0.043869	-0.473269
H	8.195800	-0.580453	0.035968
H	7.914693	0.988877	-0.781272
H	7.072322	-0.467463	-1.353150
H	4.398506	0.408270	1.838151
C	4.982999	2.467328	2.064350
H	5.010859	3.394410	1.485152
H	4.350906	2.631433	2.940260
H	5.994249	2.245789	2.411268
C	-0.261200	-1.378976	1.062878
O	-0.033716	-1.179492	2.231290
O	-0.462180	-2.613825	0.549911
C	-0.358449	-0.303289	-0.009830
C	0.632568	0.836443	0.288535
H	0.487539	1.619439	-0.460273
H	0.391232	1.259286	1.267277
C	-0.453536	-3.694874	1.503134
H	-0.604586	-4.599432	0.917464
H	-1.257343	-3.560584	2.227963
H	0.502770	-3.733386	2.025906
H	-0.111238	-0.760278	-0.971693
C	-3.353388	-1.771759	0.021023
O	-3.451008	-1.771748	1.225400
O	-3.779093	-2.790667	-0.755503
C	-2.827504	-0.625613	-0.828218
C	-1.801139	0.259519	-0.102390
H	-1.735871	1.198964	-0.658910
H	-2.165565	0.508034	0.898377
C	-4.390641	-3.900505	-0.071881
H	-4.667321	-4.605479	-0.852648
H	-5.273098	-3.571401	0.478746
H	-3.684878	-4.356495	0.623236
H	-2.372191	-1.065970	-1.719185
C	-5.269448	2.269134	-0.429385
O	-5.043209	2.974092	-1.398766
O	-5.998597	2.690104	0.638509
C	-4.810289	0.905582	-0.259639
C	-4.042541	0.216004	-1.328961
H	-3.693236	0.953897	-2.054967
H	-4.715759	-0.460521	-1.877124
C	-6.492313	4.036552	0.565588

H	-7.040447	4.195938	1.491757
H	-7.151219	4.158033	-0.296044
H	-5.665792	4.744567	0.482510
H	-5.064360	0.394067	0.661523

Methyl acrylate ECR pentamer with RSRS chirality
Standard orientation Energy= -1533.50106815

O	-6.060426	0.689064	0.784365
C	-4.685515	1.086386	-1.106351
C	-6.451884	0.911399	2.152144
H	-7.340067	0.301670	2.302788
H	-6.676763	1.965808	2.318771
H	-5.655083	0.603712	2.830522
H	-5.003538	0.060450	-1.314557
C	-5.562556	2.031175	-1.955462
H	-5.299841	3.076183	-1.769589
H	-5.410182	1.825299	-3.017612
H	-6.622185	1.895084	-1.730376
C	0.371364	0.444322	1.020576
O	0.704321	1.579197	1.268970
O	0.000853	-0.442623	1.966195
C	0.314882	-0.152396	-0.378969
C	-0.780251	0.564941	-1.199342
H	-0.591022	1.641611	-1.158692
H	-0.692149	0.252627	-2.243731
C	0.001021	0.043633	3.323919
H	-0.336426	-0.793483	3.930817
H	-0.678100	0.891123	3.423215
H	1.005740	0.350741	3.616988
H	0.070097	-1.213826	-0.292129
C	2.664169	-2.188892	-0.284148
O	2.216763	-2.872067	-1.174586
O	3.098328	-2.688023	0.889506
C	2.856778	-0.680984	-0.354301
C	1.683061	-0.000668	-1.074903
H	1.899022	1.066323	-1.171306
H	1.598616	-0.419341	-2.081541
C	3.025471	-4.119666	1.036003
H	3.421601	-4.329423	2.026916
H	3.625671	-4.611671	0.269537
H	1.991624	-4.457626	0.954710
H	2.952752	-0.305480	0.667092
C	5.766779	1.436910	-0.265084
O	6.437951	0.727144	0.464386
O	5.989591	2.769787	-0.413419
C	4.650267	0.989352	-1.075042
C	4.223536	-0.432059	-1.079586
H	4.995008	-1.030395	-0.590584
H	4.116712	-0.785389	-2.113030
C	7.072479	3.311659	0.358512
H	7.101662	4.371565	0.114949
H	8.014312	2.829844	0.089969
H	6.891744	3.168577	1.425314
H	4.131463	1.733794	-1.666332
C	-2.612131	-1.141038	-1.037243
O	-2.532180	-1.661478	-2.124796
O	-3.085263	-1.780169	0.054109
C	-2.225076	0.299066	-0.732481
C	-3.198921	1.261687	-1.448778
H	-2.898654	2.284109	-1.203536
H	-3.082076	1.134210	-2.529601
C	-3.489776	-3.149483	-0.141556
H	-3.804256	-3.501950	0.838240
H	-2.654918	-3.742948	-0.515897
H	-4.315555	-3.205421	-0.852386
H	-2.306806	0.455366	0.344806

C	-4.962233	1.354029	0.365269
O	-4.322085	2.088247	1.079921

Methyl acrylate ECR pentamer with RSSR chirality
Standard orientation Energy= -1533.49945439

C	2.390467	-1.453005	-0.993791
O	2.385626	-2.652474	-1.131071
O	2.703978	-0.597064	-1.991500
C	2.068430	-0.729054	0.306749
C	2.911243	-1.332611	1.449823
H	2.647426	-0.812298	2.374501
H	2.634098	-2.384245	1.567903
C	2.978799	-1.204722	-3.269501
H	3.246521	-0.383042	-3.930800
H	2.091846	-1.723909	-3.634658
H	3.804203	-1.912532	-3.186211
H	2.325147	0.326463	0.191254
C	4.919624	0.185306	1.227171
O	4.415719	1.111267	1.817569
O	6.035464	0.310307	0.475580
C	4.432746	-1.255264	1.258739
C	6.621509	1.624791	0.426768
H	7.495825	1.529331	-0.213420
H	6.910984	1.951448	1.426597
H	5.914363	2.341528	0.007185
H	4.718280	-1.720994	0.311445
C	5.171909	-1.999426	2.391009
H	4.930707	-1.563519	3.364326
H	4.870762	-3.049568	2.402544
H	6.254266	-1.957482	2.251591
C	-0.363562	0.246630	-1.464902
O	-0.557170	-0.722177	-2.160995
O	-0.176292	1.488550	-1.962485
C	-0.351768	0.247217	0.055278
C	0.557213	-0.854068	0.635254
H	0.447757	-0.806336	1.722549
H	0.197054	-1.838446	0.323226
C	-0.197357	1.608294	-3.396986
H	-0.061185	2.667753	-3.602373
H	-1.149359	1.257625	-3.798067
H	0.613272	1.026003	-3.835995
H	0.014508	1.219906	0.390267
C	-2.461785	2.482493	0.747470
O	-1.829043	2.663117	1.759263
O	-2.993203	3.484595	0.015857
C	-2.806227	1.125126	0.150467
C	-1.796721	0.048578	0.572044
H	-1.763162	0.011503	1.664094
H	-2.158157	-0.923461	0.223130
C	-2.773386	4.818535	0.515956
H	-3.263090	5.479352	-0.195803
H	-1.705261	5.032364	0.568231
H	-3.210928	4.930214	1.508870
H	-2.814003	1.244121	-0.937259
C	-5.411753	-1.533564	0.627852
O	-5.530372	-1.594349	1.839721
O	-5.862874	-2.502839	-0.212111
C	-4.786186	-0.447062	-0.098899
C	-4.253515	0.753038	0.593645
H	-4.291829	0.600702	1.675083
H	-4.904515	1.608806	0.362806
C	-6.491532	-3.631055	0.416366
H	-6.780391	-4.293364	-0.396906
H	-7.368425	-3.314849	0.984092
H	-5.794262	-4.132509	1.089785
H	-4.736001	-0.523093	-1.179673

Methyl acrylate pentamer backbiting TS with RRRR chirality
Standard orientation Energy= -1533.46934180

C	-0.737286	-2.344514	-0.804750
O	-0.264748	-2.142667	-1.899641
O	-0.506867	-3.472217	-0.101192
C	-1.639354	-1.394158	-0.031422
C	-2.314265	-0.379505	-0.970756
H	-1.576159	0.315370	-1.372827
H	-2.725062	-0.923406	-1.825076
C	0.367054	-4.444650	-0.711671
H	0.354491	-5.302333	-0.042992
H	1.378831	-4.045462	-0.797294
H	-0.003064	-4.719453	-1.699571
H	-2.416199	-2.028869	0.405276
C	-4.585577	-0.419811	0.147442
O	-4.904523	-1.488089	-0.320527
O	-5.257069	0.166091	1.163669
C	-3.436086	0.452306	-0.330170
C	-6.414308	-0.539257	1.649774
H	-6.813529	0.076628	2.452689
H	-6.132193	-1.523920	2.025107
H	-7.151264	-0.658491	0.854184
H	-3.057037	1.007911	0.530770
C	-3.989792	1.483152	-1.337980
H	-3.184150	2.143345	-1.666720
H	-4.404323	0.984301	-2.218429
H	-4.771452	2.096408	-0.884893
C	0.345572	1.377776	1.624065
O	-0.637024	2.091382	1.631858
O	1.516564	1.734096	2.188138
C	0.396728	0.032821	0.959585
C	-0.894482	-0.766454	1.183607
H	-0.651912	-1.583356	1.872390
H	-1.611131	-0.124614	1.695477
C	1.585496	3.067665	2.728888
H	2.563297	3.137953	3.200162
H	0.790699	3.226372	3.458054
H	1.498552	3.798189	1.923758
C	4.058814	-1.116619	0.319313
O	4.015410	-2.325568	0.335211
O	5.212395	-0.419924	0.356217
C	2.854224	-0.199584	0.218752
C	1.694690	-0.761984	1.065567
H	1.509616	-1.793131	0.758772
H	2.007258	-0.803280	2.115605
C	6.423770	-1.198762	0.393474
H	7.233502	-0.473029	0.419101
H	6.499674	-1.828644	-0.493947
H	6.443573	-1.829518	1.283140
H	3.140786	0.787263	0.578618
C	1.257555	2.177515	-1.514054
O	2.148301	2.843303	-1.024721
O	0.171345	2.722838	-2.115452
C	1.180338	0.702253	-1.500488
C	2.456019	-0.082389	-1.280827
H	3.282554	0.393369	-1.819302
H	2.316425	-1.084827	-1.691049
C	0.105803	4.159332	-2.115315
H	-0.791687	4.409622	-2.677034
H	0.989038	4.583967	-2.594324
H	0.032941	4.536027	-1.093723
H	0.496298	0.284543	-2.231757
H	0.511890	0.436310	-0.330842

Methyl acrylate pentamer backbiting TS with RRRS chirality
Standard orientation Energy= -1533.46841136

C	2.254165	0.395862	-1.583131
O	2.275220	0.274568	-2.782146
O	3.157252	1.124265	-0.893879
C	1.259484	-0.278502	-0.644318
C	1.802638	-1.643760	-0.143801
H	0.972962	-2.141182	0.368475
H	2.069915	-2.273474	-0.998002
C	4.145531	1.802056	-1.691496
H	4.781906	2.327008	-0.981690
H	3.660991	2.509054	-2.365873
H	4.721636	1.082183	-2.274501
H	1.147908	0.372605	0.225574
C	4.326705	-1.437505	0.158597
O	4.597980	-1.813331	-0.956198
O	5.233854	-0.881515	0.991932
C	2.979359	-1.594129	0.846319
C	6.576676	-0.776314	0.485588
H	7.154262	-0.317971	1.285560
H	6.600855	-0.153420	-0.409381
H	6.972883	-1.763551	0.243022
H	2.859359	-0.745752	1.525621
C	3.035438	-2.885466	1.694003
H	2.116380	-2.991638	2.275583
H	3.138025	-3.766098	1.053746
H	3.876550	-2.864908	2.389846
C	-0.908641	1.948582	-1.095470
O	0.186931	2.395676	-1.362516
O	-1.979487	2.733284	-0.845478
C	-1.223353	0.494575	-0.943045
C	-0.106697	-0.458069	-1.354105
H	-0.464897	-1.477668	-1.186416
H	0.049278	-0.370146	-2.435125
C	-1.753974	4.153217	-0.905232
H	-2.711431	4.607721	-0.661376
H	-1.431160	4.445317	-1.905428
H	-0.993284	4.449079	-0.181548
H	-1.321412	0.373744	0.381050
C	-4.625000	-1.391589	-0.793949
O	-5.380564	-0.711069	-1.443101
O	-4.949183	-2.613441	-0.317131
C	-3.217344	-1.002569	-0.375233
C	-2.629152	0.045942	-1.342115
H	-2.594371	-0.377525	-2.353173
H	-3.305823	0.897665	-1.378131
C	-6.285321	-3.071529	-0.604751
H	-6.357920	-4.056968	-0.150218
H	-6.442278	-3.132084	-1.682302
H	-7.021747	-2.392861	-0.172501
H	-2.617890	-1.918599	-0.389013
C	-1.677346	0.935352	2.554035
O	-2.509941	1.763127	2.857303
O	-0.428201	0.906811	3.088954
C	-1.846783	-0.133559	1.550813
C	-3.241166	-0.462198	1.078962
H	-3.862334	0.435497	1.133690
H	-3.693855	-1.214712	1.734968
C	-0.133678	1.936270	4.049503
H	0.893325	1.758350	4.361090
H	-0.810690	1.868733	4.902433
H	-0.229875	2.923422	3.594827
H	-1.177381	-0.978823	1.700238

Methyl acrylate pentamer backbiting TS with RRSR chirality
Standard orientation Energy= -1533.46643089

C	1.581499	0.430970	0.280668

O	1.261122	1.572847	0.064647
O	2.281261	0.050982	1.371608
C	1.302064	-0.741279	-0.645300
C	2.469471	-0.924408	-1.654835
H	2.105624	-1.597934	-2.436634
H	2.693102	0.030378	-2.139440
C	2.648025	1.113462	2.272667
H	3.171445	0.629608	3.095131
H	1.756570	1.627549	2.633858
H	3.298556	1.827519	1.765950
H	1.241983	-1.644767	-0.033835
C	4.649414	-0.520046	-0.389342
O	4.690683	0.664472	-0.620193
O	5.459260	-1.127351	0.505439
C	3.767671	-1.535233	-1.100176
C	6.422130	-0.284650	1.164123
H	6.980709	-0.943232	1.825717
H	5.919948	0.496201	1.736496
H	7.086551	0.179985	0.434109
H	3.532624	-2.323237	-0.378836
C	4.604275	-2.162386	-2.238966
H	4.036365	-2.959090	-2.725440
H	4.854711	-1.412406	-2.994239
H	5.532590	-2.591307	-1.856656
C	-1.442935	-1.448371	0.532050
O	-2.105059	-2.460183	0.628808
O	-0.700183	-0.958566	1.545066
C	-1.360144	-0.588534	-0.693727
C	-0.010508	-0.538420	-1.425717
H	-0.048552	-1.327285	-2.189082
H	0.046256	0.409153	-1.971040
C	-0.726997	-1.708000	2.774573
H	-0.055786	-1.181517	3.448983
H	-0.380830	-2.729140	2.608768
H	-1.739947	-1.729946	3.177989
H	-1.641961	0.675331	-0.251645
C	-4.493692	-0.700824	-0.033073
O	-4.472594	-0.271282	1.097336
O	-5.126584	-1.835084	-0.385580
C	-3.833838	-0.051733	-1.240521
C	-2.529513	-0.786367	-1.654626
H	-2.733200	-1.854603	-1.775808
H	-2.240950	-0.392713	-2.633373
C	-5.679275	-2.614997	0.691695
H	-6.173199	-3.458791	0.214762
H	-6.394242	-2.023309	1.264562
H	-4.877317	-2.959152	1.345501
H	-4.537273	-0.173632	-2.069086
C	-1.590510	2.927728	-0.412729
O	-1.392321	3.343671	-1.537052
O	-1.052850	3.487474	0.692536
C	-2.432377	1.755584	-0.077732
C	-3.568320	1.452252	-1.034314
H	-3.327669	1.891751	-2.005607
H	-4.485395	1.934771	-0.677359
C	-0.116149	4.553449	0.460084
H	0.165253	4.912504	1.448324
H	-0.580259	5.352688	-0.119626
H	0.755227	4.169956	-0.070872
H	-2.664563	1.659664	0.978685

Methyl acrylate pentamer backbiting TS with RSRR chirality
Standard orientation Energy= -1533.46826794

C	-2.345516	-1.875974	0.078704
O	-2.919229	-2.063115	1.126959
O	-2.504793	-2.649582	-1.009426
C	-1.369405	-0.743415	-0.197726
C	-2.102306	0.463335	-0.851454
H	-1.346863	1.229233	-1.046023
H	-2.501482	0.151606	-1.820207
C	-3.521675	-3.667600	-0.927555
H	-3.451124	-4.222094	-1.860669
H	-4.501379	-3.198364	-0.834214
H	-3.339452	-4.321967	-0.074494
H	-0.678592	-1.125476	-0.954137
C	-4.520772	0.318071	-0.069728
O	-4.870619	-0.420762	-0.961557
O	-5.272479	0.571809	1.017743
C	-3.231914	1.127007	-0.043902
C	-6.532231	-0.120078	1.097741
H	-6.998861	0.232873	2.014757
H	-6.366397	-1.197016	1.144414
H	-7.154592	0.114897	0.233172
H	-2.945553	1.239884	1.003967
C	-3.537482	2.534846	-0.602393
H	-2.650158	3.166735	-0.521621
H	-3.825872	2.480874	-1.655931
H	-4.347348	3.011039	-0.046109
C	1.359901	1.111602	1.743341
O	0.698718	2.091174	2.017784
O	2.618765	0.924244	2.188522
C	0.885651	0.005362	0.846570
C	-0.582212	-0.350295	1.075836
H	-0.614075	-1.184488	1.786637
H	-1.070261	0.485862	1.574296
C	3.186960	1.998132	2.962565
H	4.166000	1.641860	3.274884
H	2.560381	2.218692	3.827161
H	3.285160	2.891178	2.344116
C	3.870085	-2.062305	-0.521833
O	3.764482	-2.868589	-1.415567
O	4.770460	-2.179649	0.476627
C	3.008970	-0.825053	-0.342945
C	1.828409	-1.168414	0.604627
H	1.257915	-1.993557	0.165540
H	2.227494	-1.521559	1.560826
C	5.611492	-3.348599	0.437359
H	6.260703	-3.267668	1.306309
H	6.198968	-3.363703	-0.481473
H	5.007699	-4.255603	0.491261
H	3.618608	-0.055135	0.130098
C	2.122441	2.202043	-1.416315
O	3.240252	2.441118	-1.005710
O	1.225053	3.166139	-1.733554
C	1.546406	0.852196	-1.589982
C	2.496370	-0.322363	-1.709483
H	3.358223	-0.039573	-2.323184
H	1.997940	-1.147124	-2.223136
C	1.651905	4.522385	-1.514192
H	0.829597	5.145054	-1.859831
H	2.559515	4.734915	-2.080614
H	1.839411	4.693332	-0.452979
H	0.676063	0.839087	-2.240035
H	0.992992	0.608628	-0.352762

Methyl acrylate pentamer backbiting TS with SRRR chirality
Standard orientation Energy= -1533.47364283

C	2.013032	1.944348	0.669110
O	1.819512	2.837973	1.459747
O	2.905208	2.027250	-0.337711
C	1.378334	0.562919	0.738765
C	2.176231	-0.318707	1.738107

H	1.986834	0.054377	2.750494
H	1.763672	-1.330156	1.684872
H	1.439442	0.111937	-0.252429
C	4.091980	-0.962901	0.202614
O	3.439176	-1.745513	-0.449123
O	5.310446	-0.528730	-0.178589
C	3.699504	-0.392336	1.554396
C	5.805110	-1.044002	-1.428196
H	6.780621	-0.582830	-1.566664
H	5.132797	-0.772786	-2.243177
H	5.897599	-2.130272	-1.384769
H	4.138834	0.606012	1.621017
C	4.336766	-1.263544	2.658808
H	4.102888	-0.847254	3.641660
H	3.948908	-2.285121	2.621628
H	5.423138	-1.300460	2.554955
C	-1.019269	1.878658	-0.838842
O	0.033813	2.398839	-1.137489
O	-2.173827	2.156886	-1.497807
C	-1.216290	0.847439	0.220588
C	-0.084522	0.644746	1.226258
H	-0.143256	1.443464	1.977027
H	-0.315431	-0.283732	1.756862
C	-2.071712	3.116688	-2.565739
H	-3.076653	3.205459	-2.973231
H	-1.376172	2.766372	-3.329639
H	-1.727437	4.078116	-2.182539
H	-1.255908	-0.276555	-0.496043
C	-4.939812	-0.141223	0.869592
O	-5.071974	0.026848	2.057691
O	-5.963922	-0.468868	0.050628
C	-3.636454	-0.046551	0.096743
C	-2.605011	0.797239	0.870450
H	-2.993766	1.812365	0.994371
H	-2.509254	0.378156	1.874235
C	-7.248629	-0.638477	0.680271
H	-7.934403	-0.894656	-0.124264
H	-7.557722	0.286106	1.169420
H	-7.207990	-1.438779	1.420408
H	-3.859032	0.427745	-0.861636
C	-0.828534	-2.487755	-0.810681
O	-0.673861	-3.113866	0.219244
O	-0.072883	-2.665180	-1.915807
C	-1.833806	-1.422661	-1.012403
C	-3.089828	-1.470922	-0.176647
H	-2.875347	-1.978420	0.767961
H	-3.858342	-2.055385	-0.695710
C	0.981079	-3.647155	-1.817455
H	1.390243	-3.726613	-2.822483
H	0.574395	-4.604877	-1.490389
H	1.747450	-3.305651	-1.122956
H	-1.959384	-1.124052	-2.051217
C	3.552503	3.300966	-0.500344
H	4.233857	3.176996	-1.339491
H	2.810759	4.070128	-0.719197
H	4.101121	3.574639	0.402275

Methyl acrylate pentamer backbiting TS with RRSS chirality

Standard orientation Energy= -1533.47044609

C	1.150613	0.377082	-0.452120
O	0.676912	1.429782	-0.813853
O	1.822436	-0.447761	-1.280931
C	1.083606	-0.190518	0.956871
C	2.375074	0.139659	1.754851
H	2.538512	1.221434	1.768568
H	2.177034	-0.166405	2.785664
C	1.967930	0.004621	-2.641003
H	2.577126	-0.751200	-3.132922
H	2.460714	0.977061	-2.665131
H	0.989409	0.073144	-3.117141
H	1.014855	-1.276352	0.877843
C	4.356259	0.128653	0.149752
O	4.252681	1.296932	-0.139526
O	5.180713	-0.720541	-0.503770
C	3.666388	-0.568181	1.311640
C	5.974740	-0.148089	-1.557812
H	6.568043	-0.969397	-1.954225
H	6.621903	0.638150	-1.165999
H	5.335721	0.272509	-2.335387
H	3.442740	-1.589435	0.990958
C	4.674415	-0.643622	2.481567
H	4.917450	0.357000	2.850177
H	4.243855	-1.214950	3.307210
H	5.600318	-1.133480	2.174232
C	-1.620036	-1.698854	1.730958
O	-0.712667	-2.394223	2.140545
O	-2.857724	-2.183510	1.483820
C	-1.512062	-0.242991	1.400568
C	-0.143293	0.360372	1.712301
H	-0.187926	1.440413	1.573306
H	0.024357	0.193593	2.783515
C	-3.050046	-3.590981	1.708874
H	-4.073569	-3.795024	1.403138
H	-2.905690	-3.831613	2.763347
H	-2.348476	-4.172738	1.109729
H	-1.722179	-0.264483	0.057801
C	-2.352010	2.805268	0.556004
O	-1.807916	3.366948	1.477710
O	-2.279475	3.231566	-0.715696
C	-3.243254	1.572271	0.718488
C	-2.709515	0.622361	1.822197
H	-2.425344	1.225547	2.689537
H	-3.536171	-0.022749	2.118084
C	-1.423907	4.369430	-0.955724
H	-1.549031	4.605872	-2.010053
H	-0.389689	4.098757	-0.743035
H	-1.725119	5.210539	-0.331034
H	-4.188520	1.984394	1.092999
C	-2.526402	-1.150911	-1.879470
O	-3.550777	-1.798309	-1.935375
O	-1.384847	-1.524442	-2.521139
C	-2.323002	0.095750	-1.115238
C	-3.546676	0.796368	-0.577325
H	-4.320351	0.053395	-0.370266
H	-3.947006	1.480827	-1.331748
C	-1.459980	-2.750777	-3.266356
H	-0.468939	-2.895711	-3.691689
H	-2.208698	-2.674811	-4.056622
H	-1.718479	-3.584309	-2.611239
H	-1.544585	0.737021	-1.518034

Methyl acrylate pentamer backbiting TS with RSRS chirality

Standard orientation Energy= -1533.47197155

C	-1.178450	-0.144054	-1.434366
O	-0.459959	-0.441356	-2.362878
O	-2.042279	0.888737	-1.472449
C	-1.266625	-0.891180	-0.114931
C	-2.196664	-2.121073	-0.283224
H	-1.738350	-2.814964	-0.996128
H	-2.240876	-2.629608	0.683782
C	-2.066565	1.664422	-2.688068
H	-2.909682	2.343411	-2.580433

H	-2.201227	1.016366	-3.554476
H	-1.136513	2.224865	-2.785591
H	-1.704115	-0.228570	0.627580
C	-4.390832	-0.980365	0.253582
O	-4.186951	-0.942125	1.444029
O	-5.377208	-0.283159	-0.348952
C	-3.632403	-1.837984	-0.748236
C	-6.201558	0.522964	0.513456
H	-6.931053	0.995671	-0.140554
H	-5.597412	1.275646	1.021730
H	-6.699852	-0.099522	1.257984
H	-3.628987	-1.296734	-1.697768
C	-4.409484	-3.155538	-0.956840
H	-3.905653	-3.771281	-1.705895
H	-4.463153	-3.727416	-0.026418
H	-5.426739	-2.962850	-1.303551
C	0.766780	0.324292	2.222188
O	1.546425	0.974834	2.883600
O	-0.534114	0.175786	2.561272
C	1.120401	-0.376574	0.945014
C	0.121571	-1.374690	0.356175
H	0.601073	-1.883892	-0.484323
H	-0.043989	-2.141021	1.124132
C	-0.959574	0.843178	3.764196
H	-2.018635	0.613982	3.858964
H	-0.800135	1.918959	3.678980
H	-0.403606	0.467371	4.624184
H	1.276871	0.671013	0.090445
C	4.642575	-1.303812	-0.419737
O	5.688000	-0.703291	-0.451569
O	4.565926	-2.653279	-0.357584
C	3.256747	-0.682607	-0.437835
C	2.563231	-0.883325	0.934749
H	2.570625	-1.948832	1.189370
H	3.134215	-0.350948	1.697990
C	5.822373	-3.355929	-0.314465
H	5.563434	-4.411401	-0.266535
H	6.395103	-3.060023	0.565501
H	6.408003	-3.144341	-1.210086
H	2.684508	-1.221764	-1.201749
C	1.757074	2.819918	-0.509207
O	2.582561	3.522460	0.029459
O	0.512563	3.266121	-0.841753
C	1.927366	1.396753	-0.872487
C	3.316565	0.812836	-0.788710
H	3.902123	1.349340	-0.040073
H	3.836751	0.943680	-1.744653
C	0.230056	4.630727	-0.487669
H	-0.799184	4.805966	-0.794964
H	0.905917	5.310498	-1.009190
H	0.338916	4.778246	0.587860
H	1.336432	1.092585	-1.733047

Methyl acrylate pentamer backbiting TS with RSSR chirality
Standard orientation Energy= -1533.47300844

C	-2.324121	1.238960	1.652707
O	-2.499922	1.640724	2.777747
O	-3.002045	1.698800	0.582603
C	-1.383478	0.099045	1.282541
C	-1.958027	-1.218969	1.857845
H	-1.182525	-1.987701	1.810559
H	-2.203391	-1.065598	2.912894
C	-3.874836	2.816403	0.830088
H	-4.339206	3.041184	-0.127898
H	-3.292825	3.667562	1.185077
H	-4.630851	2.558207	1.573081
H	-1.353468	0.025918	0.194475
C	-2.813659	-2.226748	-0.275499
O	-1.727005	-2.650940	-0.598610
O	-3.856989	-2.158058	-1.124422
C	-3.194041	-1.751034	1.120209
C	-3.614683	-2.626309	-2.465724
H	-4.547117	-2.463782	-3.001823
H	-3.356646	-3.686320	-2.458004
H	-2.801104	-2.061072	-2.921487
H	-3.942221	-0.962425	1.001531
C	-3.845876	-2.922558	1.884018
H	-3.134857	-3.742256	2.020080
H	-4.170680	-2.586828	2.871617
H	-4.718138	-3.306726	1.351019
C	0.748145	2.430961	0.534610
O	-0.266119	3.017439	0.851317
O	1.720806	2.998149	-0.211890
C	1.042876	1.006258	0.866855
C	0.049030	0.350174	1.821452
H	0.469966	-0.613965	2.116595
H	-0.016380	0.949219	2.736926
C	1.491012	4.354719	-0.630817
H	2.357383	4.621967	-1.231504
H	1.404643	5.011634	0.235915
H	0.577654	4.421306	-1.223652
H	0.870635	0.406555	-0.319719
C	4.304735	-1.106014	0.815464
O	5.186918	-0.318919	1.057648
O	4.501890	-2.440375	0.738640
C	2.854297	-0.755012	0.534085
C	2.504991	0.633543	1.107327
H	2.704911	0.639838	2.185766
H	3.167367	1.372013	0.658832
C	5.855185	-2.893532	0.936903
H	5.815331	-3.976171	0.840129
H	6.210342	-2.608535	1.928012
H	6.515361	-2.463818	0.182341
H	2.242700	-1.531021	1.005957
C	0.864679	0.222279	-2.573632
O	1.681585	0.782923	-3.273641
O	-0.466252	0.220226	-2.850072
C	1.151218	-0.497462	-1.315702
C	2.594974	-0.802297	-0.995879
H	3.237776	-0.077705	-1.502176
H	2.859295	-1.797300	-1.370870
C	-0.859374	0.947039	-4.026007
H	-1.943607	0.866211	-4.070215
H	-0.405120	0.510209	-4.916999
H	-0.558326	1.993073	-3.950444
H	0.421891	-1.270091	-1.075625

Methyl acrylate molecule
Standard orientation Energy= -306.554623910

C	0.000000	0.132263	0.000000
O	0.509764	1.228560	0.000000
O	0.709311	-1.019842	0.000000
C	-1.456685	-0.153470	0.000000
C	-2.352077	0.832358	0.000000
H	-2.035333	1.869989	0.000000
H	-3.417352	0.630127	0.000000
C	2.138828	-0.878221	0.000000
H	2.531778	-1.892207	0.000000
H	2.468719	-0.338744	0.888497
H	2.468719	-0.338744	-0.888497
H	-1.749525	-1.197742	0.000000

Methyl acrylate dimer ECR

Standard orientation Energy= -613.734044849

C	2.309356	-0.128541	0.059528
O	2.695976	-1.047220	-0.644441
O	3.037995	1.002386	0.264383
C	1.047341	-0.091138	0.769453
C	0.084782	-1.219853	0.689607
H	0.606432	-2.114225	0.342289
H	-0.327750	-1.415078	1.685320
C	4.309032	1.054914	-0.400810
H	4.744089	2.012594	-0.122674
H	4.179741	0.992573	-1.482902
H	4.948790	0.233627	-0.072785
H	0.806779	0.796172	1.342063
C	-1.951493	0.208006	0.201743
O	-2.047225	0.588547	1.344499
O	-2.629796	0.765409	-0.822655
C	-1.103426	-0.961825	-0.272837
C	-3.515329	1.850191	-0.483355
H	-3.959387	2.166777	-1.424395
H	-2.956992	2.668412	-0.026968
H	-4.285463	1.510669	0.210618
H	-0.717745	-0.710229	-1.264360
C	-1.991746	-2.215281	-0.400238
H	-1.400746	-3.053866	-0.775499
H	-2.407977	-2.497958	0.570631
H	-2.816474	-2.041279	-1.094163

Methyl acrylate propagation TS to produce an RR chirality trimer

Standard orientation Energy= -920.286307097

C	0.853253	1.580326	0.557642
O	1.523739	1.316968	1.536118
O	1.177091	2.541896	-0.337245
C	-0.386314	0.887160	0.183477
C	-1.054896	0.053190	1.232048
H	-0.300219	-0.532746	1.762659
H	-1.479256	0.741028	1.978487
C	2.445649	3.191799	-0.129224
H	2.512548	3.949066	-0.907593
H	3.252018	2.464523	-0.228757
H	2.480748	3.652449	0.858913
H	-1.003492	1.396220	-0.549054
C	-3.294419	-0.091971	0.072442
O	-3.732461	0.966311	0.454718
O	-3.773367	-0.743696	-1.010242
C	-2.179442	-0.875748	0.747205
C	-4.884036	-0.119459	-1.685542
H	-5.127152	-0.779658	-2.514968
H	-4.601455	0.868896	-2.049953
H	-5.733925	-0.022067	-1.008909
H	-1.787862	-1.588339	0.018022
C	-2.779666	-1.671813	1.923708
H	-1.999708	-2.260958	2.412106
H	-3.214211	-0.997053	2.665317
H	-3.558281	-2.357448	1.581507
C	2.758139	-1.087749	-0.635202
O	3.328694	-0.088022	-1.021635
O	3.384306	-2.094190	0.019452
C	1.329731	-1.383197	-0.814966
C	0.493326	-0.480449	-1.408619
H	0.916266	0.378634	-1.914791
H	-0.506795	-0.779009	-1.695379
C	4.786941	-1.902493	0.268851
H	5.115360	-2.800367	0.787912
H	4.944077	-1.020244	0.891155
H	5.329506	-1.780913	-0.670014
H	0.968223	-2.323727	-0.416913

Methyl acrylate propagation TS to produce an RS chirality trimer

Standard orientation Energy= -920.287973352

C	-0.161134	1.932579	-0.384560
O	0.142768	1.940168	-1.561603
O	-0.401972	3.070664	0.322144
C	-0.288621	0.737168	0.452384
C	-0.316128	-0.605131	-0.207707
H	0.421001	-0.625647	-1.013188
H	-0.048896	-1.378176	0.515776
C	-0.288230	4.300183	-0.413248
H	-0.511613	5.089196	0.301875
H	-1.000707	4.320078	-1.239536
H	0.720597	4.418766	-0.812468
H	-0.831724	0.862473	1.381447
C	-2.775866	-1.037309	0.232380
O	-2.598573	-1.212142	1.414954
O	-4.003183	-0.915112	-0.317899
C	-1.689173	-0.956133	-0.828267
C	-5.118674	-1.031138	0.585668
H	-6.005805	-0.905596	-0.031138
H	-5.070780	-0.256355	1.351981
H	-5.120152	-2.010341	1.066505
H	-1.979735	-0.175621	-1.537087
C	-1.619033	-2.294847	-1.591484
H	-0.870464	-2.230152	-2.384414
H	-1.335076	-3.110834	-0.921285
H	-2.580815	-2.536649	-2.047623
C	3.228397	-0.734088	0.478537
O	2.791319	-1.761331	0.955780
O	4.287530	-0.701597	-0.366884
C	2.712348	0.618795	0.728274
C	1.667248	0.829507	1.582726
H	1.331362	0.012738	2.210803
H	1.419342	1.834731	1.897815
C	4.886113	-1.971783	-0.672305
H	5.709612	-1.750340	-1.347760
H	4.161470	-2.630305	-1.153960
H	5.252792	-2.450696	0.237037
H	3.173010	1.430709	0.178906

Methyl acrylate MCR pentamer with the radical on unit 3

Standard orientation Energy= -1533.50983982

C	-1.914100	-0.829294	-1.390733
O	-1.261481	-1.728064	-1.869235
O	-2.770669	-0.075725	-2.111641
C	-1.889363	-0.414875	0.071631
C	-2.474526	-1.539024	0.952018
H	-1.863869	-2.438093	0.824136
H	-2.389263	-1.228020	1.996904
C	-2.874367	-0.395947	-3.513427
H	-3.607546	0.299279	-3.915827
H	-3.206942	-1.426260	-3.645847
H	-1.909942	-0.264046	-4.005355
H	-2.486345	0.489204	0.186782
C	-4.875515	-0.746305	0.969027
O	-4.705593	0.087513	1.826622
O	-5.970995	-0.782263	0.180452
C	-3.938048	-1.905066	0.664569
C	-6.959949	0.236158	0.422970
H	-7.755210	0.045294	-0.294253
H	-6.531532	1.226965	0.266492
H	-7.338074	0.166294	1.443878

H	-4.065883	-2.159116	-0.391630
C	-4.377103	-3.125477	1.501191
H	-3.744876	-3.984931	1.265904
H	-4.284952	-2.916067	2.570311
H	-5.413466	-3.397546	1.289947
C	-0.092841	2.370921	-0.122569
O	0.437831	3.294687	-0.718777
O	-1.091260	2.564169	0.783094
C	0.254220	0.966106	-0.280416
C	-0.430520	-0.104050	0.516504
H	0.151057	-1.027751	0.455041
H	-0.481351	0.193668	1.568282
C	-1.499669	3.924798	0.987710
H	-2.305518	3.879699	1.717451
H	-1.851953	4.364740	0.053116
H	-0.670054	4.522787	1.369216
C	3.344984	1.565248	-0.040424
O	4.169362	2.222421	-0.630055
O	2.847932	1.911081	1.166310
C	2.719798	0.275960	-0.548286
C	1.347340	0.604275	-1.232766
H	1.498094	1.406760	-1.957755
H	1.049900	-0.300949	-1.772513
C	3.283219	3.183601	1.685061
H	2.815293	3.273435	2.663144
H	2.954895	3.986693	1.024215
H	4.369783	3.207407	1.775194
H	2.528031	-0.372878	0.308693
C	4.870858	-1.998250	0.078698
O	3.860236	-2.587516	0.381708
O	6.073227	-2.264173	0.634262
C	4.986040	-0.899854	-0.956673
C	3.645625	-0.445033	-1.540137
H	3.110712	-1.315841	-1.927705
H	3.847972	0.220518	-2.383308
C	6.098650	-3.313213	1.619978
H	7.134752	-3.381117	1.944219
H	5.770495	-4.257023	1.182244
H	5.449176	-3.063544	2.460172
H	5.540413	-0.064197	-0.521512
H	5.629094	-1.286485	-1.755107

Methyl acrylate MCR pentamer with the radical on unit 3 with reduced flexibility set to 10.4 angstrom
Standard orientation Energy= -1533.50942346

C	-1.958346	-1.159176	-1.234742
O	-1.331622	-2.144092	-1.550086
O	-2.764374	-0.493533	-2.088582
C	-1.954377	-0.527093	0.147909
C	-2.645821	-1.462461	1.164087
H	-2.103331	-2.412353	1.198968
H	-2.567672	-1.000677	2.152198
C	-2.847920	-1.022335	-3.427112
H	-3.538977	-0.368573	-3.954219
H	-3.223218	-2.046339	-3.410012
H	-1.866751	-1.007779	-3.903122
H	-2.498893	0.415163	0.101501
C	-4.972443	-0.495843	0.935617
O	-4.768519	0.451192	1.657589
O	-6.032428	-0.575349	0.103166
C	-4.126420	-1.758999	0.874461
C	-6.939595	0.542961	0.123772
H	-7.717336	0.299239	-0.596635
H	-6.421427	1.458046	-0.165766
H	-7.365072	0.671661	1.120025
H	-4.239241	-2.178303	-0.129389

C	-4.703478	-2.778440	1.881161
H	-4.139799	-3.713092	1.830067
H	-4.637037	-2.394677	2.902701
H	-5.750027	-2.999803	1.661436
C	-0.118138	2.178739	-0.246335
O	0.411348	3.042920	-0.926696
O	-1.109591	2.456251	0.644162
C	0.223190	0.762749	-0.281659
C	-0.491612	-0.226793	0.591319
H	0.065469	-1.167087	0.612939
H	-0.546543	0.159181	1.613789
C	-1.517782	3.829927	0.725433
H	-2.326703	3.849611	1.452786
H	-1.865709	4.185220	-0.246019
H	-0.689097	4.458909	1.055766
C	3.262756	1.551693	-0.159614
O	4.007756	2.222533	-0.833085
O	2.788364	1.950457	1.039980
C	2.732236	0.177730	-0.538091
C	1.317370	0.324869	-1.202309
H	1.403531	1.025763	-2.036005
H	1.062723	-0.661812	-1.601917
C	3.142195	3.289055	1.440821
H	2.698247	3.423932	2.424951
H	2.736015	4.009397	0.729834
H	4.226200	3.398273	1.490452
H	2.623179	-0.411505	0.374856
C	5.157533	-1.787783	0.169073
O	4.235062	-2.448961	0.584137
O	6.407026	-1.865892	0.676938
C	5.098912	-0.793378	-0.970627
C	3.686517	-0.539632	-1.508103
H	3.231443	-1.495002	-1.782052
H	3.773518	0.057421	-2.419660
C	6.596610	-2.802331	1.753895
H	7.647686	-2.725269	2.023288
H	6.357257	-3.815055	1.426473
H	5.962759	-2.541619	2.602616
H	5.582201	0.134312	-0.654396
H	5.734109	-1.192156	-1.769379

Methyl acrylate MCR pentamer with the radical on unit 3 with reduced flexibility set to 10.6 angstrom
Standard orientation Energy= -1533.50892818

C	-1.985031	-1.304278	-1.139665
O	-1.392688	-2.333701	-1.366680
O	-2.739716	-0.669128	-2.061094
C	-1.988326	-0.574649	0.194180
C	-2.730037	-1.411675	1.260274
H	-2.224482	-2.375782	1.372985
H	-2.652332	-0.883616	2.214631
C	-2.811063	-1.288490	-3.360930
H	-3.457815	-0.645280	-3.953211
H	-3.232270	-2.291592	-3.282782
H	-1.817883	-1.350300	-3.807318
H	-2.503928	0.376535	0.069279
C	-5.012004	-0.377417	0.904671
O	-4.789816	0.611742	1.561910
O	-6.051672	-0.477607	0.049141
C	-4.216330	-1.673205	0.960899
C	-6.913511	0.672856	-0.038395
H	-7.681287	0.406133	-0.761335
H	-6.351685	1.543519	-0.379004
H	-7.359378	0.891236	0.933005
H	-4.324975	-2.164673	-0.010156
C	-4.858872	-2.587253	2.028575

H	-4.333690	-3.544759	2.065099
H	-4.800466	-2.127236	3.018591
H	-5.908197	-2.783101	1.798292
C	-0.126789	2.082275	-0.294676
O	0.401546	2.911572	-1.017785
O	-1.113691	2.404045	0.585387
C	0.210999	0.664543	-0.265207
C	-0.523676	-0.281147	0.640415
H	0.018519	-1.228030	0.705249
H	-0.581389	0.147379	1.645666
C	-1.522162	3.780015	0.600693
H	-2.333212	3.833545	1.323899
H	-1.867214	4.089044	-0.387415
H	-0.694329	4.423852	0.903466
C	3.227955	1.532987	-0.215785
O	3.936478	2.198396	-0.932408
O	2.766880	1.965697	0.977265
C	2.739319	0.125940	-0.523450
C	1.306930	0.187371	-1.165541
H	1.362517	0.829505	-2.048222
H	1.073834	-0.831095	-1.491120
C	3.087170	3.329833	1.314989
H	2.654486	3.494025	2.299712
H	2.649749	4.006842	0.580391
H	4.168188	3.471975	1.342410
H	2.670823	-0.425927	0.416430
C	5.276836	-1.691891	0.205295
O	4.398260	-2.372440	0.680048
O	6.542178	-1.687061	0.678736
C	5.139725	-0.760735	-0.980060
C	3.700861	-0.596904	-1.483808
H	3.282801	-1.584105	-1.697489
H	3.735003	-0.043034	-2.425677
C	6.805207	-2.557860	1.794628
H	7.858521	-2.419351	2.028345
H	6.604812	-3.595798	1.525115
H	6.183411	-2.283467	2.647940
H	5.589756	0.202191	-0.727040
H	5.766837	-1.172636	-1.778520

Methyl acrylate MCR pentamer with the radical on unit 3 with reduced flexibility set to 10.8 angstrom
Standard orientation Energy= -1533.50816906

C	-2.013589	-1.461524	-1.015905
O	-1.514168	-2.557998	-1.117404
O	-2.629650	-0.833705	-2.039692
C	-2.031044	-0.625363	0.253826
C	-2.826443	-1.347223	1.364116
H	-2.362465	-2.317568	1.565550
H	-2.751677	-0.747337	2.275249
C	-2.665541	-1.545583	-3.292763
H	-3.192577	-0.890828	-3.982931
H	-3.195924	-2.491782	-3.178218
H	-1.653130	-1.742530	-3.647356
H	-2.512683	0.326962	0.036890
C	-5.052655	-0.257895	0.851905
O	-4.824999	0.766913	1.450093
O	-6.048741	-0.380029	-0.051455
C	-4.315123	-1.575057	1.043291
C	-6.857474	0.792231	-0.265748
H	-7.597073	0.502785	-1.009018
H	-6.243934	1.614872	-0.635266
H	-7.343567	1.095981	0.662443
H	-4.416026	-2.144364	0.114830
C	-5.030990	-2.365571	2.163094
H	-4.549697	-3.336885	2.300474

H	-4.984339	-1.822903	3.110890
H	-6.079870	-2.537751	1.912606
C	-0.143321	1.979945	-0.328398
O	0.384072	2.769671	-1.094888
O	-1.127778	2.347591	0.535592
C	0.192638	0.564211	-0.227622
C	-0.565489	-0.333381	0.708230
H	-0.038172	-1.283721	0.824611
H	-0.631173	0.141411	1.691966
C	-1.539189	3.721679	0.478145
H	-2.355731	3.809511	1.191679
H	-1.877389	3.979298	-0.526857
H	-0.714761	4.381804	0.754045
C	3.186334	1.511334	-0.268415
O	3.855331	2.164335	-1.032618
O	2.742490	1.983555	0.916156
C	2.741036	0.074767	-0.496251
C	1.291317	0.046351	-1.103819
H	1.312664	0.619594	-2.034659
H	1.083664	-1.000176	-1.346817
C	3.028715	3.370656	1.183187
H	2.611199	3.568523	2.168290
H	2.557131	3.999164	0.427000
H	4.105425	3.544807	1.181035
H	2.717714	-0.432627	0.470525
C	5.386495	-1.591098	0.236400
O	4.555335	-2.282410	0.776567
O	6.665170	-1.506409	0.664049
C	5.168379	-0.731827	-0.990278
C	3.705920	-0.656760	-1.448683
H	3.326464	-1.671295	-1.596664
H	3.684590	-0.153042	-2.418705
C	7.003534	-2.303897	1.813963
H	8.056426	-2.107029	2.003521
H	6.842118	-3.362556	1.605870
H	6.397695	-2.011780	2.672837
H	5.583734	0.261507	-0.805540
H	5.783827	-1.160123	-1.789135

Methyl acrylate MCR pentamer with the radical on unit 3 with reduced flexibility set to 11.0 angstrom
Standard orientation Energy= -1533.50708962

C	-2.048851	-1.628971	-0.860588
O	-1.715221	-2.789862	-0.807990
O	-2.426080	-1.020284	-2.004826
C	-2.081589	-0.676119	0.323534
C	-2.932632	-1.259210	1.472275
H	-2.516514	-2.224044	1.776818
H	-2.859528	-0.579099	2.325309
C	-2.406608	-1.834353	-3.194336
H	-2.730707	-1.178796	-3.999338
H	-3.087672	-2.679621	-3.087825
H	-1.399383	-2.207182	-3.384352
H	-2.522460	0.265612	-0.000212
C	-5.089502	-0.136530	0.771460
O	-4.868795	0.924723	1.305490
O	-6.015569	-0.296264	-0.198472
C	-4.420953	-1.457462	1.123017
C	-6.759141	0.881364	-0.565573
H	-7.449545	0.558493	-1.341653
H	-6.087473	1.651893	-0.946185
H	-7.303482	1.273025	0.294864
H	-4.514016	-2.115021	0.254180
C	-5.215129	-2.096368	2.287668
H	-4.785390	-3.067909	2.543330
H	-5.179290	-1.460009	3.175631

H	-6.260562	-2.248825	2.010967
C	-0.169880	1.875271	-0.356755
O	0.356968	2.621543	-1.165578
O	-1.157021	2.287677	0.482905
C	0.168827	0.467108	-0.177242
C	-0.614527	-0.379166	0.786402
H	-0.100365	-1.327757	0.961823
H	-0.691107	0.145028	1.743958
C	-1.576793	3.653704	0.345334
H	-2.403279	3.773910	1.042416
H	-1.903305	3.853546	-0.676457
H	-0.760190	4.333608	0.595368
C	3.136269	1.492119	-0.307309
O	3.769081	2.129874	-1.113815
O	2.702452	2.003264	0.864780
C	2.739066	0.031120	-0.457321
C	1.275111	-0.089646	-1.021959
H	1.261428	0.405344	-1.997340
H	1.098184	-1.158342	-1.176486
C	2.951494	3.408807	1.063427
H	2.543299	3.639822	2.045229
H	2.450623	3.987702	0.286789
H	4.022496	3.613491	1.035755
H	2.762989	-0.430042	0.532354
C	5.486399	-1.484553	0.260026
O	4.703490	-2.178900	0.864708
O	6.775386	-1.330357	0.633373
C	5.189162	-0.696517	-0.997322
C	3.708013	-0.705451	-1.404219
H	3.369812	-1.741585	-1.490513
H	3.632804	-0.251918	-2.396105
C	7.187153	-2.055682	1.806953
H	8.237856	-1.810473	1.945342
H	7.059586	-3.128751	1.657455
H	6.602709	-1.743260	2.673466
H	5.566807	0.321190	-0.876217
H	5.792815	-1.138137	-1.797811

Methyl acrylate MCR pentamer with the radical on unit 3 with reduced flexibility set to 11.2 angstrom
Standard orientation Energy= -1533.50556658

C	-2.094210	-1.812106	-0.684687
O	-1.991027	-2.998056	-0.474300
O	-2.158307	-1.285449	-1.926389
C	-2.132981	-0.731905	0.383604
C	-3.040427	-1.145248	1.561900
H	-2.685153	-2.091157	1.980381
H	-2.955745	-0.380742	2.338955
C	-2.085011	-2.222378	-3.019257
H	-2.143467	-1.619184	-3.922375
H	-2.916511	-2.926686	-2.971976
H	-1.145330	-2.775276	-2.986059
H	-2.526282	0.180790	-0.062295
C	-5.112964	0.002680	0.674554
O	-4.876885	1.098888	1.125276
O	-5.983362	-0.206486	-0.337260
C	-4.528736	-1.306310	1.184971
C	-6.650170	0.961577	-0.852571
H	-7.307250	0.597333	-1.639184
H	-5.923464	1.667604	-1.256563
H	-7.226217	1.450616	-0.065781
H	-4.627103	-2.046238	0.386343
C	-5.399299	-1.778146	2.377152
H	-5.033704	-2.738661	2.748087
H	-5.360526	-1.054698	3.195489
H	-6.440550	-1.904609	2.072909
C	-0.203328	1.758686	-0.411795
O	0.322477	2.456507	-1.263020
O	-1.201226	2.212898	0.392041
C	0.146940	0.367065	-0.141050
C	-0.662095	-0.424749	0.849071
H	-0.158522	-1.365273	1.088003
H	-0.748279	0.150706	1.776058
C	-1.640098	3.560578	0.161938
H	-2.486279	3.706699	0.829683
H	-1.943080	3.692755	-0.877833
H	-0.841184	4.268511	0.390687
C	3.080610	1.480066	-0.327692
O	3.690734	2.107796	-1.158946
O	2.628244	2.021654	0.823855
C	2.744366	-0.001501	-0.413542
C	1.274546	-0.222013	-0.937498
H	1.232054	0.184446	-1.952620
H	1.137104	-1.305612	-0.998722
C	2.829420	3.441399	0.966920
H	2.404819	3.698561	1.935181
H	2.318061	3.971898	0.163066
H	3.893498	3.679778	0.939762
H	2.812569	-0.419594	0.593196
C	5.587633	-1.361861	0.284100
O	4.852217	-2.061671	0.940181
O	6.879338	-1.140489	0.610902
C	5.220521	-0.639021	-0.993577
C	3.727961	-0.730120	-1.354687
H	3.437239	-1.783569	-1.392117
H	3.606143	-0.320941	-2.361145
C	7.355083	-1.798839	1.799709
H	8.398206	-1.505668	1.895547
H	7.268143	-2.881506	1.697882
H	6.783612	-1.474378	2.670430
H	5.554293	0.398469	-0.924145
H	5.818697	-1.086226	-1.794930

Methyl acrylate MCR pentamer with the radical on unit 3 with reduced flexibility set to 11.4 angstrom
Standard orientation Energy= -1533.50340819

C	-2.150755	-1.966650	-0.477444
O	-2.196829	-3.123736	-0.130697
O	-2.028730	-1.587522	-1.768039
C	-2.179814	-0.766552	0.454052
C	-3.145730	-0.998103	1.638527
H	-2.854322	-1.905189	2.175258
H	-3.047189	-0.153272	2.325444
C	-1.939919	-2.650322	-2.737461
H	-1.841244	-2.155953	-3.701209
H	-2.840542	-3.264995	-2.710573
H	-1.071548	-3.279216	-2.536619
H	-2.521422	0.098776	-0.112563
C	-5.125625	0.141066	0.550996
O	-4.856762	1.267995	0.895565
O	-5.958739	-0.135850	-0.476052
C	-4.629671	-1.130619	1.223558
C	-6.545739	0.998753	-1.141387
H	-7.183416	0.581983	-1.917821
H	-5.769280	1.627121	-1.579635
H	-7.132737	1.591744	-0.438648
H	-4.734662	-1.947316	0.504967
C	-5.578032	-1.429788	2.417028
H	-5.280653	-2.361554	2.903956
H	-5.538132	-0.625232	3.155632
H	-6.608871	-1.538082	2.073341
C	-0.220787	1.640715	-0.460957

O	0.305597	2.280298	-1.356116
O	-1.228323	2.141316	0.301401
C	0.137055	0.272807	-0.091137
C	-0.704322	-0.457597	0.922679
H	-0.218308	-1.389760	1.222652
H	-0.793777	0.167935	1.816648
C	-1.686014	3.461514	-0.030483
H	-2.557222	3.631089	0.598229
H	-1.955873	3.519297	-1.085910
H	-0.908874	4.199633	0.177385
C	3.036795	1.465140	-0.327169
O	3.634227	2.088992	-1.170337
O	2.551532	2.026368	0.801994
C	2.764047	-0.031407	-0.362472
C	1.289124	-0.350194	-0.830187
H	1.219868	-0.045940	-1.879610
H	1.190892	-1.438529	-0.787831
C	2.705234	3.455289	0.903942
H	2.251865	3.729096	1.854446
H	2.196333	3.944819	0.072971
H	3.761822	3.726278	0.891617
H	2.884061	-0.412126	0.654367
C	5.705746	-1.240634	0.269922
O	5.021142	-1.932281	0.987048
O	7.004505	-0.972901	0.526689
C	5.259679	-0.578480	-1.015072
C	3.755655	-0.743231	-1.312689
H	3.512189	-1.809402	-1.309992
H	3.580901	-0.371907	-2.326073
C	7.552080	-1.568623	1.717539
H	8.589956	-1.244845	1.752622
H	7.490698	-2.656490	1.663770
H	7.012192	-1.223079	2.600231
H	5.550431	0.473788	-0.993180
H	5.841880	-1.028598	-1.826218

Methyl acrylate MCR pentamer with the radical on unit 3 with reduced flexibility set to 11.6 angstrom
Standard orientation Energy= -1533.50023646

C	-2.211451	-2.066106	-0.293259
O	-2.379198	-3.177493	0.151514
O	-1.941824	-1.826096	-1.595155
C	-2.229038	-0.779488	0.514254
C	-3.245197	-0.861253	1.682390
H	-3.006542	-1.721347	2.313958
H	-3.137065	0.042810	2.287438
C	-1.846165	-2.983388	-2.448648
H	-1.620960	-2.595061	-3.439355
H	-2.790268	-3.529579	-2.455126
H	-1.051483	-3.647129	-2.105287
H	-2.524731	0.033808	-0.147242
C	-5.147299	0.239501	0.427015
O	-4.843698	1.382761	0.676323
O	-5.961599	-0.098509	-0.597131
C	-4.722612	-0.983493	1.226686
C	-6.485519	0.991620	-1.379095
H	-7.117910	0.529659	-2.133993
H	-5.673670	1.548822	-1.848416
H	-7.067580	1.667141	-0.750650
H	-4.826624	-1.855133	0.575995
C	-5.743482	-1.152457	2.393915
H	-5.503763	-2.049304	2.969845
H	-5.713494	-0.290486	3.065033
H	-6.756729	-1.257426	2.001284
C	-0.220575	1.529788	-0.518448
O	0.324503	2.103941	-1.446012

O	-1.248367	2.078662	0.180245
C	0.135166	0.194197	-0.040670
C	-0.748702	-0.470031	0.988560
H	-0.284912	-1.391459	1.350391
H	-0.840982	0.204284	1.846663
C	-1.711176	3.364470	-0.262505
H	-2.612273	3.560159	0.314228
H	-1.933969	3.344284	-1.330116
H	-0.955008	4.128938	-0.072960
C	2.998173	1.465612	-0.244632
O	3.609068	2.122812	-1.051900
O	2.438735	1.996800	0.865811
C	2.799310	-0.041832	-0.294509
C	1.323422	-0.457486	-0.702960
H	1.238411	-0.265987	-1.778092
H	1.266237	-1.538621	-0.551930
C	2.540348	3.427954	0.992026
H	2.019580	3.676305	1.914557
H	2.071085	3.913672	0.135790
H	3.586634	3.731512	1.049664
H	2.980136	-0.420397	0.714451
C	5.835569	-1.132426	0.207143
O	5.197741	-1.827604	0.963208
O	7.144479	-0.853756	0.390758
C	5.312727	-0.474480	-1.050004
C	3.798367	-0.692009	-1.287025
H	3.599608	-1.767624	-1.297524
H	3.571809	-0.312145	-2.287009
C	7.761398	-1.438698	1.552563
H	8.796929	-1.106441	1.529114
H	7.706230	-2.527309	1.507626
H	7.267957	-1.092819	2.461930
H	5.562649	0.588090	-1.025715
H	5.872404	-0.893308	-1.892632

Methyl acrylate MCR pentamer with the radical on unit 3 with reduced flexibility set to 11.8 angstrom
Standard orientation Energy= -1533.49560303

C	2.272733	-2.082426	0.184613
O	2.486204	-3.167580	-0.303477
O	1.947141	-1.910647	1.484726
C	2.286307	-0.759123	-0.560903
C	3.338894	-0.771409	-1.709274
H	3.128041	-1.603337	-2.386634
H	3.232780	0.159153	-2.273075
C	1.847296	-3.108894	2.279018
H	1.577494	-2.772989	3.277737
H	2.802766	-3.634742	2.295225
H	1.080878	-3.772193	1.875751
H	2.553082	0.026882	0.144054
C	5.195621	0.292221	-0.354280
O	4.868336	1.439555	-0.549006
O	6.003936	-0.078710	0.663647
C	4.812414	-0.897983	-1.221401
C	6.491624	0.981858	1.507028
H	7.124414	0.496712	2.246925
H	5.660816	1.496105	1.991978
H	7.066199	1.701519	0.922164
H	4.904666	-1.795698	-0.605323
C	5.896255	-1.009793	-2.351163
H	5.693607	-1.883828	-2.974201
H	5.891206	-0.120288	-2.985782
H	6.886780	-1.123388	-1.907471
C	0.184091	1.413562	0.602872
O	-0.409509	1.901360	1.549951
O	1.237781	2.027853	0.004660

C	-0.141882	0.124261	-0.005922
C	0.801591	-0.452496	-1.044472
H	0.372511	-1.358300	-1.480871
H	0.896301	0.280005	-1.854178
C	1.663872	3.273556	0.579689
H	2.577163	3.536790	0.050929
H	1.856576	3.156145	1.646998
H	0.899484	4.040638	0.440973
C	-2.969738	1.462556	0.082828
O	-3.616900	2.178817	0.807318
O	-2.303401	1.924785	-1.000703
C	-2.848994	-0.048679	0.199248
C	-1.375372	-0.562726	0.543027
H	-1.293026	-0.514333	1.634738
H	-1.352849	-1.617445	0.258612
C	-2.356006	3.348806	-1.206297
H	-1.740038	3.539740	-2.082624
H	-1.963511	3.871090	-0.333035
H	-3.383610	3.669783	-1.383524
H	-3.101819	-0.448422	-0.786359
C	-5.972870	-1.043279	-0.125448
O	-5.378099	-1.746933	-0.908592
O	-7.294807	-0.784164	-0.226798
C	-5.375561	-0.348759	1.076549
C	-3.849448	-0.602492	1.255955
H	-3.688339	-1.682916	1.319168
H	-3.570359	-0.180608	2.225532
C	-7.979048	-1.396668	-1.335340
H	-9.015067	-1.076456	-1.249196
H	-7.906865	-2.483601	-1.276021
H	-7.551201	-1.060953	-2.281041
H	-5.591938	0.719487	1.012384
H	-5.902252	-0.711790	1.964391

Methyl acrylate MCR pentamer with the radical on unit 3 with reduced flexibility set to 12.0 angstrom
Standard orientation Energy= -1533.48889095

C	2.350305	-2.064860	0.138034
O	2.591616	-3.140648	-0.358018
O	2.002023	-1.913782	1.435064
C	2.349028	-0.733618	-0.592623
C	3.428403	-0.717970	-1.730142
H	3.236938	-1.543246	-2.421019
H	3.319136	0.218656	-2.283030
C	1.911029	-3.122580	2.214104
H	1.620358	-2.802929	3.212320
H	2.875063	-3.632234	2.239028
H	1.162297	-3.794164	1.791748
H	2.592772	0.049902	0.122903
C	5.253827	0.344931	-0.328021
O	4.900145	1.487921	-0.500730
O	6.065047	-0.027187	0.687759
C	4.905322	-0.836073	-1.221610
C	6.522345	1.027445	1.554989
H	7.161834	0.542740	2.289435
H	5.676758	1.512456	2.044523
H	7.083438	1.771984	0.988411
H	4.990835	-1.740641	-0.614687
C	6.048345	-0.927351	-2.313844
H	5.878901	-1.794824	-2.955284
H	6.067162	-0.029145	-2.935354
H	7.013535	-1.041540	-1.818805
C	0.132897	1.293363	0.672167
O	-0.510737	1.702773	1.623811
O	1.202587	1.964024	0.170728
C	-0.151683	0.050673	-0.044644

C	0.851793	-0.449306	-1.080593
H	0.462987	-1.346277	-1.569803
H	0.935670	0.324007	-1.853669
C	1.575406	3.172197	0.853054
H	2.489023	3.506739	0.366690
H	1.751920	2.974679	1.911457
H	0.788574	3.923219	0.760861
C	-2.965523	1.436330	-0.043087
O	-3.635866	2.188905	0.621213
O	-2.225102	1.853587	-1.097365
C	-2.900348	-0.073502	0.112946
C	-1.425661	-0.661768	0.393005
H	-1.354680	-0.749869	1.483516
H	-1.423499	-1.675159	-0.014260
C	-2.243650	3.269734	-1.354499
H	-1.565653	3.423268	-2.191454
H	-1.905999	3.819799	-0.475365
H	-3.252037	3.595889	-1.613946
H	-3.210213	-0.477244	-0.854945
C	-6.097851	-0.970223	-0.081155
O	-5.540195	-1.654594	-0.907631
O	-7.427407	-0.731192	-0.100702
C	-5.440724	-0.277133	1.088875
C	-3.901454	-0.563997	1.211758
H	-3.770638	-1.647066	1.298605
H	-3.578575	-0.128384	2.161570
C	-8.165956	-1.336409	-1.177882
H	-9.200008	-1.034942	-1.025687
H	-8.073422	-2.422825	-1.142103
H	-7.799775	-0.977831	-2.140937
H	-5.632074	0.795412	1.017083
H	-5.932630	-0.618189	2.004183

Methyl acrylate MCR pentamer with the radical on unit 3 with reduced flexibility set to 12.2 angstrom
Standard orientation Energy= -1533.47971946

C	2.431532	-2.045506	0.145052
O	2.704304	-3.124115	-0.328264
O	2.064780	-1.879877	1.435431
C	2.405718	-0.728325	-0.609324
C	3.505522	-0.713732	-1.744584
H	3.329760	-1.551281	-2.424408
H	3.386742	0.213383	-2.310991
C	1.992240	-3.076550	2.234678
H	1.681309	-2.746405	3.223342
H	2.967551	-3.562778	2.280722
H	1.264953	-3.773029	1.815148
H	2.629986	0.073216	0.091954
C	5.308087	0.387840	-0.336758
O	4.923523	1.520132	-0.514271
O	6.128674	0.043064	0.681711
C	4.992551	-0.806175	-1.224599
C	6.556380	1.114082	1.543721
H	7.207959	0.650703	2.281332
H	5.697558	1.578706	2.030001
H	7.097800	1.870646	0.973900
H	5.078840	-1.702663	-0.606106
C	6.191035	-0.898874	-2.282874
H	6.054032	-1.774936	-2.919794
H	6.227608	-0.006048	-2.910634
H	7.130014	-0.999701	-1.738364
C	0.099423	1.206817	0.689540
O	-0.569998	1.580151	1.638110
O	1.174235	1.902648	0.235924
C	-0.160150	-0.013756	-0.074011
C	0.887856	-0.484852	-1.096556

H	0.531208	-1.391602	-1.592201
H	0.951701	0.292701	-1.867916
C	1.516733	3.092716	0.964560
H	2.431102	3.459132	0.503049
H	1.681609	2.861826	2.018120
H	0.718766	3.833440	0.886616
C	-2.987885	1.408088	-0.077860
O	-3.665570	2.163608	0.575816
O	-2.217832	1.824965	-1.111048
C	-2.948227	-0.102760	0.066278
C	-1.465069	-0.728573	0.303261
H	-1.399774	-0.893997	1.385434
H	-1.476410	-1.711963	-0.171210
C	-2.219281	3.242197	-1.361538
H	-1.518147	3.394884	-2.179385
H	-1.901201	3.786112	-0.471337
H	-3.218021	3.577055	-1.646132
H	-3.279787	-0.488761	-0.901656
C	-6.205013	-0.923780	-0.085631
O	-5.666680	-1.554267	-0.965945
O	-7.538728	-0.711782	-0.043263
C	-5.516803	-0.273224	1.089823
C	-3.962960	-0.584696	1.170971
H	-3.853410	-1.671383	1.241469
H	-3.619953	-0.170406	2.123229
C	-8.305906	-1.273557	-1.123980
H	-9.338826	-1.001235	-0.918485
H	-8.192585	-2.358274	-1.149997
H	-7.982217	-0.857109	-2.078930
H	-5.691385	0.803832	1.049691
H	-5.985127	-0.634561	2.008926

Methyl acrylate MCR pentamer with the radical on unit 3 with reduced flexibility set to 12.4 angstrom

Standard orientation Energy= -1533.46799390

C	2.506453	-2.020804	0.178317
O	2.801529	-3.109290	-0.257789
O	2.133874	-1.819431	1.462165
C	2.455983	-0.730974	-0.619446
C	3.572713	-0.736574	-1.757063
H	3.407375	-1.594872	-2.412732
H	3.444787	0.172832	-2.349115
C	2.081772	-2.990047	2.300436
H	1.761106	-2.633432	3.276732
H	3.066094	-3.455574	2.365322
H	1.369482	-3.714077	1.902330
H	2.664866	0.098120	0.053406
C	5.362538	0.415886	-0.366576
O	4.952151	1.536058	-0.562704
O	6.191530	0.108139	0.657536
C	5.073225	-0.799773	-1.232545
C	6.594587	1.203826	1.500000
H	7.256475	0.768755	2.245664
H	5.725397	1.657479	1.978166
H	7.118727	1.962313	0.916699
H	5.162480	-1.681579	-0.594004
C	6.322263	-0.904755	-2.262100
H	6.212487	-1.794990	-2.883751
H	6.373657	-0.023103	-2.903643
H	7.235312	-0.987206	-1.673428
C	0.076793	1.141469	0.691603
O	-0.605864	1.496548	1.637528
O	1.152342	1.850927	0.261835
C	-0.168072	-0.069077	-0.093556
C	0.914754	-0.532486	-1.102211
H	0.584320	-1.456267	-1.584244
H	0.957199	0.233786	-1.886511
C	1.476655	3.032309	1.012764
H	2.386876	3.419806	0.560234
H	1.642240	2.784562	2.062409
H	0.668955	3.763410	0.946156
C	-3.019598	1.382815	-0.079984
O	-3.700014	2.132458	0.577909
O	-2.233782	1.810957	-1.096503
C	-2.995078	-0.129496	0.039689
C	-1.499709	-0.777510	0.250000
H	-1.438385	-0.986548	1.324901
H	-1.522511	-1.741479	-0.262023
C	-2.224083	3.231667	-1.325008
H	-1.511127	3.393021	-2.130910
H	-1.915119	3.760127	-0.422359
H	-3.217007	3.576654	-1.617822
H	-3.331143	-0.495198	-0.934388
C	-6.304901	-0.890631	-0.107439
O	-5.775258	-1.458138	-1.034465
O	-7.641534	-0.712405	-0.020935
C	-5.601880	-0.288228	1.083749
C	-4.032366	-0.617723	1.136520
H	-3.939895	-1.707281	1.183123
H	-3.682744	-0.230763	2.097709
C	-8.423081	-1.227071	-1.114802
H	-9.456173	-0.990795	-0.869449
H	-8.287311	-2.305528	-1.207575
H	-8.131919	-0.748027	-2.050679
H	-5.763226	0.791609	1.081481
H	-6.054867	-0.679289	1.997596

Methyl acrylate beta-scission TS

Standard orientation Energy= -1533.47248204

C	-2.134383	-0.922173	-1.428898
O	-1.573843	-1.880484	-1.908451
O	-2.916450	-0.089382	-2.146646
C	-2.057356	-0.509893	0.032454
C	-2.671210	-1.604173	0.929383
H	-2.105901	-2.530667	0.789283
H	-2.547411	-1.295892	1.971220
C	-3.048197	-0.392841	-3.549483
H	-3.708397	0.372631	-3.950840
H	-3.480564	-1.384864	-3.686330
H	-2.074329	-0.354888	-4.038971
H	-2.610902	0.420051	0.161837
C	-5.031914	-0.700617	0.993998
O	-4.809169	0.124796	1.847767
O	-6.139502	-0.683507	0.221665
C	-4.156234	-1.903425	0.676812
C	-7.074118	0.381833	0.477410
H	-7.888502	0.229071	-0.227375
H	-6.600699	1.350597	0.312684
H	-7.439622	0.331407	1.504050
H	-4.318194	-2.156986	-0.374825
C	-4.633609	-3.097382	1.530273
H	-4.047255	-3.986579	1.286822
H	-4.508665	-2.886790	2.595828
H	-5.685836	-3.322190	1.343032
C	-0.171544	2.245381	-0.067861
O	0.332373	3.192895	-0.634581
O	-1.088205	2.385124	0.920080
C	0.112319	0.818544	-0.357686
C	-0.575595	-0.270129	0.426793
H	-0.028211	-1.206094	0.289035
H	-0.558785	-0.025603	1.492087
C	-1.455924	3.732674	1.257411

H	-2.213820	3.641022	2.032343
H	-1.857790	4.248598	0.384119
H	-0.590343	4.283307	1.629083
C	3.473905	1.630409	-0.138592
O	4.155509	2.282695	-0.908404
O	2.971518	2.132173	1.016342
C	3.093554	0.231638	-0.338786
C	1.041424	0.537865	-1.323450
H	1.416539	1.335420	-1.952566
H	1.134335	-0.477545	-1.686343
C	3.215787	3.531392	1.245274
H	2.807952	3.738831	2.232808
H	2.706289	4.126951	0.486634
H	4.285486	3.743509	1.220807
H	2.709746	-0.295698	0.524730
C	5.277467	-1.959944	0.168946
O	4.323806	-2.544743	0.625154
O	6.538951	-2.142229	0.612009
C	5.242546	-0.959110	-0.966147
C	3.815737	-0.557885	-1.389647
H	3.253223	-1.465454	-1.620451
H	3.897559	0.039420	-2.300898
C	6.706352	-3.099189	1.675464
H	7.772262	-3.107927	1.891778
H	6.370599	-4.086834	1.356430
H	6.138219	-2.794167	2.555219
H	5.833653	-0.083095	-0.688601
H	5.765419	-1.416080	-1.814106

Methyl acrylate beta-scission TS with reduced flexibility set to 10.6 angstrom

Standard orientation Energy= -1533.47242932

C	2.148684	-1.060077	1.364258
O	1.589654	-2.051626	1.772589
O	2.921167	-0.274892	2.143648
C	2.081818	-0.549368	-0.066271
C	2.730089	-1.567050	-1.027715
H	2.185433	-2.513755	-0.958460
H	2.610926	-1.191010	-2.047608
C	3.044779	-0.673900	3.523083
H	3.697181	0.065817	3.981442
H	3.482489	-1.670463	3.594328
H	2.067202	-0.675714	4.006610
H	2.620748	0.396065	-0.124057
C	5.068059	-0.603545	-0.993485
O	4.834457	0.276363	-1.787881
O	6.166187	-0.616326	-0.207752
C	4.219516	-1.847454	-0.776286
C	7.076707	0.487048	-0.373069
H	7.886790	0.302755	0.329126
H	6.577456	1.429452	-0.144196
H	7.454708	0.520719	-1.395863
H	4.374460	-2.171567	0.256879
C	4.741119	-2.964168	-1.705680
H	4.175279	-3.883464	-1.536777
H	4.626401	-2.680275	-2.755252
H	5.796053	-3.174674	-1.517278
C	0.190485	2.178655	0.164091
O	-0.316583	3.094487	0.778109
O	1.115823	2.370879	-0.806726
C	-0.099631	0.738546	0.371759
C	0.600725	-0.305268	-0.461611
H	0.060589	-1.251494	-0.375224
H	0.589808	-0.007062	-1.513257
C	1.491191	3.733857	-1.064217
H	2.258085	3.682718	-1.833992

H	1.883916	4.199073	-0.158885
H	0.631638	4.307305	-1.414816
C	-3.432386	1.614307	0.141070
O	-4.102551	2.276833	0.912046
O	-2.902103	2.116259	-1.001294
C	-3.100169	0.200995	0.326128
C	-1.039925	0.409692	1.311797
H	-1.410988	1.173258	1.983964
H	-1.143279	-0.624201	1.615227
C	-3.109995	3.523488	-1.216627
H	-2.673921	3.734801	-2.191217
H	-2.607584	4.097852	-0.437224
H	-4.175215	3.758665	-1.215439
H	-2.737685	-0.330905	-0.543821
C	-5.411724	-1.857306	-0.192108
O	-4.497584	-2.478016	-0.680680
O	-6.689287	-1.967259	-0.612625
C	-5.304560	-0.884929	0.962857
C	-3.851067	-0.571061	1.370557
H	-3.334739	-1.511820	1.574638
H	-3.888155	0.010046	2.295250
C	-6.924846	-2.890356	-1.692907
H	-7.993893	-2.841592	-1.887353
H	-6.632535	-3.900378	-1.402415
H	-6.359498	-2.593904	-2.577378
H	-5.850141	0.028287	0.713850
H	-5.838183	-1.330773	1.809968

Methyl acrylate beta-scission TS with reduced flexibility set to 10.8 angstrom

Standard orientation Energy= -1533.47223244

C	2.162371	-1.218336	1.266988
O	1.607539	-2.245370	1.582679
O	2.920585	-0.497459	2.119324
C	2.107159	-0.587484	-0.115352
C	2.797334	-1.502960	-1.148786
H	2.277559	-2.465811	-1.172210
H	2.685241	-1.042553	-2.134293
C	3.034833	-1.013672	3.460109
H	3.675597	-0.310856	3.987721
H	3.481532	-2.008744	3.449174
H	2.052721	-1.066250	3.931458
H	2.627092	0.369519	-0.081968
C	5.105578	-0.482921	-0.975460
O	4.864007	0.458609	-1.693174
O	6.185407	-0.536633	-0.166557
C	4.290246	-1.764852	-0.893587
C	7.066047	0.602006	-0.208404
H	7.865089	0.377085	0.494604
H	6.533809	1.505468	0.091970
H	7.466083	0.739082	-1.214045
H	4.435324	-2.179831	0.107978
C	4.865525	-2.773450	-1.911527
H	4.324418	-3.720315	-1.844214
H	4.765231	-2.394319	-2.932068
H	5.921946	-2.970873	-1.717539
C	0.206537	2.100788	0.265366
O	-0.305449	2.975696	0.932665
O	1.141086	2.354205	-0.682239
C	-0.087853	0.650561	0.376395
C	0.627147	-0.336400	-0.512571
H	0.095400	-1.290921	-0.489174
H	0.623419	0.025688	-1.544028
C	1.523409	3.729788	-0.845208
H	2.302638	3.725744	-1.604186
H	1.902255	4.134466	0.094438

H	0.670884	4.327423	-1.171835
C	-3.392262	1.590775	0.155017
O	-4.046853	2.258042	0.935042
O	-2.839587	2.099402	-0.973735
C	-3.107906	0.163411	0.312998
C	-1.038554	0.265843	1.285388
H	-1.404052	0.985924	2.006721
H	-1.150673	-0.785292	1.519007
C	-3.011980	3.514853	-1.165011
H	-2.551456	3.735321	-2.126253
H	-2.514774	4.062995	-0.363712
H	-4.071720	3.773438	-1.181092
H	-2.772984	-0.365664	-0.569685
C	-5.542937	-1.748979	-0.215608
O	-4.673283	-2.398955	-0.745635
O	-6.833960	-1.781316	-0.607453
C	-5.359428	-0.820108	0.965066
C	-3.882286	-0.597508	1.349425
H	-3.413376	-1.569699	1.517288
H	-3.871061	-0.042521	2.290844
C	-7.141092	-2.656139	-1.709640
H	-8.210479	-2.547584	-1.876048
H	-6.893964	-3.688404	-1.457953
H	-6.581965	-2.360133	-2.598213
H	-5.858411	0.128890	0.755863
H	-5.898570	-1.262340	1.810497

Methyl acrylate beta-scission TS with reduced flexibility set to 11.0 angstrom
Standard orientation Energy= -1533.47184287

C	2.180452	-1.373014	1.152039
O	1.641587	-2.434093	1.366332
O	2.908395	-0.714995	2.078583
C	2.136914	-0.624641	-0.170821
C	2.872894	-1.428009	-1.265205
H	2.384611	-2.400467	-1.382468
H	2.764396	-0.887216	-2.209406
C	3.010265	-1.344708	3.370962
H	3.626694	-0.678583	3.970292
H	3.478090	-2.326024	3.281533
H	2.021451	-1.457864	3.816989
H	2.634038	0.336242	-0.042494
C	5.141909	-0.355156	-0.945287
O	4.886081	0.639040	-1.582590
O	6.202860	-0.447154	-0.115182
C	4.369450	-1.664853	-1.001176
C	7.044539	0.718240	-0.030559
H	7.833719	0.456140	0.670707
H	6.474444	1.573062	0.335533
H	7.463675	0.958956	-1.008572
H	4.507874	-2.165963	-0.038826
C	5.004031	-2.554585	-2.093180
H	4.495600	-3.521120	-2.128488
H	4.914368	-2.085056	-3.076415
H	6.061854	-2.733765	-1.889585
C	0.219449	2.008097	0.363376
O	-0.299769	2.834547	1.084668
O	1.163254	2.326828	-0.554819
C	-0.076473	0.553636	0.369941
C	0.657996	-0.367860	-0.572695
H	0.137672	-1.327810	-0.618032
H	0.662661	0.060793	-1.578318
C	1.549940	3.709514	-0.615398
H	2.341009	3.756303	-1.360540
H	1.914536	4.046672	0.355983
H	0.703462	4.330675	-0.912597
C	-3.352355	1.559380	0.168855
O	-3.988433	2.228347	0.962366
O	-2.781289	2.077067	-0.946567
C	-3.115787	0.119494	0.293685
C	-1.039292	0.110331	1.240477
H	-1.399033	0.778980	2.012342
H	-1.159061	-0.954233	1.397900
C	-2.918921	3.499851	-1.108916
H	-2.434010	3.731566	-2.055407
H	-2.428419	4.019480	-0.284833
H	-3.972424	3.781798	-1.141021
H	-2.813088	-0.401610	-0.605287
C	-5.671248	-1.636271	-0.236413
O	-4.850678	-2.306961	-0.816605
O	-6.973322	-1.591733	-0.588822
C	-5.407596	-0.761846	0.970171
C	-3.910461	-0.633258	1.322070
H	-3.491394	-1.634205	1.447273
H	-3.847796	-0.112336	2.280953
C	-7.355074	-2.407849	-1.712450
H	-8.422405	-2.242439	-1.841006
H	-7.150958	-3.459617	-1.507225
H	-6.808882	-2.104611	-2.606629
H	-5.857521	0.220061	0.807386
H	-5.948442	-1.203920	1.814481

Methyl acrylate beta-scission TS with reduced flexibility set to 11.2 angstrom
Standard orientation Energy= -1533.47123871

C	2.195160	-1.537986	0.999408
O	1.669378	-2.623015	1.090568
O	2.897946	-0.972378	2.003394
C	2.162808	-0.654098	-0.237455
C	2.949644	-1.316732	-1.390892
H	2.496899	-2.285795	-1.622911
H	2.845605	-0.682320	-2.275430
C	2.990089	-1.735370	3.222576
H	3.584186	-1.127337	3.900998
H	3.477420	-2.693516	3.037246
H	1.996355	-1.912368	3.635687
H	2.633253	0.297926	0.005102
C	5.171788	-0.213145	-0.890928
O	4.901253	0.834946	-1.427978
O	6.209939	-0.360258	-0.040248
C	4.447801	-1.533149	-1.111877
C	7.008212	0.814899	0.195217
H	7.785073	0.503751	0.890159
H	6.398626	1.607379	0.631257
H	7.447256	1.172000	-0.737443
H	4.579183	-2.131790	-0.205990
C	5.146807	-2.275446	-2.274044
H	4.675904	-3.248751	-2.431921
H	5.068893	-1.703231	-3.202306
H	6.203914	-2.440103	-2.055295
C	0.227863	1.906812	0.469318
O	-0.300245	2.673231	1.248151
O	1.182813	2.297399	-0.408706
C	-0.069397	0.456778	0.355477
C	0.685947	-0.385520	-0.644173
H	0.177549	-1.344519	-0.770375
H	0.700263	0.119043	-1.613843
C	1.574692	3.678745	-0.350360
H	2.380680	3.781263	-1.073579
H	1.920823	3.935116	0.651931
H	0.736097	4.325666	-0.613076
C	-3.311518	1.530384	0.175928

O	-3.930068	2.204206	0.978779
O	-2.716308	2.051730	-0.925251
C	-3.127429	0.079904	0.271639
C	-1.045816	-0.049574	1.177726
H	-1.397476	0.555431	2.003813
H	-1.173428	-1.122747	1.247389
C	-2.815129	3.480152	-1.063284
H	-2.299605	3.717885	-1.991924
H	-2.336472	3.972805	-0.215981
H	-3.860441	3.788302	-1.118067
H	-2.862242	-0.434558	-0.642979
C	-5.791419	-1.522976	-0.245289
O	-5.019689	-2.213383	-0.867904
O	-7.098903	-1.406629	-0.559574
C	-5.454080	-0.698764	0.977921
C	-3.943207	-0.658268	1.295830
H	-3.574637	-1.682622	1.385869
H	-3.833155	-0.168381	2.266858
C	-7.548807	-2.168533	-1.695903
H	-8.610086	-1.949405	-1.789554
H	-7.389687	-3.234610	-1.528055
H	-7.012733	-1.863519	-2.595592
H	-5.854432	0.309737	0.853694
H	-5.996576	-1.136212	1.823456

Methyl acrylate beta-scission TS with reduced flexibility set to 11.4 angstrom

Standard orientation Energy= -1533.47025179

C	2.210107	-1.695155	0.809018
O	1.698982	-2.789859	0.761558
O	2.887673	-1.243896	1.885881
C	2.187140	-0.671610	-0.315315
C	3.028557	-1.172008	-1.513593
H	2.614876	-2.119336	-1.873304
H	2.930126	-0.439938	-2.319848
C	2.971742	-2.145637	3.006917
H	3.546109	-1.615450	3.763174
H	3.475823	-3.068664	2.717410
H	1.974299	-2.383274	3.378621
H	2.627159	0.254496	0.051835
C	5.195528	-0.071983	-0.802752
O	4.907914	1.025813	-1.217904
O	6.208790	-0.291810	0.062043
C	4.525493	-1.375943	-1.210950
C	6.957950	0.868576	0.469352
H	7.720938	0.497380	1.150037
H	6.307201	1.582739	0.975549
H	7.416006	1.350845	-0.395372
H	4.647964	-2.077503	-0.380865
C	5.294071	-1.946257	-2.427246
H	4.864549	-2.906349	-2.723497
H	5.230775	-1.265310	-3.280123
H	6.347090	-2.103937	-2.185332
C	0.231121	1.797163	0.574080
O	-0.305096	2.491005	1.413129
O	1.194334	2.264585	-0.256029
C	-0.066314	0.363460	0.327724
C	0.712698	-0.386849	-0.726961
H	0.217753	-1.336810	-0.942347
H	0.737068	0.198221	-1.650090
C	1.589904	3.633123	-0.066589
H	2.408537	3.796259	-0.763954
H	1.919856	3.796173	0.960339
H	0.758005	4.305316	-0.283298
C	-3.267103	1.505820	0.160196
O	-3.869502	2.196365	0.960662

O	-2.639167	2.014024	-0.929227
C	-3.142206	0.047374	0.244572
C	-1.058591	-0.206961	1.090923
H	-1.399303	0.321885	1.971927
H	-1.193970	-1.281297	1.063968
C	-2.693706	3.445110	-1.061495
H	-2.137858	3.674015	-1.968797
H	-2.235650	3.921100	-0.193480
H	-3.727623	3.782097	-1.153009
H	-2.923774	-0.470025	-0.680877
C	-5.906262	-1.411152	-0.227975
O	-5.183230	-2.125338	-0.881558
O	-7.215841	-1.234102	-0.503204
C	-5.498876	-0.619108	0.994883
C	-3.979130	-0.658821	1.276575
H	-3.661612	-1.701560	1.349434
H	-3.823873	-0.186385	2.250156
C	-7.729900	-1.959146	-1.636363
H	-8.782713	-1.692689	-1.697221
H	-7.613250	-3.033477	-1.487230
H	-7.205929	-1.664871	-2.546701
H	-5.849191	0.409947	0.890545
H	-6.041572	-1.037873	1.849526

Methyl acrylate beta-scission TS with reduced flexibility set to 11.6 angstrom

Standard orientation Energy= -1533.47032190

C	-2.204244	-1.666562	-0.586861
O	-1.830863	-2.760116	-0.230537
O	-2.599474	-1.396393	-1.848231
C	-2.290255	-0.442027	0.310933
C	-3.294738	-0.695561	1.460650
H	-2.961284	-1.562349	2.039377
H	-3.279848	0.174086	2.123323
C	-2.540954	-2.493785	-2.780898
H	-2.892142	-2.088837	-3.727243
H	-3.183200	-3.310758	-2.449417
H	-1.517150	-2.857694	-2.875091
H	-2.637641	0.401871	-0.284827
C	-5.323179	0.281050	0.307296
O	-5.068500	1.429170	0.584189
O	-6.212845	-0.068332	-0.646713
C	-4.745016	-0.944605	0.999673
C	-6.871468	1.016802	-1.326984
H	-7.541171	0.546410	-2.043572
H	-6.140577	1.643798	-1.839223
H	-7.433497	1.627032	-0.618557
H	-4.770385	-1.767164	0.279137
C	-5.685561	-1.322265	2.171710
H	-5.323230	-2.228513	2.662927
H	-5.722205	-0.520706	2.914197
H	-6.699171	-1.510472	1.812176
C	0.014745	1.614507	-0.881107
O	0.735303	2.008889	-1.775526
O	-0.984521	2.362884	-0.359407
C	0.113426	0.284969	-0.229958
C	-0.867290	-0.103126	0.853404
H	-0.483059	-0.972353	1.392529
H	-0.981192	0.718588	1.565352
C	-1.147155	3.678508	-0.915981
H	-2.004042	4.106524	-0.400525
H	-1.332913	3.618472	-1.989341
H	-0.252026	4.277161	-0.740554
C	2.814999	0.442061	1.499388
O	2.786210	1.626151	1.772381
O	2.508464	-0.528570	2.401971

C	3.150174	-0.122770	0.188526
C	1.124481	-0.548655	-0.664566
H	1.583724	-0.338072	-1.620984
H	1.137563	-1.574482	-0.318384
C	2.153852	-0.071501	3.716708
H	1.943152	-0.971334	4.291128
H	1.273800	0.572780	3.677571
H	2.978991	0.483913	4.165837
H	3.396697	-1.177718	0.184978
C	6.165249	-0.347559	-0.722638
O	5.750094	-1.383483	-1.185375
O	7.440632	-0.180123	-0.312383
C	5.366975	0.925028	-0.539587
C	3.852612	0.754188	-0.811671
H	3.733895	0.328998	-1.810781
H	3.391706	1.742995	-0.814757
C	8.311366	-1.316869	-0.466671
H	9.278834	-0.993987	-0.088673
H	8.384793	-1.601720	-1.517130
H	7.937353	-2.164535	0.109047
H	5.552200	1.319097	0.463028
H	5.777914	1.667369	-1.232715

Methyl acrylate beta-scission TS with reduced flexibility set to 11.8 angstrom

Standard orientation Energy= -1533.46807506

C	2.219503	-1.788710	-0.296366
O	1.873065	-2.608757	-1.114635
O	2.565616	-2.108225	0.968090
C	2.319396	-0.292948	-0.552489
C	3.362581	-0.012226	-1.668197
H	3.059215	-0.534597	-2.580507
H	3.354278	1.060753	-1.878360
C	2.487299	-3.505208	1.314252
H	2.796448	-3.563543	2.355363
H	3.153375	-4.092844	0.681124
H	1.466024	-3.869134	1.195797
H	2.648842	0.194324	0.364771
C	5.334892	0.346997	-0.123185
O	5.075672	1.500397	0.126439
O	6.187799	-0.394350	0.616360
C	4.806098	-0.440661	-1.313335
C	6.800505	0.272663	1.736041
H	7.445544	-0.470695	2.199312
H	6.039165	0.611757	2.439658
H	7.383566	1.130403	1.397709
H	4.816924	-1.497808	-1.032681
C	5.816596	-0.264177	-2.483129
H	5.495727	-0.854160	-3.344954
H	5.880028	0.783738	-2.787558
H	6.810532	-0.603361	-2.185596
C	0.044823	1.054632	1.412509
O	-0.646822	1.005751	2.409389
O	1.042604	1.955076	1.254157
C	-0.093870	0.161262	0.235372
C	0.897445	0.258391	-0.907348
H	0.512066	-0.291909	-1.768861
H	1.024761	1.304364	-1.199805
C	1.239195	2.878621	2.338593
H	2.090165	3.490017	2.046748
H	1.450097	2.340787	3.264038
H	0.349127	3.494234	2.477141
C	-2.885534	1.254415	-0.944483
O	-2.918007	2.386467	-0.504671
O	-2.546553	0.975140	-2.231474
C	-3.175154	0.032848	-0.182115

C	-1.149065	-0.739992	0.269171
H	-1.563805	-0.982413	1.238562
H	-1.196738	-1.504789	-0.496002
C	-2.228712	2.108556	-3.054767
H	-1.981248	1.698781	-4.031962
H	-1.380334	2.658589	-2.643865
H	-3.084419	2.781885	-3.128621
H	-3.405114	-0.839016	-0.782944
C	-6.188640	-0.776360	0.434364
O	-5.728277	-1.873707	0.225772
O	-7.479050	-0.458708	0.196071
C	-5.436799	0.407356	1.002574
C	-3.906177	0.170524	1.130860
H	-3.757286	-0.738806	1.718294
H	-3.479502	1.003361	1.692384
C	-8.308510	-1.517077	-0.319822
H	-9.295318	-1.076900	-0.444612
H	-8.345498	-2.350308	0.383280
H	-7.922467	-1.872234	-1.276216
H	-5.655665	1.291369	0.398183
H	-5.852095	0.612051	1.995188

Methyl acrylate beta-scission TS with reduced flexibility set to 12.0 angstrom

Standard orientation Energy= -1533.46424683

C	2.264699	1.856963	0.261528
O	1.962819	2.734649	1.036419
O	2.567157	2.087249	-1.033394
C	2.346165	0.378948	0.609790
C	3.408868	0.152523	1.729901
H	3.132744	0.741331	2.609607
H	3.383662	-0.903610	2.011405
C	2.496434	3.459768	-1.467667
H	2.762695	3.444204	-2.522064
H	3.197704	4.074933	-0.902291
H	1.486958	3.849686	-1.331964
H	2.661026	-0.168309	-0.277857
C	5.338233	-0.340922	0.164694
O	5.056412	-1.504029	-0.002118
O	6.176076	0.338677	-0.648133
C	4.859521	0.528564	1.318656
C	6.743614	-0.407749	-1.741113
H	7.384025	0.294281	-2.270557
H	5.955759	-0.778425	-2.398072
H	7.324963	-1.251572	-1.366746
H	4.878760	1.565483	0.971014
C	5.925676	0.413188	2.459062
H	5.648877	1.060209	3.294685
H	5.994732	-0.614320	2.824855
H	6.904657	0.723706	2.090523
C	0.114985	-1.077378	-1.287497
O	-0.519068	-1.068147	-2.322791
O	1.094634	-1.976867	-1.034224
C	-0.083071	-0.131843	-0.159404
C	0.906976	-0.127406	0.996261
H	0.518654	0.496339	1.804593
H	1.023390	-1.144992	1.380769
C	1.346950	-2.944408	-2.067996
H	2.176353	-3.546479	-1.703746
H	1.613815	-2.444858	-3.000403
H	0.461980	-3.560848	-2.233052
C	-2.951191	-1.367436	0.720297
O	-3.067050	-2.436622	0.156140
O	-2.547029	-1.258962	2.013304
C	-3.203732	-0.049172	0.116333
C	-1.189434	0.716689	-0.269396

H	-1.547944	0.905449	-1.273281
H	-1.279387	1.527467	0.443191
C	-2.258191	-2.496009	2.684344
H	-1.952648	-2.217056	3.690783
H	-1.455820	-3.032994	2.175402
H	-3.144524	-3.131905	2.716944
H	-3.414157	0.738960	0.829743
C	-6.248678	0.867035	-0.332365
O	-5.760767	1.909414	0.035083
O	-7.547402	0.551040	-0.141895
C	-5.525809	-0.239681	-1.067589
C	-3.984084	-0.010390	-1.182773
H	-3.830062	0.964093	-1.653660
H	-3.585693	-0.773017	-1.854968
C	-8.350440	1.542060	0.526995
H	-9.348434	1.113678	0.586991
H	-8.364666	2.471065	-0.044608
H	-7.957190	1.741305	1.524745
H	-5.751246	-1.194739	-0.586506
H	-5.950195	-0.300344	-2.074961

Methyl acrylate beta-scission TS with reduced flexibility set to 12.2 angstrom
Standard orientation Energy= -1533.45830685

C	2.335392	1.914202	0.185760
O	2.113671	2.860430	0.905045
O	2.557769	2.031118	-1.140455
C	2.385050	0.465253	0.643314
C	3.462637	0.297068	1.772523
H	3.212926	0.959035	2.606567
H	3.413760	-0.733478	2.133822
C	2.497476	3.366483	-1.679358
H	2.688771	3.258023	-2.744504
H	3.254565	3.999365	-1.214450
H	1.512033	3.801387	-1.508229
H	2.681716	-0.153583	-0.202001
C	5.354499	-0.357338	0.216622
O	5.043124	-1.522571	0.141607
O	6.185719	0.241125	-0.664553
C	4.925571	0.604404	1.315550
C	6.709386	-0.596526	-1.712297
H	7.352626	0.049169	-2.306111
H	5.897704	-0.994551	-2.322606
H	7.280188	-1.424900	-1.290232
H	4.958519	1.612319	0.892062
C	6.046748	0.553895	2.425750
H	5.815038	1.265773	3.221095
H	6.118536	-0.445325	2.861736
H	7.008195	0.821908	1.985972
C	0.166278	-1.085454	-1.166805
O	-0.424794	-1.131017	-2.226137
O	1.133778	-1.970795	-0.827769
C	-0.077349	-0.082440	-0.098416
C	0.919655	0.024809	1.057021
H	0.537752	0.727114	1.801467
H	1.013597	-0.953878	1.538088
C	1.433940	-2.983360	-1.804459
H	2.247835	-3.564495	-1.376813
H	1.741146	-2.525076	-2.745590
H	0.558431	-3.609921	-1.981600
C	-3.011163	-1.412022	0.554823
O	-3.205395	-2.425789	-0.084231
O	-2.546016	-1.426139	1.830923
C	-3.230750	-0.037021	0.067857
C	-1.230183	0.713098	-0.278617
H	-1.535120	0.846838	-1.309780

H	-1.349478	1.570867	0.372679
C	-2.286588	-2.724806	2.388333
H	-1.930645	-2.541471	3.400171
H	-1.528205	-3.250262	1.805358
H	-3.198066	-3.324616	2.407143
H	-3.427574	0.681497	0.855280
C	-6.326733	0.902319	-0.241080
O	-5.826133	1.883956	0.254302
O	-7.632158	0.587370	-0.100740
C	-5.614121	-0.120181	-1.096999
C	-4.061283	0.121214	-1.201305
H	-3.914730	1.135729	-1.581992
H	-3.680414	-0.574483	-1.952255
C	-8.425561	1.501523	0.679798
H	-9.430813	1.086107	0.678813
H	-8.419436	2.494259	0.227682
H	-8.038681	1.568928	1.697465
H	-5.833493	-1.121203	-0.717699
H	-6.040609	-0.073310	-2.103564

Methyl acrylate beta-scission TS with reduced flexibility set to 12.4 angstrom
Standard orientation Energy= -1533.44996442

C	-2.417872	-1.947443	0.115445
O	-2.317639	-2.948626	0.785913
O	-2.502056	-1.966865	-1.231898
C	-2.438303	-0.529398	0.661309
C	-3.530909	-0.404292	1.796035
H	-3.304479	-1.121971	2.589126
H	-3.461231	0.600997	2.219178
C	-2.439851	-3.265375	-1.853956
H	-2.507800	-3.077979	-2.923153
H	-3.270104	-3.888248	-1.518451
H	-1.498935	-3.759849	-1.609488
H	-2.713717	0.146891	-0.145771
C	-5.391479	0.379540	0.257680
O	-5.056667	1.540817	0.255997
O	-6.212298	-0.150624	-0.676275
C	-5.007156	-0.651283	1.308905
C	-6.696726	0.756521	-1.684185
H	-7.337747	0.159043	-2.328724
H	-5.864487	1.173819	-2.252511
H	-7.262071	1.569781	-1.226824
H	-5.050823	-1.632700	0.828519
C	-6.183310	-0.646326	2.386309
H	-5.993334	-1.406974	3.146339
H	-6.260851	0.327840	2.873963
H	-7.123218	-0.875584	1.883766
C	-0.190451	1.072763	-1.074145
O	0.394864	1.169492	-2.133430
O	-1.166326	1.933328	-0.695150
C	0.072297	0.030267	-0.049968
C	-0.945013	-0.150573	1.091413
H	-0.577263	-0.910413	1.784914
H	-1.017584	0.792642	1.643018
C	-1.488435	2.975816	-1.632745
H	-2.305580	3.530749	-1.177386
H	-1.798666	2.546855	-2.586642
H	-0.622787	3.619529	-1.796606
C	3.062614	1.423606	0.447513
O	3.332206	2.392904	-0.231132
O	2.538581	1.524608	1.695961
C	3.252795	0.014317	0.042880
C	1.265398	-0.724023	-0.267305
H	1.525804	-0.833541	-1.314550
H	1.398197	-1.609553	0.343878

C	2.311749	2.860674	2.173812
H	1.903812	2.745972	3.176088
H	1.603171	3.385115	1.530537
H	3.246776	3.422529	2.204239
H	3.444099	-0.649404	0.878828
C	6.407136	-0.906540	-0.164647
O	5.900844	-1.824894	0.435280
O	7.719423	-0.600457	-0.077261
C	5.695259	0.037852	-1.105324
C	4.129627	-0.222004	-1.195509
H	3.996094	-1.261447	-1.507968
H	3.758754	0.415660	-2.001253
C	8.511991	-1.441927	0.781857
H	9.523016	-1.045039	0.723377
H	8.484215	-2.475752	0.434845
H	8.140644	-1.397891	1.806571
H	5.904371	1.068254	-0.808442
H	6.118128	-0.089834	-2.105693

Methyl acrylate MCR pentamer with radical on unit 4 and chirality RRR
Standard orientation Energy= -1533.50120926

C	3.907702	-1.708881	-0.170500
O	3.153321	-2.598634	-0.490714
O	4.725408	-1.786103	0.898527
C	4.104366	-0.411494	-0.938867
C	4.985672	-0.733463	-2.165781
H	4.464685	-1.412132	-2.845661
H	5.928148	-1.200253	-1.868358
C	4.663804	-3.007547	1.660953
H	5.381460	-2.880859	2.468287
H	4.932789	-3.860311	1.036144
H	3.659537	-3.156181	2.059651
H	4.656272	0.273806	-0.290296
C	-4.885759	0.169962	-1.746599
O	-5.280391	1.250397	-2.113114
O	-5.350086	-0.996829	-2.246622
C	-3.823298	-0.081456	-0.700108
C	-3.382568	1.217832	0.005152
H	-3.000400	1.909628	-0.748807
H	-4.266328	1.680222	0.451185
C	-6.349655	-0.889870	-3.277092
H	-6.595470	-1.913839	-3.549961
H	-5.954556	-0.347982	-4.137545
H	-7.232107	-0.370123	-2.901253
H	-2.973721	-0.559573	-1.196029
C	-2.705425	0.607159	2.411902
O	-1.927674	0.523181	3.349952
O	-4.035494	0.342457	2.544259
C	-2.337088	0.999026	1.057933
C	-0.885946	1.279296	0.789864
H	-0.546987	2.054533	1.489471
H	-0.790565	1.678061	-0.220923
C	-4.468067	-0.034431	3.860889
H	-5.540461	-0.199839	3.778846
H	-4.257093	0.761490	4.577005
H	-3.962970	-0.945907	4.185290
C	-0.198595	-1.013978	-0.047345
O	-0.540997	-0.828820	-1.195841
O	0.009632	-2.232251	0.476882
C	0.075122	0.076941	0.972557
C	1.567296	0.509501	0.943820
H	1.705576	1.216118	1.764076
H	2.179864	-0.357229	1.201156
C	-0.093018	-3.354502	-0.425395
H	-0.026836	-4.237233	0.206585
H	0.736997	-3.333143	-1.130787
H	-1.044128	-3.329487	-0.957410
H	-0.095721	-0.348405	1.961506
C	2.989158	2.348831	-0.052987
O	3.411521	2.664000	1.033318
O	3.281396	3.039578	-1.178462
C	2.076133	1.163331	-0.361447
C	2.763665	0.214875	-1.381113
H	2.944751	0.795016	-2.287961
H	2.068417	-0.579827	-1.656769
C	4.131658	4.190171	-1.013392
H	4.250214	4.608604	-2.010508
H	5.098267	3.895955	-0.602170
H	3.665533	4.915804	-0.345565
H	1.229119	1.595575	-0.903099
H	5.217587	0.186363	-2.707527
H	-4.215886	-0.802466	0.021271

Methyl acrylate MCR pentamer with radical on unit 4 and chirality RRS
Standard orientation Energy= -1533.50336288

C	3.680006	1.488041	1.364385
O	2.755168	2.266823	1.375331
O	4.386506	1.172986	2.473092
C	4.229016	0.774412	0.139069
C	5.404775	1.599840	-0.425725
H	5.845627	1.077590	-1.277920
H	6.184398	1.744740	0.325160
C	3.998382	1.845134	3.685445
H	4.669831	1.468580	4.454069
H	2.961758	1.614558	3.935038
H	4.107632	2.925100	3.575666
H	4.626087	-0.189236	0.467915
C	-4.470233	0.265561	1.581910
O	-5.589294	0.407552	1.152593
O	-4.199380	0.012033	2.880038
C	-3.196305	0.316079	0.764627
C	-3.410900	1.013750	-0.592840
H	-4.232538	0.496780	-1.106177
H	-3.743608	2.035862	-0.421723
C	-5.339154	-0.104225	3.753514
H	-4.928115	-0.302920	4.740736
H	-5.983502	-0.924084	3.433048
H	-5.913497	0.822993	3.755542
H	-2.868025	-0.720565	0.633118
C	-1.608085	2.241293	-1.977570
O	-0.708526	2.294700	-2.802761
O	-2.149122	3.358359	-1.423884
C	-2.209809	1.005476	-1.490540
C	-1.671573	-0.296627	-2.003698
H	-1.311383	-0.172221	-3.027742
H	-2.494960	-1.015940	-2.020825
C	-1.587114	4.606394	-1.861212
H	-2.140533	5.376115	-1.327276
H	-1.707449	4.724238	-2.939262
H	-0.525649	4.655667	-1.613184
C	-0.583895	-2.419693	-1.275750
O	0.068508	-3.114270	-2.014986
O	-1.563337	-2.907815	-0.477177
C	-0.491429	-0.906098	-1.150353
C	0.874299	-0.350623	-1.585539
H	1.212395	-0.888098	-2.476290
H	0.735523	0.688798	-1.883525
C	-1.815534	-4.322267	-0.575690
H	-2.624900	-4.523545	0.122814
H	-0.924846	-4.889289	-0.302811

H	-2.110422	-4.588055	-1.591833
H	-0.671245	-0.667775	-0.100238
C	2.498593	-1.776445	-0.257843
O	3.450436	-2.285356	-0.797174
O	1.740514	-2.423670	0.659998
C	1.988226	-0.365070	-0.505192
C	3.142110	0.561843	-0.924358
H	2.718671	1.533522	-1.191097
H	3.616239	0.150924	-1.819597
C	2.105760	-3.793625	0.916725
H	1.411901	-4.141474	1.679455
H	3.132807	-3.852282	1.278968
H	2.011268	-4.384891	0.004982
H	1.550750	-0.000770	0.429097
H	5.063400	2.581431	-0.766063
H	-2.422212	0.814603	1.353191

Methyl acrylate MCR pentamer with radical on unit 4 and chirality RSR
Standard orientation Energy= -1533.50890723

C	-4.630964	-0.356933	-1.039282
O	-4.860950	0.828061	-1.058209
O	-5.428816	-1.255534	-0.421480
C	-3.462943	-1.039984	-1.734208
C	-3.902232	-1.428141	-3.161831
H	-4.144228	-0.537680	-3.748028
H	-4.779476	-2.078488	-3.142653
C	-6.603330	-0.720904	0.218178
H	-7.110907	-1.577504	0.656387
H	-7.244904	-0.226595	-0.512708
H	-6.322595	-0.003524	0.990323
H	-3.250234	-1.962202	-1.185985
C	3.648767	-2.171325	0.833590
O	3.868117	-2.084978	2.018794
O	3.826493	-3.310443	0.130543
C	3.122992	-1.064950	-0.052725
C	3.367721	0.330319	0.558902
H	2.994249	0.335037	1.584917
H	4.450896	0.489429	0.619159
C	4.260695	-4.457644	0.883357
H	4.348192	-5.263911	0.158331
H	3.527665	-4.710531	1.650851
H	5.222969	-4.261638	1.358374
H	2.052126	-1.255812	-0.185305
C	3.237988	1.855358	-1.522920
O	2.766021	2.747911	-2.208423
O	4.309730	1.116789	-1.928074
C	2.749399	1.462522	-0.207434
C	1.673206	2.296322	0.413987
H	1.454214	3.153158	-0.224893
H	2.051873	2.675424	1.372859
C	4.851863	1.458932	-3.213579
H	5.689371	0.781100	-3.366237
H	5.191682	2.495870	-3.223422
H	4.102182	1.324475	-3.995151
C	-0.525105	2.417875	1.572805
O	-1.353488	3.195909	1.167728
O	-0.226949	2.265781	2.882003
C	0.347370	1.522749	0.704787
C	-0.369847	1.117683	-0.590416
H	-0.692411	2.021572	-1.112640
H	0.345870	0.605452	-1.239064
C	-0.947510	3.114312	3.797139
H	-0.583571	2.848241	4.787055
H	-2.020125	2.932834	3.719117
H	-0.745517	4.164597	3.582380

H	0.597357	0.639743	1.299557
C	-1.200991	-1.068974	0.325667
O	-0.293925	-1.800549	-0.005173
O	-1.986271	-1.313439	1.393660
C	-1.599337	0.207486	-0.396453
C	-2.218826	-0.139855	-1.769605
H	-1.456584	-0.635448	-2.379013
H	-2.477473	0.799274	-2.265883
C	-1.697298	-2.514405	2.138001
H	-2.430970	-2.540537	2.940337
H	-0.685528	-2.475817	2.543352
H	-1.795817	-3.391938	1.497902
H	-2.345883	0.726693	0.207549
H	-3.094464	-1.961743	-3.668563
H	3.582616	-1.148236	-1.037611

Methyl acrylate MCR pentamer with radical on unit 4 and chirality RSS
Standard orientation Energy= -1533.50438211

C	-4.541139	-0.327895	-1.001422
O	-4.012241	-0.750361	-2.002003
O	-5.605674	-0.925091	-0.421786
C	-4.175149	0.955997	-0.274068
C	-5.120497	2.068394	-0.782888
H	-4.943088	2.991547	-0.226259
H	-6.167439	1.787578	-0.652288
C	-6.116263	-2.095700	-1.084254
H	-6.967125	-2.419294	-0.488579
H	-5.356415	-2.877372	-1.121198
H	-6.428943	-1.854305	-2.101252
H	-4.380151	0.797975	0.788783
C	4.003542	-2.221169	-0.650528
O	5.083454	-2.076363	-1.169608
O	3.476335	-3.431806	-0.362640
C	3.059147	-1.113985	-0.233145
C	3.652429	0.279555	-0.497086
H	4.574472	0.376604	0.096989
H	3.962776	0.347503	-1.537433
C	4.275118	-4.571940	-0.729903
H	3.696704	-5.441477	-0.425406
H	5.235297	-4.548928	-0.212536
H	4.448991	-4.585066	-1.806740
H	2.827473	-1.269179	0.824468
C	2.585894	2.559355	-1.064922
O	1.957015	3.572206	-0.800006
O	3.205862	2.384373	-2.261958
C	2.755017	1.430817	-0.153547
C	2.123995	1.550071	1.201301
H	1.987856	2.608360	1.436148
H	2.800634	1.116152	1.944981
C	3.069227	3.463508	-3.199012
H	3.624126	3.152857	-4.081915
H	3.485760	4.384540	-2.787732
H	2.018676	3.627745	-3.444715
C	0.328193	0.946484	2.820455
O	-0.450094	1.744553	3.284299
O	0.983660	0.033122	3.572829
C	0.733179	0.839358	1.357004
C	-0.341464	1.420951	0.430894
H	-0.561072	2.444172	0.742045
H	0.056577	1.469677	-0.583667
C	0.723187	0.073827	4.989270
H	1.325612	-0.723713	5.418556
H	-0.335988	-0.096104	5.186626
H	1.013965	1.040461	5.402874
H	0.875204	-0.220480	1.142250

C	-1.402917	-0.784941	-0.126620
O	-0.563021	-1.085328	-0.942257
O	-2.235864	-1.686912	0.436450
C	-1.655900	0.620297	0.400004
C	-2.705601	1.363054	-0.468861
H	-2.438571	1.276748	-1.526162
H	-2.626139	2.422206	-0.207849
C	-2.123320	-3.036972	-0.056364
H	-2.841012	-3.616602	0.520652
H	-1.112499	-3.415915	0.098304
H	-2.363657	-3.066611	-1.119720
H	-2.058937	0.531508	1.412733
H	-4.945587	2.270933	-1.843135
H	2.116983	-1.253056	-0.773230

Methyl acrylate migration TS with chirality RRR
Standard orientation Energy= -1533.47453441

C	3.704479	-1.758377	0.131863
O	4.296457	-1.342711	1.098908
O	4.116550	-1.548947	-1.136443
C	2.422570	-2.576317	0.184482
C	2.648781	-3.811198	1.072389
H	2.899097	-3.510980	2.092093
H	3.466467	-4.428043	0.690383
C	5.310527	-0.752965	-1.290178
H	5.502719	-0.724677	-2.360368
H	6.144648	-1.213145	-0.759481
H	5.145596	0.253330	-0.903006
H	2.184340	-2.899524	-0.830387
C	-4.347979	0.014167	-0.704727
O	-4.902314	1.080400	-0.817000
O	-4.806528	-1.121681	-1.279879
C	-3.092733	-0.253001	0.094541
C	-2.328718	1.036563	0.415044
H	-2.122119	1.558219	-0.523245
H	-2.986258	1.704190	0.983670
C	-6.032002	-0.999370	-2.026920
H	-6.242389	-1.997426	-2.405144
H	-5.908349	-0.294430	-2.850026
H	-6.840805	-0.655901	-1.380450
H	-2.478015	-0.968276	-0.454622
C	-1.024070	0.375762	2.573347
O	-0.065449	0.391044	3.317486
O	-2.218210	-0.150844	2.941879
C	-1.012705	0.892954	1.172803
C	-0.042727	2.055122	0.970869
H	0.645213	2.101974	1.817327
H	-0.609803	2.991684	0.944655
C	-2.281068	-0.686413	4.277488
H	-3.299131	-1.051539	4.395819
H	-2.064727	0.091601	5.010442
H	-1.563438	-1.499111	4.397479
C	1.669350	3.134878	-0.535083
O	2.876653	3.126769	-0.551608
O	0.935856	4.257454	-0.698746
C	0.775189	1.922981	-0.338838
C	1.605961	0.627736	-0.329476
H	2.184933	0.572592	-1.254697
H	2.317667	0.675144	0.497301
C	1.677015	5.476831	-0.893796
H	0.927350	6.257518	-1.002055
H	2.292513	5.408293	-1.791745
H	2.318163	5.675364	-0.033932
H	0.089345	1.903794	-1.191198
C	0.073560	-1.038202	-1.450581
O	-0.066331	-0.329613	-2.426469
O	-0.414927	-2.303629	-1.394793
C	0.741684	-0.621013	-0.182933
C	1.243744	-1.727794	0.737235
H	0.424516	-2.420286	0.942903
H	1.547328	-1.287064	1.690534
C	-1.090403	-2.770622	-2.578068
H	-1.364630	-3.801916	-2.366425
H	-0.426733	-2.717219	-3.441785
H	-1.981688	-2.172126	-2.772213
H	-0.285726	-0.084198	0.522621
H	1.744930	-4.424827	1.098638
H	-3.397791	-0.759257	1.013479

Methyl acrylate migration TS with chirality RRS
Standard orientation Energy= -1533.47018772

C	4.005578	-0.384971	-0.695265
O	4.174388	-0.269544	-1.886621
O	4.708225	0.308812	0.222315
C	3.029306	-1.347040	-0.030985
C	3.780040	-2.668641	0.243123
H	3.130499	-3.361549	0.783309
H	4.669237	-2.494731	0.852529
C	5.687629	1.233144	-0.290674
H	6.153394	1.676540	0.586485
H	5.201973	2.000573	-0.894406
H	6.426844	0.708461	-0.897444
H	2.737051	-0.923608	0.931962
C	-4.414114	-0.272868	-0.055379
O	-4.721824	-1.437266	-0.150972
O	-5.296723	0.738846	-0.197949
C	-3.015453	0.250481	0.192597
C	-2.195598	-0.720127	1.052423
H	-2.670555	-0.790133	2.039739
H	-2.276759	-1.723933	0.632681
C	-6.649543	0.358922	-0.514222
H	-7.202419	1.292609	-0.589509
H	-7.063078	-0.270934	0.274442
H	-6.681870	-0.183665	-1.460036
H	-3.088268	1.244061	0.637472
C	-0.006621	-1.260545	2.235975
O	1.054968	-1.001841	2.768271
O	-0.646208	-2.437771	2.426649
C	-0.722535	-0.370023	1.272939
C	-0.311269	1.095613	1.371975
H	0.573095	1.163616	2.008866
H	-1.107525	1.673846	1.851736
C	-0.004747	-3.369405	3.315246
H	-0.661780	-4.235757	3.348925
H	0.108902	-2.933570	4.308722
H	0.978995	-3.648236	2.934598
C	0.470541	3.175166	0.219435
O	1.606211	3.579432	0.153642
O	-0.579163	3.967778	0.531149
C	0.018655	1.742748	-0.005788
C	1.102845	0.945093	-0.756178
H	1.300455	1.428098	-1.717992
H	2.021732	1.012554	-0.175296
C	-0.272534	5.349529	0.798368
H	-1.227206	5.820663	1.021740
H	0.188967	5.812247	-0.074879
H	0.405823	5.432623	1.648669
H	-0.884166	1.788616	-0.614667
C	-0.295012	-0.716765	-2.053388
O	-0.973496	0.165702	-2.543689
O	-0.429660	-2.015665	-2.400737
C	0.724828	-0.514506	-0.981286

C	1.803803	-1.603547	-0.922660
H	2.164274	-1.786676	-1.940717
H	1.331863	-2.538982	-0.609604
C	-1.429711	-2.307983	-3.394892
H	-1.351595	-3.377698	-3.576122
H	-1.234212	-1.746655	-4.309087
H	-2.423426	-2.057100	-3.020910
H	-0.048711	-0.690474	0.124476
H	4.087557	-3.145539	-0.691689
H	-2.569356	0.381214	-0.798657

Methyl acrylate migration TS with chirality RSR
Standard orientation Energy= -1533.47030751

C	2.134438	-2.975648	-0.280223
O	1.692951	-3.762798	0.524412
O	3.432154	-2.621172	-0.339656
C	1.306731	-2.260609	-1.337819
C	0.643146	-3.292503	-2.264639
H	0.004633	-3.968711	-1.691949
H	1.393208	-3.891769	-2.787058
C	4.295337	-3.186982	0.668621
H	5.296898	-2.847793	0.414260
H	4.238836	-4.275723	0.651205
H	4.004823	-2.817179	1.652518
H	1.984104	-1.638480	-1.925456
C	-3.693959	-0.542837	-1.476566
O	-3.272386	-0.485442	-2.607894
O	-4.791314	-1.249458	-1.133676
C	-3.068776	0.112362	-0.262485
C	-2.189959	1.317064	-0.631902
H	-1.757158	1.130296	-1.617407
H	-2.829421	2.201354	-0.755152
C	-5.437692	-1.973837	-2.198204
H	-6.288486	-2.469471	-1.736059
H	-4.754964	-2.706554	-2.630672
H	-5.769116	-1.288246	-2.978999
H	-2.475814	-0.664907	0.232681
C	-1.371882	2.012299	1.743584
O	-0.557340	2.418161	2.545609
O	-2.669683	1.796903	2.071268
C	-1.057323	1.671188	0.324183
C	0.025950	2.567538	-0.276511
H	0.550803	3.087981	0.524232
H	-0.451741	3.325476	-0.910834
C	-3.021270	2.066497	3.440916
H	-4.082652	1.839875	3.518546
H	-2.833103	3.112876	3.684634
H	-2.442170	1.433870	4.114877
C	2.085329	2.695134	-1.755631
O	2.322357	2.744702	-2.939226
O	2.724933	3.450786	-0.840088
C	1.058333	1.777199	-1.115765
C	1.752751	0.707806	-0.236565
H	2.532982	0.202661	-0.816164
H	2.251914	1.195826	0.601444
C	3.739348	4.340435	-1.343863
H	4.133559	4.854390	-0.470141
H	3.305786	5.052545	-2.047229
H	4.525764	3.774971	-1.845466
H	0.561870	1.293305	-1.960511
C	1.023478	-0.782917	1.686813
O	2.054729	-0.602612	2.296417
O	-0.038990	-1.438993	2.202931
C	0.747128	-0.301247	0.294874
C	0.225204	-1.363114	-0.675987
H	-0.320852	-0.882913	-1.493201
H	-0.480965	-2.009257	-0.151775
C	0.153649	-2.027853	3.502490
H	-0.809350	-2.455970	3.772826
H	0.457022	-1.268763	4.224289
H	0.913678	-2.808275	3.447629
H	-0.317870	0.536115	0.477344
H	0.030875	-2.786630	-3.015146
H	-3.852147	0.378627	0.444034

Methyl acrylate migration TS with chirality RSS
Standard orientation Energy= -1533.46428290

C	-3.292065	0.769995	-1.516707
O	-3.713909	-0.014717	-2.331988
O	-4.092632	1.505993	-0.716498
C	-1.829177	1.135896	-1.312001
C	-1.556835	2.424237	-2.121844
H	-0.538030	2.777946	-1.946412
H	-2.245162	3.221156	-1.833974
C	-5.509022	1.328991	-0.903295
H	-5.982481	1.991577	-0.181933
H	-5.793946	0.292693	-0.717533
H	-5.797315	1.600043	-1.920010
H	-1.694912	1.356572	-0.250930
C	2.132239	3.281126	0.656105
O	2.228096	3.758889	1.759574
O	2.316030	3.990919	-0.479174
C	1.825574	1.830772	0.340637
C	1.111014	1.127568	1.497533
H	1.709322	1.274498	2.404447
H	0.164730	1.632700	1.700037
C	2.673890	5.376526	-0.307965
H	2.778755	5.775176	-1.314480
H	3.613551	5.462304	0.239294
H	1.892126	5.907115	0.236840
H	2.789434	1.358836	0.119456
C	0.139543	-1.017936	2.468537
O	0.075517	-2.215246	2.652775
O	-0.488667	-0.122217	3.264560
C	0.861153	-0.370601	1.326721
C	1.999136	-1.227619	0.771227
H	1.939813	-2.220890	1.220469
H	2.963479	-0.792330	1.053822
C	-1.248012	-0.674409	4.353741
H	-1.663069	0.181940	4.881068
H	-0.603013	-1.256287	5.013469
H	-2.044649	-1.317345	3.976776
C	3.118417	-2.241867	-1.229019
O	3.071723	-3.402153	-1.552111
O	4.266346	-1.523453	-1.208193
C	1.944673	-1.390900	-0.775151
C	0.605393	-2.008181	-1.217924
H	0.606437	-2.138229	-2.306613
H	0.508801	-3.006325	-0.791373
C	5.463739	-2.236474	-1.574597
H	6.269343	-1.509288	-1.500396
H	5.382682	-2.617983	-2.593186
H	5.634383	-3.070507	-0.892620
H	2.080400	-0.410486	-1.239619
C	-1.747246	-1.946469	-0.301452
O	-1.905846	-3.130101	-0.481874
O	-2.613844	-1.187601	0.414340
C	-0.580970	-1.146766	-0.799274
C	-0.882382	0.006148	-1.759073
H	-1.282906	-0.425083	-2.686051
H	0.075528	0.458346	-2.032405
C	-3.772633	-1.879606	0.917459

H	-4.328131	-1.138310	1.488470
H	-3.469194	-2.711072	1.554565
H	-4.373579	-2.261367	0.090822
H	-0.052765	-0.578920	0.354756
H	-1.671596	2.239673	-3.193701
H	1.250869	1.798762	-0.586292

Methyl acrylate MCR pentamer with the radical on unit 4 with reduced flexibility of 10.4 angstrom
Standard orientation Energy= -1533.50507608

C	4.376531	-1.898872	-0.691344
O	3.917215	-2.609152	-1.554543
O	4.692823	-2.342937	0.541461
C	4.718920	-0.425143	-0.864842
C	6.070641	-0.340464	-1.606205
H	5.986170	-0.759172	-2.612302
H	6.851165	-0.886826	-1.071017
C	4.432927	-3.737696	0.798809
H	4.764210	-3.909245	1.820529
H	4.990567	-4.364437	0.101714
H	3.367082	-3.947114	0.700462
H	4.842850	0.006731	0.131343
C	-5.471583	-1.227917	-0.673778
O	-5.658387	-1.744929	0.400401
O	-6.298352	-1.386492	-1.729495
C	-4.313464	-0.320284	-1.031796
C	-3.269878	-0.228241	0.100077
H	-3.775039	0.127500	1.000591
H	-2.895089	-1.232647	0.311870
C	-7.453364	-2.219290	-1.510328
H	-7.987552	-2.228377	-2.457739
H	-8.079291	-1.802760	-0.719989
H	-7.147823	-3.228567	-1.231528
H	-4.722747	0.669228	-1.255088
C	-2.230957	2.121213	-0.124517
O	-1.327555	2.906413	-0.367426
O	-3.461944	2.530422	0.291481
C	-2.124282	0.674642	-0.243402
C	-0.809404	0.127259	-0.700139
H	-0.394210	0.791611	-1.461536
H	-0.946692	-0.863388	-1.144243
C	-3.627558	3.948267	0.450078
H	-4.653097	4.086042	0.786808
H	-3.462299	4.463363	-0.497805
H	-2.926432	4.335513	1.191199
C	-0.193732	-1.044785	1.435948
O	-0.110150	-2.239755	1.271460
O	-0.733404	-0.485880	2.538145
C	0.259328	0.006282	0.436629
C	1.645202	-0.375787	-0.126374
H	2.263424	-0.735165	0.699424
H	1.531705	-1.209743	-0.823317
C	-1.222039	-1.399983	3.540168
H	-1.592654	-0.772509	4.347516
H	-0.414798	-2.043182	3.892203
H	-2.024469	-2.017717	3.134625
H	0.317235	0.961688	0.958772
C	2.793802	1.852200	0.173724
O	2.925265	1.697774	1.365173
O	3.033024	3.025767	-0.447860
C	2.379088	0.778128	-0.825242
C	3.617364	0.321067	-1.645541
H	4.057984	1.210345	-2.103360
H	3.275747	-0.321278	-2.461813
C	3.439243	4.120459	0.394730
H	3.577332	4.966099	-0.275369
H	4.370348	3.881947	0.910943
H	2.665045	4.338110	1.131381
H	1.714480	1.270948	-1.540985
H	6.385060	0.702521	-1.692150
H	-3.867217	-0.685696	-1.961442

Methyl acrylate MCR pentamer with the radical on unit 4 with reduced flexibility of 10.6 angstrom
Standard orientation Energy= -1533.50477727

C	4.511307	-1.787798	-0.698831
O	4.125401	-2.470023	-1.618622
O	4.797580	-2.285296	0.521298
C	4.786277	-0.291951	-0.775540
C	6.181253	-0.101759	-1.414154
H	6.188736	-0.469343	-2.443516
H	6.949583	-0.637422	-0.851458
C	4.603492	-3.704100	0.688312
H	4.892690	-3.916189	1.715207
H	5.229376	-4.259535	-0.011378
H	3.557497	-3.964472	0.522046
H	4.821377	0.091501	0.247132
C	-5.554806	-1.146425	-0.632596
O	-5.667859	-1.743824	0.409912
O	-6.466646	-1.207417	-1.626764
C	-4.410268	-0.230379	-1.010238
C	-3.254876	-0.289668	0.010961
H	-3.664158	-0.042315	0.994283
H	-2.886947	-1.316915	0.065137
C	-7.621034	-2.032548	-1.377896
H	-8.228941	-1.958469	-2.276791
H	-8.171601	-1.666487	-0.510235
H	-7.319772	-3.065931	-1.201594
H	-4.807435	0.786286	-1.076578
C	-2.219897	2.069986	-0.102991
O	-1.322015	2.869571	-0.316980
O	-3.438804	2.451953	0.371135
C	-2.117750	0.632853	-0.315036
C	-0.798779	0.117564	-0.801455
H	-0.379518	0.834723	-1.510663
H	-0.931257	-0.839924	-1.314020
C	-3.597268	3.856714	0.625702
H	-4.613579	3.972840	0.996835
H	-3.454986	4.432149	-0.290639
H	-2.876800	4.194511	1.372407
C	-0.192517	-1.215330	1.240349
O	-0.118441	-2.390427	0.965313
O	-0.715747	-0.757628	2.395718
C	0.258480	-0.079571	0.336670
C	1.664416	-0.395814	-0.222481
H	2.269749	-0.797755	0.593576
H	1.584715	-1.184470	-0.974839
C	-1.198805	-1.757758	3.315035
H	-1.553806	-1.205555	4.182175
H	-0.392353	-2.436507	3.594661
H	-2.011501	-2.329177	2.864597
H	0.294391	0.830026	0.936394
C	2.716241	1.854589	0.238851
O	2.816215	1.644944	1.425106
O	2.915259	3.068753	-0.314417
C	2.394986	0.813297	-0.826433
C	3.697534	0.438082	-1.590390
H	4.122866	1.362814	-1.988769
H	3.430303	-0.184549	-2.448499
C	3.233978	4.138093	0.595625
H	3.352432	5.022365	-0.026645
H	4.156818	3.920220	1.135288

H	2.423162	4.279466	1.311122
H	1.755030	1.313617	-1.558991
H	6.445349	0.958495	-1.424819
H	-4.072741	-0.497312	-2.015819

Methyl acrylate MCR pentamer with the radical on unit 4 with reduced flexibility of 10.8 angstrom
Standard orientation Energy= -1533.50394042

C	4.648094	-1.683029	-0.653707
O	4.335518	-2.358915	-1.605377
O	4.906194	-2.198493	0.565672
C	4.853423	-0.174439	-0.681497
C	6.279115	0.096491	-1.224669
H	6.371051	-0.246416	-2.258431
H	7.032583	-0.415961	-0.622030
C	4.779803	-3.629293	0.687741
H	5.028170	-3.853998	1.722627
H	5.468777	-4.132286	0.007869
H	3.759143	-3.939755	0.461000
H	4.807568	0.184582	0.349515
C	-5.637872	-1.071365	-0.594146
O	-5.670883	-1.766241	0.392279
O	-6.634690	-1.026169	-1.504131
C	-4.515697	-0.132643	-0.980668
C	-3.256734	-0.345343	-0.110050
H	-3.559333	-0.239075	0.936929
H	-2.912954	-1.374652	-0.235285
C	-7.779579	-1.856847	-1.231344
H	-8.462216	-1.690569	-2.061663
H	-8.244492	-1.567848	-0.287768
H	-7.485482	-2.905910	-1.179586
H	-4.886531	0.889451	-0.868989
C	-2.203452	2.012485	-0.069212
O	-1.305048	2.820163	-0.246615
O	-3.401260	2.364920	0.476280
C	-2.120439	0.594820	-0.397698
C	-0.796906	0.111032	-0.912887
H	-0.377699	0.874017	-1.572840
H	-0.925616	-0.811433	-1.486366
C	-3.534624	3.745446	0.850675
H	-4.536527	3.839608	1.264756
H	-3.416214	4.393185	-0.019595
H	-2.785826	4.013691	1.597687
C	-0.191276	-1.356663	1.032162
O	-0.109804	-2.507506	0.670690
O	-0.721512	-0.989208	2.216434
C	0.256984	-0.154040	0.217197
C	1.680647	-0.416182	-0.332706
H	2.271078	-0.855648	0.475239
H	1.631321	-1.164154	-1.128133
C	-1.204326	-2.057848	3.055443
H	-1.562786	-1.574321	3.961316
H	-0.396983	-2.754269	3.284400
H	-2.014639	-2.594694	2.560412
H	0.274724	0.709949	0.881234
C	2.639706	1.849096	0.257342
O	2.704422	1.602619	1.439115
O	2.800739	3.089562	-0.247163
C	2.414148	0.830975	-0.853245
C	3.776867	0.520673	-1.544601
H	4.181318	1.470288	-1.904632
H	3.585784	-0.097697	-2.425772
C	3.026378	4.141309	0.709975
H	3.131496	5.050352	0.121942
H	3.932411	3.947944	1.286185
H	2.176954	4.221321	1.389221
H	1.806966	1.333488	-1.611708
H	6.491200	1.167993	-1.196223
H	-4.298222	-0.270847	-2.043410

Methyl acrylate MCR pentamer with the radical on unit 4 with reduced flexibility of 11.0 angstrom
Standard orientation Energy= -1533.50216124

C	4.734556	-1.640642	-0.619293
O	4.454662	-2.311168	-1.584888
O	4.965866	-2.164191	0.601941
C	4.922159	-0.129573	-0.628540
C	6.366074	0.163397	-1.126112
H	6.495497	-0.172628	-2.157992
H	7.103144	-0.344542	-0.500215
C	4.850792	-3.597184	0.708663
H	5.074192	-3.828293	1.747818
H	5.562338	-4.087488	0.042896
H	3.839542	-3.915664	0.452829
H	4.844327	0.217970	0.404493
C	-5.720178	-1.031496	-0.531963
O	-5.674176	-1.788565	0.407300
O	-6.791236	-0.926039	-1.347772
C	-4.630049	-0.070183	-0.950812
C	-3.289201	-0.375395	-0.236497
H	-3.490247	-0.376177	0.841365
H	-2.981620	-1.392533	-0.490537
C	-7.915652	-1.768150	-1.028926
H	-8.665911	-1.547672	-1.784955
H	-8.294800	-1.537356	-0.032435
H	-7.627638	-2.819435	-1.068214
H	-4.968784	0.939712	-0.708526
C	-2.203160	1.973846	-0.143276
O	-1.299224	2.774801	-0.321927
O	-3.379383	2.327250	0.446388
C	-2.144564	0.563697	-0.512746
C	-0.813316	0.080535	-1.022796
H	-0.397911	0.843181	-1.686699
H	-0.937354	-0.843712	-1.593938
C	-3.481073	3.698595	0.863102
H	-4.468893	3.795021	1.309308
H	-3.378530	4.368948	0.008147
H	-2.706822	3.934657	1.594796
C	-0.191769	-1.374632	0.924595
O	-0.044363	-2.529132	0.598072
O	-0.805391	-1.002111	2.067021
C	0.250808	-0.172158	0.106201
C	1.685474	-0.423095	-0.432588
H	2.248684	-0.922357	0.359386
H	1.645682	-1.122595	-1.271521
C	-1.295892	-2.070390	2.902026
H	-1.722563	-1.582014	3.775181
H	-0.477908	-2.730816	3.191656
H	-2.056982	-2.647537	2.375187
H	0.255579	0.693512	0.767862
C	2.641042	1.803019	0.302400
O	2.647405	1.503667	1.473626
O	2.840239	3.062917	-0.137196
C	2.457044	0.838560	-0.862177
C	3.852423	0.557691	-1.510907
H	4.248392	1.521143	-1.842454
H	3.700275	-0.049083	-2.407563
C	3.035980	4.066677	0.876259
H	3.178070	5.000000	0.335935
H	3.913237	3.834603	1.481970
H	2.158637	4.127810	1.521144
H	1.886741	1.380747	-1.622581

H	6.563070	1.237274	-1.084214
H	-4.525633	-0.112820	-2.038302

Methyl acrylate MCR pentamer with the radical on unit 4 with reduced flexibility of 11.2 angstrom
Standard orientation Energy= -1533.50079612

C	4.532454	-2.048787	-0.312707
O	3.912082	-2.687475	-1.129965
O	4.993793	-2.575431	0.840117
C	4.941173	-0.589261	-0.452368
C	6.385378	-0.535936	-1.028309
H	6.415301	-0.943150	-2.042569
H	7.074099	-1.107289	-0.403157
C	4.712293	-3.970708	1.059993
H	5.155199	-4.206995	2.024905
H	5.157954	-4.579176	0.271732
H	3.635436	-4.142379	1.080620
H	4.976211	-0.172921	0.557831
C	-5.816328	-0.881236	0.916843
O	-5.842965	-0.018865	1.760671
O	-6.762229	-1.840576	0.807655
C	-4.770341	-1.031302	-0.165431
C	-3.453835	-0.295183	0.196147
H	-3.726187	0.735082	0.454520
H	-3.030835	-0.736693	1.100199
C	-7.844751	-1.771171	1.754992
H	-8.493189	-2.611191	1.515943
H	-8.383432	-0.828189	1.651050
H	-7.465348	-1.856134	2.774102
H	-5.194463	-0.614810	-1.082878
C	-2.610324	0.425857	-2.139333
O	-1.794018	0.487878	-3.045295
O	-3.829269	1.030779	-2.220102
C	-2.395923	-0.271084	-0.875041
C	-1.019639	-0.852503	-0.677534
H	-0.715254	-1.350222	-1.602474
H	-1.034260	-1.587532	0.129694
C	-4.086320	1.756825	-3.432732
H	-5.085295	2.172629	-3.317077
H	-4.044926	1.090709	-4.296199
H	-3.353386	2.554336	-3.565136
C	-0.192641	0.798920	1.020883
O	-0.526803	0.157296	1.987074
O	-0.023646	2.138967	1.044784
C	0.074359	0.218704	-0.361064
C	1.518805	-0.362283	-0.464996
H	1.737691	-0.967228	0.418382
H	1.539590	-1.034221	-1.326118
C	-0.198647	2.765780	2.331533
H	-0.051757	3.829926	2.157959
H	0.538309	2.379777	3.036726
H	-1.201815	2.575261	2.714205
H	-0.018563	1.021177	-1.095359
C	2.957724	1.473335	0.584765
O	2.841853	1.063284	1.715112
O	3.486465	2.682466	0.291225
C	2.614357	0.707242	-0.683490
C	3.948841	0.202617	-1.330566
H	4.470107	1.092414	-1.692568
H	3.695205	-0.398518	-2.207472
C	3.921751	3.475444	1.410502
H	4.327193	4.388448	0.979811
H	4.686757	2.947114	1.981399
H	3.080944	3.703674	2.066579
H	2.225341	1.442939	-1.394256
H	6.730970	0.499763	-1.064263

H	-4.600021	-2.094807	-0.348864

Methyl acrylate MCR pentamer with the radical on unit 4 with reduced flexibility of 11.4 angstrom
Standard orientation Energy= -1533.49793819

C	4.729164	-1.828697	-0.636756
O	4.096074	-2.397139	-1.495043
O	5.302767	-2.468395	0.402934
C	5.026970	-0.336589	-0.595607
C	6.458880	-0.096787	-1.171542
H	6.505291	-0.370655	-2.228999
H	7.195586	-0.687188	-0.624390
C	5.121794	-3.896441	0.449430
H	5.646144	-4.225963	1.343618
H	5.545261	-4.364578	-0.440365
H	4.061316	-4.142569	0.514925
H	5.056121	-0.053486	0.459888
C	-5.887789	-1.102027	0.658979
O	-5.826368	-0.587305	1.748950
O	-6.904465	-1.905771	0.275596
C	-4.888786	-0.925966	-0.462363
C	-3.501486	-0.463857	0.071519
H	-3.685684	0.413607	0.704872
H	-3.101801	-1.232726	0.735642
C	-7.951327	-2.105946	1.244269
H	-8.666593	-2.770398	0.764553
H	-8.421588	-1.155438	1.500321
H	-7.550136	-2.562262	2.150105
H	-5.297590	-0.177285	-1.145088
C	-2.653502	0.950693	-1.928545
O	-1.845919	1.269276	-2.786963
O	-3.852469	1.584931	-1.793349
C	-2.444873	-0.110149	-0.946769
C	-1.061883	-0.721487	-0.944641
H	-0.779505	-0.936190	-1.979522
H	-1.065350	-1.656428	-0.381135
C	-4.097280	2.658902	-2.715858
H	-5.080339	3.047044	-2.456637
H	-4.087416	2.292841	-3.743867
H	-3.339119	3.436362	-2.609137
C	-0.194585	0.369308	1.135657
O	-0.446159	-0.538797	1.889860
O	-0.123031	1.659897	1.528124
C	0.052860	0.213491	-0.358689
C	1.500294	-0.310469	-0.643352
H	1.746684	-1.113886	0.054283
H	1.502651	-0.746928	-1.645265
C	-0.290198	1.890339	2.941640
H	-0.235242	2.969780	3.068069
H	0.505974	1.391770	3.495672
H	-1.257147	1.514351	3.277361
H	-0.050900	1.191936	-0.830911
C	2.895184	1.294335	0.781719
O	2.809199	0.647213	1.798092
O	3.356650	2.564858	0.754773
C	2.584675	0.797824	-0.621157
C	3.947214	0.480628	-1.343418
H	4.389737	1.449685	-1.587545
H	3.720603	-0.015431	-2.290515
C	3.750382	3.125624	2.020429
H	4.106366	4.128755	1.795851
H	4.542680	2.529442	2.475503
H	2.898509	3.166262	2.700211
H	2.182472	1.654543	-1.170414
H	6.721610	0.959373	-1.078173
H	-4.813084	-1.860846	-1.022378

Methyl acrylate MCR pentamer with the radical on unit 4 with reduced flexibility of 11.6 angstrom

Standard orientation Energy= -1533.49371616

C	4.885391	-1.592166	-0.859281
O	4.214577	-2.130356	-1.708200
O	5.574995	-2.273513	0.078448
C	5.101780	-0.091981	-0.719309
C	6.511913	0.274478	-1.302056
H	6.555448	0.077000	-2.376450
H	7.287407	-0.309631	-0.805048
C	5.463058	-3.708624	0.032026
H	6.078594	-4.073969	0.851061
H	5.826727	-4.089965	-0.923219
H	4.424432	-4.011881	0.168848
H	5.150149	0.113192	0.353317
C	-5.975638	-1.266671	0.366661
O	-5.829489	-1.201983	1.563024
O	-7.054040	-1.833854	-0.218101
C	-5.028874	-0.713031	-0.673512
C	-3.581250	-0.558847	-0.102835
H	-3.675402	0.008751	0.833170
H	-3.214841	-1.544425	0.192539
C	-8.059412	-2.348802	0.675505
H	-8.834738	-2.760069	0.032896
H	-8.461424	-1.549316	1.299493
H	-7.638623	-3.125212	1.315678
H	-5.417324	0.259746	-0.982364
C	-2.719878	1.426986	-1.544826
O	-1.923809	1.980844	-2.286386
O	-3.893186	2.015584	-1.177725
C	-2.520047	0.105804	-0.954322
C	-1.135040	-0.482495	-1.159171
H	-0.894537	-0.416082	-2.225229
H	-1.130217	-1.534206	-0.867475
C	-4.123306	3.330923	-1.708639
H	-5.086360	3.641986	-1.308544
H	-4.150025	3.306394	-2.799276
H	-3.337059	4.016391	-1.388445
C	-0.182538	0.016448	1.097884
O	-0.389936	-1.062033	1.598212
O	-0.138269	1.165799	1.807015
C	0.023820	0.255853	-0.391733
C	1.459825	-0.192205	-0.845723
H	1.715507	-1.146607	-0.381518
H	1.425820	-0.363126	-1.925110
C	-0.274962	1.020201	3.234809
H	-0.250355	2.032450	3.633619
H	0.550969	0.426272	3.627904
H	-1.220253	0.535046	3.480180
H	-0.076097	1.324131	-0.589700
C	2.876234	1.050935	0.889621
O	2.804935	0.195011	1.738827
O	3.318459	2.303736	1.142055
C	2.567406	0.870053	-0.586789
C	3.940086	0.709061	-1.364560
H	4.310002	1.724479	-1.526322
H	3.722876	0.286194	-2.348392
C	3.707002	2.579626	2.500209
H	4.052846	3.611046	2.499875
H	4.505221	1.906544	2.816592
H	2.854627	2.463303	3.170539
H	2.172641	1.827970	-0.937541
H	6.713100	1.335457	-1.138078
H	-5.046428	-1.359012	-1.554091

Methyl acrylate MCR pentamer with the radical on unit 4 with reduced flexibility of 11.8 angstrom

Standard orientation Energy= -1533.49057776

C	5.605379	-0.551542	-0.672643
O	5.623474	-1.471366	-1.452490
O	6.023981	-0.658742	0.608421
C	5.191085	0.873745	-1.027101
C	6.370629	1.448415	-1.889998
H	6.487713	0.875799	-2.812191
H	7.302808	1.398031	-1.323800
C	6.462817	-1.972698	1.008468
H	6.783150	-1.870242	2.043399
H	7.291414	-2.305428	0.382229
H	5.639144	-2.682594	0.925669
H	5.146848	1.455603	-0.103450
C	-6.183557	-1.052510	-0.461702
O	-5.892086	-2.042352	0.164629
O	-7.389995	-0.871104	-1.041843
C	-5.295188	0.150832	-0.679627
C	-3.779839	-0.232455	-0.542018
H	-3.684206	-0.765655	0.414369
H	-3.541366	-0.974851	-1.307401
C	-8.348093	-1.930124	-0.852078
H	-9.241733	-1.610494	-1.383448
H	-8.560139	-2.067244	0.209186
H	-7.968605	-2.865461	-1.265260
H	-5.563865	0.896238	0.071549
C	-2.786019	2.045919	0.245351
O	-1.993740	2.972640	0.188972
O	-3.830251	2.040958	1.120031
C	-2.712259	0.845406	-0.584166
C	-1.433003	0.712668	-1.399027
H	-1.292111	1.646061	-1.954483
H	-1.535354	-0.103848	-2.116674
C	-3.927771	3.188425	1.980205
H	-4.798548	3.008625	2.607631
H	-4.058378	4.098943	1.393181
H	-3.029374	3.285895	2.591589
C	-0.246224	-0.875662	0.104819
O	-0.409255	-1.915629	-0.485101
O	-0.228499	-0.781380	1.451313
C	-0.102820	0.481658	-0.574606
C	1.191318	0.520584	-1.465895
H	1.315260	-0.455033	-1.942975
H	1.032552	1.251050	-2.265558
C	-0.349842	-2.031459	2.161197
H	-0.348418	-1.767283	3.216811
H	0.494572	-2.678145	1.920353
H	-1.279866	-2.534035	1.893695
H	-0.043902	1.260908	0.186109
C	2.763315	0.054660	0.483863
O	2.778862	-1.153303	0.501269
O	2.975816	0.815003	1.578852
C	2.526219	0.918782	-0.745450
C	3.778507	0.869132	-1.708641
H	3.703923	1.737630	-2.369851
H	3.712423	-0.020299	-2.340346
C	3.237441	0.107962	2.805428
H	3.354257	0.878469	3.564486
H	4.150666	-0.479744	2.711568
H	2.400663	-0.546654	3.049463
H	2.425772	1.945383	-0.384216
H	6.177482	2.493329	-2.145163
H	-5.511804	0.586808	-1.657156

Methyl acrylate migration TS with reduced flexibility of 8.4 angstrom

Standard orientation Energy= -1533.47697770

C	4.334639	-0.523438	0.177892
O	4.594545	-0.174192	1.304633
O	4.771005	0.127444	-0.919978
C	3.499234	-1.745100	-0.177399
C	4.191419	-2.996721	0.396273
H	4.256495	-2.935268	1.484757
H	5.204572	-3.101095	-0.000410
C	5.558065	1.313539	-0.681409
H	5.842371	1.673992	-1.667424
H	6.440777	1.070790	-0.088879
H	4.962845	2.061319	-0.155853
H	3.461545	-1.825481	-1.265752
C	-4.718392	-0.237270	-0.653763
O	-4.585495	-0.883299	-1.664288
O	-5.889194	0.323573	-0.279780
C	-3.636852	0.049143	0.366602
C	-2.246042	-0.407564	-0.108851
H	-2.292755	-1.469088	-0.352821
H	-2.002349	0.114003	-1.037900
C	-7.007711	0.101310	-1.159682
H	-7.845274	0.621709	-0.700564
H	-7.219045	-0.965398	-1.246347
H	-6.799672	0.506979	-2.150602
H	-3.919270	-0.466624	1.290179
C	-1.148312	-0.977689	2.156212
O	-0.563515	-0.697428	3.181959
O	-1.848911	-2.130438	2.007837
C	-1.140975	-0.155540	0.911970
C	-0.695590	1.287057	1.108584
H	-0.173168	1.371226	2.063999
H	-1.568529	1.945816	1.154502
C	-1.846158	-3.015593	3.142282
H	-2.445404	-3.872831	2.842676
H	-2.284798	-2.524799	4.012286
H	-0.828113	-3.324191	3.384246
C	0.607839	3.230520	0.163551
O	1.720751	3.658126	0.353509
O	-0.484858	4.023678	0.093656
C	0.236263	1.771166	-0.033174
C	1.500190	0.896240	-0.121057
H	2.130807	1.271362	-0.931709
H	2.066108	1.006247	0.806362
C	-0.254757	5.434862	0.266329
H	-1.233804	5.901215	0.180702
H	0.417243	5.808363	-0.507454
H	0.181437	5.633328	1.246215
H	-0.303052	1.717704	-0.983385
C	0.771150	-0.902347	-1.746196
O	0.483429	-0.078877	-2.591019
O	0.697214	-2.236174	-1.972039
C	1.186247	-0.577789	-0.349530
C	2.050034	-1.606553	0.373336
H	1.579006	-2.588193	0.294935
H	2.106726	-1.348774	1.434805
C	0.228659	-2.626456	-3.276799
H	0.244033	-3.714258	-3.276329
H	0.886555	-2.232552	-4.052504
H	-0.784225	-2.257314	-3.443483
H	-0.020135	-0.632958	0.296730
H	3.627579	-3.893542	0.128182
H	-3.661989	1.118338	0.593919

Methyl acrylate migration TS with reduced flexibility of 8.6 angstrom

Standard orientation Energy= -1533.47558483

C	4.361687	-0.404375	0.189527
O	4.581981	-0.062383	1.326949
O	4.791230	0.283079	-0.888354
C	3.598546	-1.662364	-0.201762
C	4.405476	-2.882159	0.302808
H	4.495892	-2.860445	1.390923
H	5.410765	-2.884254	-0.125662
C	5.515489	1.501420	-0.614528
H	5.811953	1.885295	-1.588066
H	6.390378	1.292958	0.002103
H	4.870025	2.214917	-0.100709
H	3.549683	-1.702357	-1.292112
C	-4.751346	-0.179127	-0.662494
O	-4.646582	-0.871791	-1.644889
O	-5.894522	0.454085	-0.319194
C	-3.662830	0.094391	0.353659
C	-2.283198	-0.427595	-0.111099
H	-2.376901	-1.487860	-0.345619
H	-2.017989	0.073582	-1.045447
C	-7.019182	0.250031	-1.195643
H	-7.832001	0.829455	-0.763525
H	-7.282219	-0.807783	-1.239126
H	-6.787664	0.603230	-2.201334
H	-3.964221	-0.389810	1.288456
C	-1.190264	-1.047781	2.145303
O	-0.586949	-0.796405	3.167600
O	-1.932947	-2.172966	1.994773
C	-1.162685	-0.215860	0.906355
C	-0.695588	1.217347	1.120317
H	-0.158633	1.280761	2.069304
H	-1.559657	1.885497	1.188862
C	-1.951249	-3.067082	3.122013
H	-2.583790	-3.899675	2.821387
H	-2.363592	-2.568116	4.000184
H	-0.942670	-3.414027	3.351350
C	0.588641	3.168031	0.160068
O	1.700789	3.605829	0.331129
O	-0.511177	3.952328	0.103209
C	0.226075	1.704778	-0.026729
C	1.496405	0.841002	-0.112162
H	2.121879	1.218002	-0.926278
H	2.062030	0.966743	0.813245
C	-0.289661	5.366079	0.265599
H	-1.273575	5.824307	0.192824
H	0.367401	5.741105	-0.520190
H	0.159889	5.572748	1.237718
H	-0.316519	1.643208	-0.974499
C	0.789678	-0.982169	-1.719700
O	0.458581	-0.170095	-2.559573
O	0.761909	-2.317374	-1.945681
C	1.212163	-0.641917	-0.327683
C	2.144912	-1.634459	0.370356
H	1.736477	-2.641938	0.275263
H	2.197424	-1.394349	1.435987
C	0.289926	-2.724260	-3.244052
H	0.341500	-3.810945	-3.243182
H	0.924399	-2.309554	-4.028406
H	-0.736577	-2.388922	-3.398297
H	-0.019243	-0.708607	0.311379
H	3.907718	-3.809769	0.009732
H	-3.648633	1.168552	0.555057

Methyl acrylate migration TS with reduced flexibility of 8.8 angstrom

Standard orientation Energy= -1533.47265775

C	4.387699	-0.307632	0.214209
O	4.571477	0.017912	1.362943
O	4.810605	0.419693	-0.839788
C	3.691021	-1.590742	-0.218801
C	4.600758	-2.774577	0.217074
H	4.716923	-2.791878	1.302685
H	5.590104	-2.678921	-0.236317
C	5.475543	1.662169	-0.525686
H	5.784840	2.073396	-1.483971
H	6.339343	1.479868	0.114351
H	4.784955	2.339923	-0.022197
H	3.631461	-1.589539	-1.309508
C	-4.802445	-0.130671	-0.658676
O	-4.725363	-0.854631	-1.620704
O	-5.918988	0.558871	-0.336281
C	-3.706613	0.125235	0.353738
C	-2.333314	-0.433571	-0.117976
H	-2.460720	-1.488622	-0.359064
H	-2.061452	0.065338	-1.051683
C	-7.050659	0.374358	-1.208045
H	-7.839409	0.999113	-0.794895
H	-7.356484	-0.672728	-1.220108
H	-6.804492	0.687348	-2.223518
H	-4.014005	-0.351130	1.290667
C	-1.232881	-1.105186	2.122260
O	-0.613721	-0.880416	3.141004
O	-2.008747	-2.206785	1.968456
C	-1.191733	-0.262520	0.889472
C	-0.701733	1.160600	1.118152
H	-0.160778	1.207010	2.065872
H	-1.556252	1.839822	1.195459
C	-2.041654	-3.111251	3.087089
H	-2.700661	-3.922390	2.784696
H	-2.430904	-2.609541	3.974186
H	-1.041178	-3.489064	3.302741
C	0.579554	3.111666	0.151367
O	1.690233	3.556344	0.315190
O	-0.523995	3.890551	0.093575
C	0.223557	1.645333	-0.026399
C	1.498964	0.789223	-0.096386
H	2.130094	1.169285	-0.905114
H	2.053683	0.925685	0.833961
C	-0.308422	5.306394	0.245065
H	-1.294773	5.759510	0.173214
H	0.343553	5.678943	-0.546128
H	0.144205	5.522038	1.213797
H	-0.313267	1.575153	-0.976758
C	0.819749	-1.049804	-1.698554
O	0.450174	-0.246939	-2.531038
O	0.838551	-2.383682	-1.931241
C	1.243129	-0.700269	-0.307615
C	2.236203	-1.661198	0.365856
H	1.883162	-2.686942	0.250188
H	2.283833	-1.441499	1.435891
C	0.369498	-2.801252	-3.227183
H	0.457554	-3.885584	-3.231165
H	0.982984	-2.362567	-4.015199
H	-0.668853	-2.499849	-3.371825
H	-0.021237	-0.773124	0.313806
H	4.165402	-3.723295	-0.106109
H	-3.666758	1.199065	0.551345

Methyl acrylate migration TS with reduced flexibility of 9.0 angstrom

Standard orientation Energy= -1533.46796199

C	4.414736	-0.226907	0.242335
O	4.569219	0.079324	1.400704
O	4.823606	0.540293	-0.788714
C	3.780501	-1.528880	-0.229237
C	4.781991	-2.671519	0.147579
H	4.923103	-2.719566	1.229091
H	5.749362	-2.486456	-0.324325
C	5.430078	1.802863	-0.437497
H	5.746834	2.242105	-1.380829
H	6.283831	1.644347	0.221979
H	4.699202	2.443666	0.057491
H	3.711276	-1.492747	-1.318872
C	-4.864635	-0.093872	-0.647531
O	-4.811621	-0.838700	-1.595017
O	-5.956017	0.642515	-0.342595
C	-3.765028	0.139334	0.365682
C	-2.390998	-0.431963	-0.124718
H	-2.539659	-1.479378	-0.385382
H	-2.122660	0.080574	-1.052170
C	-7.092166	0.480058	-1.212997
H	-7.858560	1.141586	-0.815306
H	-7.435474	-0.555372	-1.203272
H	-6.833087	0.761954	-2.234333
H	-4.071804	-0.350991	1.295554
C	-1.273224	-1.156158	2.092924
O	-0.640614	-0.956037	3.108255
O	-2.074264	-2.238453	1.934057
C	-1.225059	-0.300564	0.867988
C	-0.712720	1.112216	1.110313
H	-0.174896	1.144081	2.060422
H	-1.557949	1.802923	1.187637
C	-2.115811	-3.155482	3.042134
H	-2.794927	-3.948656	2.736615
H	-2.485585	-2.656375	3.938978
H	-1.121582	-3.557076	3.243211
C	0.573798	3.061515	0.144231
O	1.682000	3.512332	0.309334
O	-0.532375	3.835708	0.076419
C	0.224813	1.592240	-0.026266
C	1.505764	0.743742	-0.074321
H	2.147576	1.130423	-0.871861
H	2.043761	0.886635	0.864799
C	-0.322901	5.253467	0.217896
H	-1.310754	5.702116	0.138971
H	0.330494	5.622569	-0.573747
H	0.125296	5.478294	1.186599
H	-0.300466	1.513249	-0.982297
C	0.859447	-1.103545	-1.682030
O	0.458808	-0.307589	-2.506542
O	0.920537	-2.434021	-1.925082
C	1.279271	-0.750434	-0.289451
C	2.325335	-1.684046	0.360488
H	2.019875	-2.721923	0.222554
H	2.367780	-1.485889	1.434711
C	0.458453	-2.856989	-3.221660
H	0.579742	-3.938052	-3.233526
H	1.054396	-2.394400	-4.009479
H	-0.589275	-2.586568	-3.359815
H	-0.023084	-0.828561	0.308832
H	4.404824	-3.635015	-0.203501
H	-3.711701	1.209060	0.579558

Methyl acrylate migration TS with reduced flexibility of 9.2 angstrom

Standard orientation Energy= -1533.46134338

C	4.446932	-0.158294	0.264922
O	4.579843	0.134865	1.429370
O	4.836563	0.639962	-0.750045
C	3.868087	-1.476337	-0.232639
C	4.950523	-2.574549	0.099299
H	5.116463	-2.639588	1.176111
H	5.891257	-2.313010	-0.389116
C	5.390653	1.919428	-0.373470
H	5.706420	2.381654	-1.306119
H	6.238719	1.783880	0.298305
H	4.628136	2.526640	0.116133
H	3.788900	-1.417481	-1.320623
C	-4.932330	-0.070247	-0.639504
O	-4.895364	-0.827968	-1.577384
O	-6.002608	0.703397	-0.352296
C	-3.835609	0.139181	0.381328
C	-2.453120	-0.427322	-0.129972
H	-2.613229	-1.465523	-0.418603
H	-2.192373	0.109328	-1.046047
C	-7.137595	0.562902	-1.228151
H	-7.886234	1.252633	-0.844830
H	-7.511886	-0.461547	-1.206476
H	-6.863500	0.822557	-2.251446
H	-4.140522	-0.378096	1.296982
C	-1.314374	-1.205487	2.061514
O	-0.672246	-1.028778	3.074963
O	-2.132747	-2.272986	1.893246
C	-1.263293	-0.333320	0.847357
C	-0.731130	1.069137	1.105269
H	-0.202481	1.087276	2.060879
H	-1.568088	1.770446	1.177154
C	-2.178896	-3.206067	2.987637
H	-2.871687	-3.984736	2.675588
H	-2.534387	-2.715489	3.894904
H	-1.188959	-3.624781	3.174504
C	0.562578	3.019375	0.148495
O	1.666155	3.478895	0.321360
O	-0.547601	3.786173	0.064918
C	0.225085	1.546446	-0.017522
C	1.513969	0.708798	-0.038957
H	2.169539	1.108390	-0.819236
H	2.029686	0.853630	0.912339
C	-0.348347	5.206127	0.198547
H	-1.338214	5.647945	0.107237
H	0.310136	5.573945	-0.589471
H	0.089270	5.440455	1.169835
H	-0.283108	1.458254	-0.981921
C	0.911160	-1.138419	-1.665578
O	0.491346	-0.345868	-2.483583
O	1.007379	-2.464262	-1.921121
C	1.320836	-0.788107	-0.268029
C	2.413254	-1.698332	0.360439
H	2.145543	-2.743696	0.203756
H	2.453213	-1.518510	1.437707
C	0.558769	-2.886777	-3.222434
H	0.707063	-3.964324	-3.243805
H	1.144497	-2.402343	-4.004776
H	-0.495087	-2.641332	-3.360730
H	-0.024411	-0.875543	0.302590
H	4.625220	-3.549671	-0.270576
H	-3.778581	1.202381	0.623032

Methyl acrylate migration TS with reduced flexibility of 9.4 angstrom

Standard orientation Energy= -1533.45275049

C	4.482936	-0.099402	0.282910
O	4.598345	0.181587	1.452242
O	4.851436	0.726463	-0.718027
C	3.954437	-1.429784	-0.236737
C	5.108359	-2.484066	0.057599
H	5.296727	-2.560168	1.129667
H	6.020169	-2.155352	-0.444064
C	5.358022	2.019121	-0.319567
H	5.667601	2.503468	-1.243043
H	6.203193	1.903659	0.359552
H	4.569981	2.593853	0.168816
H	3.866261	-1.353127	-1.322873
C	-5.004236	-0.057875	-0.628885
O	-4.984532	-0.836331	-1.550120
O	-6.048052	0.761157	-0.371723
C	-3.914410	0.129453	0.402805
C	-2.518551	-0.422282	-0.129892
H	-2.684758	-1.450203	-0.449666
H	-2.267934	0.143341	-1.031437
C	-7.178042	0.641850	-1.257315
H	-7.905070	1.366794	-0.898162
H	-7.589514	-0.367656	-1.216993
H	-6.883550	0.867990	-2.282875
H	-4.215796	-0.421283	1.299762
C	-1.354779	-1.253209	2.032108
O	-0.706320	-1.097313	3.044745
O	-2.183957	-2.310145	1.852097
C	-1.304723	-0.362726	0.830506
C	-0.752954	1.029032	1.103714
H	-0.235819	1.033629	2.065771
H	-1.581493	1.740995	1.168470
C	-2.231383	-3.260597	2.931319
H	-2.932800	-4.028092	2.610972
H	-2.576865	-2.782024	3.848814
H	-1.243908	-3.690747	3.104839
C	0.548517	2.981381	0.160450
O	1.646161	3.450536	0.345504
O	-0.565785	3.739679	0.057149
C	0.224436	1.504602	-0.002841
C	1.522257	0.679583	0.002952
H	2.192621	1.097592	-0.755333
H	2.012561	0.821105	0.968181
C	-0.378461	5.161806	0.184363
H	-1.370137	5.595875	0.076906
H	0.287944	5.529404	-0.597073
H	0.044328	5.405436	1.159923
H	-0.264500	1.407790	-0.976292
C	0.967169	-1.159134	-1.652698
O	0.536135	-0.366184	-2.464298
O	1.091268	-2.479623	-1.922810
C	1.365349	-0.817569	-0.248821
C	2.499517	-1.707952	0.357833
H	2.262813	-2.757592	0.182498
H	2.538739	-1.546523	1.437748
C	0.657254	-2.896182	-3.230817
H	0.826734	-3.970320	-3.263421
H	1.236905	-2.391689	-4.004974
H	-0.400574	-2.669914	-3.371655
H	-0.025723	-0.918406	0.295173
H	4.829635	-3.467071	-0.328429
H	-3.859624	1.184317	0.677199

Methyl acrylate migration TS with reduced flexibility of 9.6 angstrom

Standard orientation Energy= -1533.44221699

C	-4.462501	-0.018732	-0.189443
O	-4.607625	0.190790	-1.370391
O	-4.752000	0.889139	0.765681
C	-3.980266	-1.337375	0.399481
C	-5.217653	-2.342620	0.231624
H	-5.464473	-2.476726	-0.822420
H	-6.076485	-1.917515	0.753255
C	-5.209062	2.177578	0.298359
H	-5.463121	2.735309	1.197051
H	-6.081627	2.061093	-0.344942
H	-4.411081	2.679020	-0.250821
H	-3.842915	-1.193901	1.473508
C	5.094331	-0.052472	0.444002
O	5.730334	-1.029568	0.758158
O	5.214028	1.142693	1.063269
C	4.052638	-0.007106	-0.644207
C	2.636042	-0.485622	-0.007754
H	2.803343	-1.488172	0.386870
H	2.436562	0.170580	0.843601
C	6.148578	1.196373	2.157778
H	6.105995	2.219081	2.525649
H	7.154067	0.953114	1.811877
H	5.859400	0.493828	2.940572
H	4.342547	-0.701190	-1.432190
C	1.365099	-1.471414	-2.042070
O	0.695704	-1.372940	-3.047923
O	2.171829	-2.531048	-1.800001
C	1.369723	-0.491755	-0.909319
C	0.810757	0.879368	-1.257306
H	0.269812	0.821577	-2.204440
H	1.638869	1.582506	-1.388731
C	2.180212	-3.558867	-2.807231
H	2.876089	-4.312684	-2.445503
H	2.514054	-3.154479	-3.763778
H	1.181978	-3.982206	-2.928238
C	-0.436756	2.907832	-0.401524
O	-1.531413	3.389970	-0.571826
O	0.695109	3.645178	-0.385064
C	-0.137614	1.435259	-0.161173
C	-1.455458	0.643950	-0.089902
H	-2.100892	1.135164	0.646383
H	-1.960620	0.732284	-1.053945
C	0.532619	5.061688	-0.587078
H	1.536651	5.477870	-0.542244
H	-0.095102	5.488343	0.196383
H	0.077363	5.258938	-1.558484
H	0.369933	1.385029	0.806233
C	-0.908680	-1.098530	1.674503
O	-0.387406	-0.280635	2.404788
O	-1.110921	-2.382591	2.052317
C	-1.348789	-0.836093	0.266258
C	-2.553411	-1.717601	-0.229936
H	-2.351912	-2.761757	0.008925
H	-2.630851	-1.628053	-1.315879
C	-0.653159	-2.728950	3.372053
H	-0.896867	-3.781933	3.496013
H	-1.161961	-2.125135	4.124613
H	0.423199	-2.572079	3.456281
H	0.063621	-1.006038	-0.300110
H	-4.979100	-3.312389	0.673301
H	3.962513	1.003138	-1.043540

Methyl acrylate tetramer unsaturated (macromonomer)

Standard orientation Energy= -1304.97263480

C	0.100101	2.298531	-0.423705
O	-0.408135	2.675593	-1.449817
O	0.950522	3.063728	0.303663
C	-0.060790	0.913835	0.187594
C	-1.523775	0.433365	0.137264
H	-1.943940	0.673985	-0.842226
C	1.235812	4.368177	-0.235688
H	1.923377	4.830640	0.469461
H	0.320084	4.955034	-0.320228
H	1.695432	4.281475	-1.221306
H	0.272778	0.975725	1.228987
C	3.842486	-1.527768	1.869226
H	4.635932	-1.026263	2.406607
H	3.219798	-2.220287	2.426602
C	4.510295	-0.379901	-0.207946
O	4.399351	-0.178400	-1.396765
O	5.454339	0.216391	0.550057
C	3.630661	-1.311228	0.566937
C	2.526141	-2.005302	-0.204482
H	2.737089	-1.959865	-1.273776
H	2.516421	-3.059086	0.087335
C	6.340480	1.113836	-0.142665
H	7.022065	1.490375	0.616934
H	5.778329	1.932143	-0.594579
H	6.888519	0.583032	-0.922260
C	0.109745	-2.417732	-0.488728
O	-0.229666	-2.516262	-1.641766
O	-0.320764	-3.257643	0.483004
C	1.109019	-1.408301	0.058353
C	0.931238	-0.011226	-0.569172
H	0.617511	-0.122380	-1.610276
H	1.903094	0.485529	-0.590609
C	-1.206807	-4.311398	0.057041
H	-1.443752	-4.873720	0.957627
H	-0.714076	-4.950383	-0.677059
H	-2.112229	-3.893255	-0.384733
H	0.968155	-1.357924	1.141417
H	-1.530388	-0.656974	0.224052
H	-7.298050	-1.489637	-0.580678
H	-6.109161	-0.897693	-1.783296
C	-6.602328	-0.692011	-0.832329
H	-7.123343	0.264144	-0.900110
O	-5.641135	-0.668267	0.239395
C	-4.660265	0.261192	0.134670
O	-4.575077	1.019687	-0.799027
C	-3.749506	0.197506	1.345450
H	-3.553530	-0.859361	1.551387
C	-2.427261	0.984766	1.262184
H	-1.917678	0.771007	2.212235
C	-2.655716	2.503392	1.200384
H	-3.336385	2.819816	1.997386
H	-1.720843	3.050467	1.345106
H	-3.085624	2.801307	0.243715
H	-4.342241	0.555134	2.195997

Methyl acrylate macromonomer propagation TS

Standard orientation Energy= -1612.10839915

C	1.922094	2.656565	-0.188520
O	2.734760	3.023469	0.624065
O	1.275658	3.505144	-1.021882
C	1.440772	1.223751	-0.370496
C	2.601953	0.216976	-0.272127
H	3.240923	0.506053	0.567456
C	1.599534	4.901097	-0.872416
H	0.995466	5.420498	-1.613274

H	2.661618	5.067789	-1.056931
H	1.353718	5.243864	0.133601
H	0.974909	1.157000	-1.359511
C	-3.084169	0.415574	-1.049312
H	-3.626810	1.143888	-1.635599
H	-2.492307	-0.304230	-1.600819
C	-3.404678	1.848323	0.952444
O	-3.153736	2.178597	2.092599
O	-4.273741	2.538351	0.170590
C	-2.795703	0.683731	0.264902
C	-1.898907	-0.201588	1.097678
H	-1.862872	0.180732	2.118218
H	-2.345278	-1.201416	1.140660
C	-4.867560	3.700092	0.775899
H	-5.522365	4.121061	0.015645
H	-4.097890	4.419232	1.059817
H	-5.437853	3.421122	1.663119
C	0.216232	-1.453804	1.330101
O	0.691124	-1.357100	2.434231
O	0.160738	-2.622964	0.648469
C	-0.443711	-0.323566	0.553389
C	0.327819	1.003982	0.690558
H	0.752898	1.068021	1.695553
H	-0.387341	1.825240	0.596776
C	0.691941	-3.773660	1.337369
H	0.567479	-4.607787	0.650070
H	0.139476	-3.951907	2.260537
H	1.746515	-3.622266	1.570598
H	-0.489825	-0.628216	-0.495464
H	2.194728	-0.765316	-0.022229
H	5.487195	-2.241071	2.668206
H	6.044615	-0.604942	2.202136
C	5.945319	-1.643269	1.883496
H	6.929918	-2.034753	1.624107
O	5.053140	-1.739613	0.756170
C	5.419043	-1.051954	-0.350041
O	6.444670	-0.418041	-0.422345
C	4.385797	-1.173323	-1.449580
H	3.795206	-2.076595	-1.283840
C	3.435947	0.052074	-1.560073
H	2.737130	-0.208246	-2.366605
C	4.186553	1.319902	-1.990834
H	4.779415	1.127941	-2.889416
H	3.491081	2.128813	-2.227820
H	4.867197	1.671693	-1.213310
C	-4.141729	-2.281517	-0.905513
O	-4.134295	-2.859707	0.164901
O	-3.446943	-2.723149	-1.988672
C	-4.850608	-1.033880	-1.188646
C	-2.682044	-3.926492	-1.795812
H	-2.241567	-4.148215	-2.765791
H	-3.329151	-4.743378	-1.473366
H	-1.902622	-3.766076	-1.048821
H	-4.965257	-0.789976	-2.238660
C	-5.886852	-0.557021	-0.226968
H	-6.107873	0.501273	-0.374236
H	-6.821315	-1.116168	-0.374226
H	-5.574454	-0.723109	0.805014
H	4.932020	-1.282395	-2.389591

Methyl acrylate MCR trimer with chirality R-MCR-R
Standard orientation Energy= -959.653910303

C	-1.935937	3.109233	0.810660
H	-2.286875	2.887927	1.820457
H	-2.703293	3.710626	0.317717
C	-2.929961	0.990828	-0.103710
O	-3.863913	1.002059	0.660466
O	-2.883809	0.204774	-1.203854
C	-1.662600	1.819043	0.033357
C	-0.543287	0.980357	0.738033
H	-0.916795	0.650535	1.708160
H	0.291955	1.668278	0.906886
C	-4.014084	-0.663970	-1.408758
H	-3.790374	-1.224174	-2.313903
H	-4.925648	-0.077989	-1.534094
H	-4.135689	-1.337157	-0.559249
H	-1.320822	2.060138	-0.976998
C	-0.378499	-1.557259	0.256116
O	0.006760	-2.527919	-0.378646
O	-1.190154	-1.675103	1.342771
C	-0.032066	-0.178682	-0.056372
C	0.838962	0.045953	-1.256044
H	0.438587	-0.529066	-2.097304
H	0.822314	1.103284	-1.530889
C	-1.557466	-3.013829	1.706931
H	-2.213005	-2.911198	2.569299
H	-0.672365	-3.598147	1.965240
H	-2.078517	-3.508371	0.885307
C	2.312993	-0.394365	-1.078740
H	-1.023347	3.705989	0.879599
C	3.020205	0.444955	-0.027919
O	2.733930	1.579020	0.281804
O	4.060713	-0.222829	0.511411
C	4.848718	0.498410	1.477700
H	4.232404	0.793366	2.327906
H	5.627258	-0.193543	1.790981
H	5.285433	1.389835	1.025284
C	3.084264	-0.287400	-2.409812
H	3.081138	0.740338	-2.784088
H	4.120796	-0.608662	-2.289069
H	2.615437	-0.926794	-3.161330
H	2.335733	-1.434877	-0.750186

Methyl acrylate MCR propagation TS with chirality R-MCR-R
MA4MCRP-RR-2.log52
Standard orientation Energy= -1266.19658104

C	2.588758	-1.735353	2.973831
H	3.219069	-0.849022	3.068150
H	3.225309	-2.611220	3.118149
C	2.936913	-1.847388	0.494590
O	4.061222	-1.414359	0.550784
O	2.437614	-2.474126	-0.595744
C	1.899774	-1.772775	1.605062
C	0.973123	-0.526590	1.439981
H	1.593600	0.369681	1.438847
H	0.355262	-0.496007	2.343340
C	3.321590	-2.581776	-1.728386
H	2.732015	-3.047796	-2.514395
H	4.185191	-3.198675	-1.475701
H	3.665270	-1.594774	-2.038625
H	1.284669	-2.673229	1.531777
C	0.560680	-0.027391	-1.062059
O	0.081846	-0.308743	-2.143758
O	1.626359	0.791530	-0.923967
C	0.047862	-0.524855	0.239656
C	-0.912528	-1.697656	0.156480
H	-0.338548	-2.510253	-0.307508
H	-1.155556	-2.027705	1.170008
C	2.113624	1.401905	-2.134487
H	3.026731	1.919727	-1.847977
H	1.372808	2.105956	-2.514412
H	2.321597	0.643328	-2.889538

C	-2.232382	-1.561088	-0.634852
H	1.841707	-1.720971	3.771347
C	-3.377068	-1.091452	0.249885
O	-3.443797	-1.210771	1.452622
O	-4.363847	-0.542350	-0.486810
C	-5.537665	-0.117353	0.231863
H	-5.279011	0.645936	0.966832
H	-6.207099	0.289650	-0.522614
H	-6.000577	-0.963706	0.741176
C	-2.636427	-2.916342	-1.257623
H	-2.727204	-3.690615	-0.489912
H	-3.588962	-2.838260	-1.784994
H	-1.873988	-3.228763	-1.974196
H	-2.115798	-0.848401	-1.449936
C	-0.055269	3.367034	0.224044
O	-0.303338	3.416352	-0.964285
O	0.646373	4.331385	0.873085
C	-0.466447	2.307150	1.148755
C	-1.219420	1.237247	0.732317
H	-1.674844	1.284899	-0.250167
H	-1.705902	0.620024	1.476090
C	1.074610	5.443791	0.072095
H	1.593203	6.112736	0.755640
H	0.216326	5.944931	-0.378490
H	1.747506	5.110512	-0.719786
H	-0.130611	2.398016	2.174575

Methyl acrylate MCR trimer with chirality R-MCR-S
Standard orientation Energy= -959.651025646

C	2.707013	-2.357660	1.716757
H	2.994607	-1.706934	2.544697
H	3.605668	-2.876788	1.375966
C	3.061172	-0.528261	0.033734
O	3.956910	-0.014744	0.657957
O	2.805852	-0.251570	-1.263875
C	2.074102	-1.548049	0.581627
C	0.771827	-0.833185	1.075348
H	1.046430	-0.100196	1.834809
H	0.171008	-1.606826	1.568441
C	3.651169	0.739877	-1.878677
H	3.283174	0.844805	-2.896767
H	4.689803	0.406458	-1.876868
H	3.577850	1.686570	-1.342278
H	1.805492	-2.213625	-0.243435
C	-0.055404	1.239684	-0.226528
O	-0.752280	1.813086	-1.048075
O	0.814305	1.905320	0.582702
C	-0.053708	-0.197360	0.001188
C	-0.918768	-1.048158	-0.872032
H	-1.151596	-0.517211	-1.797068
H	-0.366877	-1.956718	-1.139752
C	0.858163	3.330307	0.417575
H	1.622145	3.680029	1.108931
H	-0.109012	3.775103	0.658875
H	1.119131	3.592212	-0.609235
C	-2.260120	-1.526510	-0.220798
H	2.000809	-3.104898	2.087570
C	-3.207935	-0.347418	-0.059515
O	-4.097071	-0.058136	-0.821122
O	-2.923711	0.359933	1.056045
C	-3.695443	1.561840	1.250363
H	-4.759337	1.327298	1.299989
H	-3.350424	1.979027	2.194080
H	-3.514236	2.257628	0.430355
C	-2.906519	-2.629423	-1.062948
H	-2.233152	-3.486566	-1.144941
H	-3.841543	-2.970478	-0.613106
H	-3.136052	-2.267526	-2.066924
H	-2.039224	-1.906583	0.781038

Methyl acrylate MCR propagation TS with chirality R-MCR-S
Standard orientation Energy= -1266.19493231

C	-1.936435	1.493328	-2.887587
H	-1.263519	2.200535	-3.381265
H	-2.939019	1.924850	-2.868994
C	-1.507341	2.444248	-0.631285
O	-0.575650	3.161489	-0.351278
O	-2.778369	2.715631	-0.266034
C	-1.441811	1.167828	-1.460863
C	-0.005358	0.573125	-1.492032
H	0.702429	1.376987	-1.280942
H	0.183155	0.234430	-2.511447
C	-2.983129	3.940038	0.464849
H	-4.050328	3.979094	0.671432
H	-2.675555	4.798013	-0.134594
H	-2.412479	3.929943	1.394313
H	-2.144663	0.460756	-1.015620
C	-0.377368	-1.888102	-0.830034
O	-0.398385	-2.833472	-0.069441
O	-0.903041	-1.951220	-2.083540
C	0.265374	-0.581935	-0.547049
C	0.589907	-0.305226	0.903734
H	1.054594	-1.184908	1.352751
H	1.328237	0.498909	0.930765
C	-1.481229	-3.214960	-2.454607
H	-1.824639	-3.088822	-3.479414
H	-2.317768	-3.460903	-1.799086
H	-0.737340	-4.010672	-2.393872
C	-0.574797	0.147398	1.842521
H	-1.964801	0.576847	-3.480358
C	-1.539037	-0.991358	2.137488
O	-1.455269	-1.756245	3.064577
O	-2.542351	-1.037899	1.226696
C	-3.469554	-2.129584	1.381663
H	-3.941145	-2.092100	2.364290
H	-4.212171	-1.995930	0.597240
H	-2.947116	-3.079920	1.266074
C	0.009621	0.691822	3.152275
H	0.651465	1.551623	2.946752
H	-0.783052	1.009454	3.833842
H	0.598690	-0.073445	3.660842
H	-1.147285	0.935636	1.353003
C	4.006443	-0.057594	-0.088089
O	4.023901	-0.740281	0.916074
O	4.838815	0.996944	-0.282226
C	3.122474	-0.248262	-1.242196
C	2.202406	-1.273583	-1.283142
H	2.279274	-2.063792	-0.545361
H	1.770508	-1.537349	-2.240960
C	5.769538	1.263776	0.779701
H	6.348021	2.124191	0.450239
H	6.420033	0.403148	0.943869
H	5.239060	1.490182	1.706026
H	3.209981	0.467913	-2.050220

Butyl acrylate monomer
Standard orientation Energy= -424.541316999

C	1.187342	-1.338207	0.000000
O	2.257205	-0.773628	0.000000
O	0.000000	-0.693777	0.000000
C	0.982043	-2.810005	0.000000
C	2.016061	-3.648918	0.000000

H	3.032263	-3.270343	0.000000
H	1.872623	-4.722870	0.000000
C	0.049858	0.753280	0.000000
H	0.603615	1.083782	-0.882780
H	0.603615	1.083782	0.882780
H	-0.044513	-3.157674	0.000000
C	-1.378361	1.268853	0.000000
C	-1.441370	2.801371	0.000000
C	-2.876377	3.336162	0.000000
H	-1.900099	0.875299	-0.879253
H	-1.900099	0.875299	0.879253
H	-0.908554	3.188411	-0.876653
H	-0.908554	3.188411	0.876653
H	-2.892369	4.429224	0.000000
H	-3.425372	2.995350	0.883178
H	-3.425372	2.995350	-0.883178

Methyl acrylate dimer
Standard orientation Energy= -613.734044849

C	2.309356	-0.128541	0.059528
O	2.695976	-1.047220	-0.644441
O	3.037995	1.002386	0.264383
C	1.047341	-0.091138	0.769453
C	0.084782	-1.219853	0.689607
H	0.606432	-2.114225	0.342289
H	-0.327750	-1.415078	1.685320
C	4.309032	1.054914	-0.400810
H	4.744089	2.012594	-0.122674
H	4.179741	0.992573	-1.482902
H	4.948790	0.233627	-0.072785
H	0.806779	0.796172	1.342063
C	-1.951493	0.208006	0.201743
O	-2.047225	0.588547	1.344499
O	-2.629796	0.765409	-0.822655
C	-1.103426	-0.961825	-0.272837
C	-3.515329	1.850191	-0.483355
H	-3.959387	2.166777	-1.424395
H	-2.956992	2.668412	-0.026968
H	-4.285463	1.510669	0.210618
H	-0.717745	-0.710229	-1.264360
C	-1.991746	-2.215281	-0.400238
H	-1.400746	-3.053866	-0.775499
H	-2.407977	-2.497958	0.570631
H	-2.816474	-2.041279	-1.094163

Chain transfer to monomer TS on V2
Standard orientation Energy= -1038.23609328

C	-1.865847	-2.469769	-0.593509
O	-2.734093	-3.238462	-0.940818
O	-2.085736	-1.219935	-0.157348
C	-0.421888	-2.787672	-0.603990
C	0.155515	-3.934397	-0.910627
H	-0.440555	-4.801690	-1.193043
H	1.234645	-4.053650	-0.894948
C	-3.463602	-0.766170	-0.125348
H	-4.049461	-1.469694	0.471499
H	-3.855430	-0.784313	-1.146062
H	0.473790	-1.660493	-0.231713
C	-3.473764	0.633627	0.462529
C	-4.883693	1.236557	0.491311
C	-4.913126	2.642831	1.096869
H	-3.065331	0.595448	1.478468
H	-2.803221	1.272415	-0.121401
H	-5.552586	0.580289	1.061167
H	-5.285582	1.271346	-0.528342
H	-5.926775	3.052417	1.099411
H	-4.276200	3.328995	0.530808
H	-4.554552	2.635090	2.130805
C	2.407197	-1.296464	0.673557
O	3.426011	-1.643417	0.114562
O	2.201012	-1.465209	2.000904
C	1.217662	-0.691199	0.009005
C	1.479282	-0.007293	-1.321515
H	2.069031	-0.673084	-1.956341
H	0.523476	0.173298	-1.820051
C	3.262795	-2.107512	2.728461
H	2.921110	-2.153657	3.760251
H	4.183569	-1.526991	2.654735
H	3.441848	-3.111087	2.339298
H	0.602745	-0.118248	0.702013
C	1.405298	2.368723	-0.464955
O	0.205583	2.494761	-0.542749
O	2.184219	3.184444	0.278652
C	2.234995	1.330040	-1.202914
C	1.508182	4.252260	0.969009
H	2.287363	4.791737	1.502928
H	0.772768	3.849792	1.666878
H	1.004810	4.908461	0.257522
H	3.160606	1.181216	-0.641559
C	2.598227	1.888165	-2.594296
H	3.232333	1.174410	-3.125333
H	1.698395	2.059031	-3.191268
H	3.143844	2.831154	-2.512871

Chain transfer to monomer TS on A1
Standard orientation Energy= -1038.24753848

C	3.473360	-0.102262	0.315726
O	3.557547	-0.545425	-0.804195
O	2.610764	0.901052	0.663604
C	4.260289	-0.526204	1.494463
C	5.180403	-1.484989	1.398006
H	5.369946	-1.975343	0.449838
H	5.758214	-1.800764	2.258227
C	1.757253	1.420916	-0.323672
H	0.669851	0.573793	-0.353735
H	2.162155	1.288053	-1.326752
H	4.046388	-0.021811	2.429182
C	1.279796	2.800230	0.044482
C	0.303251	3.393733	-0.979250
C	-0.109726	4.826886	-0.632819
H	0.817777	2.774324	1.037959
H	2.159077	3.455096	0.136059
H	-0.590757	2.765485	-1.042473
H	0.765954	3.376630	-1.972954
H	-0.806337	5.225847	-1.374413
H	0.757896	5.493191	-0.597115
H	-0.604016	4.870674	0.342385
C	0.201282	-1.565073	-0.672331
O	0.517315	-2.425901	0.124606
O	0.404683	-1.678875	-2.006122
C	-0.391365	-0.252749	-0.327725
C	-0.990039	-0.121585	1.056117
H	-0.298926	-0.550655	1.785589
H	-1.120505	0.936963	1.297924
C	1.123347	-2.848643	-2.436117
H	1.154906	-2.786381	-3.522004
H	0.607228	-3.755660	-2.118166
H	2.133306	-2.838212	-2.024115
H	-0.978040	0.176619	-1.137961
C	-3.412027	-0.186670	0.341337
O	-3.425383	0.967083	-0.020793
O	-4.387313	-1.066429	0.028618

C	-2.350481	-0.827439	1.221072
C	-5.480620	-0.551854	-0.754702
H	-6.152103	-1.394466	-0.904164
H	-5.119118	-0.174620	-1.712204
H	-5.987250	0.253511	-0.220919
H	-2.257299	-1.874300	0.920943
C	-2.826699	-0.788699	2.687899
H	-2.093636	-1.285627	3.327577
H	-2.939678	0.242550	3.034305
H	-3.784085	-1.300521	2.806324

Chain transfer to monomer TS on A2
Standard orientation Energy= -1038.24530698

C	4.012367	-0.066275	0.292475
O	4.792659	0.393515	-0.509722
O	2.807960	0.487127	0.563047
C	4.216453	-1.290691	1.107318
C	5.361994	-1.967569	1.052149
H	6.170079	-1.633821	0.410531
H	5.516709	-2.863338	1.641707
C	2.461046	1.674154	-0.184445
H	2.454686	1.434366	-1.251429
H	3.238619	2.431572	-0.023322
H	3.388452	-1.600438	1.733712
C	1.119177	2.149976	0.292137
C	0.534756	3.320941	-0.466919
C	-0.794767	3.830590	0.099837
H	0.264339	1.063831	0.075571
H	1.061004	2.240514	1.379221
H	0.410880	3.046984	-1.522026
H	1.265480	4.144698	-0.462303
H	-1.148793	4.697800	-0.463235
H	-0.682987	4.136987	1.144421
H	-1.575123	3.066989	0.055522
C	0.139473	-1.057535	-0.626739
O	0.388378	-2.058715	0.012427
O	0.576121	-0.871063	-1.900886
C	-0.629842	0.112551	-0.143985
C	-1.375011	-0.073861	1.162127
H	-0.712527	-0.560720	1.881980
H	-1.645344	0.905562	1.566490
C	1.352247	-1.943147	-2.463720
H	1.599678	-1.624353	-3.474182
H	0.770687	-2.866178	-2.485212
H	2.261145	-2.107936	-1.883143
H	-1.201001	0.591216	-0.938599
C	-3.690462	-0.239099	0.167964
O	-3.777804	0.951985	-0.023529
O	-4.550377	-1.133183	-0.365181
C	-2.654525	-0.923396	1.045347
C	-5.615056	-0.590641	-1.168829
H	-6.190684	-1.449846	-1.505947
H	-5.209115	-0.042554	-2.020086
H	-6.237796	0.080294	-0.575359
H	-2.414048	-1.885583	0.585770
C	-3.273711	-1.196612	2.431849
H	-2.554541	-1.728612	3.058970
H	-3.537102	-0.260760	2.933020
H	-4.173034	-1.810322	2.349298

Chain transfer to monomer TS on A3
Standard orientation Energy= -1038.24575022

C	-4.378827	0.572187	-0.485641
O	-3.823271	0.960248	0.517193
O	-3.768558	-0.122767	-1.469873
C	-5.811113	0.787682	-0.818382
C	-6.619507	1.443947	0.011567
H	-6.245281	1.835189	0.951113
H	-7.664269	1.604533	-0.226352
C	-2.356819	-0.418076	-1.314915
H	-1.867595	0.420880	-0.820514
H	-1.991615	-0.510237	-2.338925
H	-6.156384	0.384709	-1.763266
C	-2.147174	-1.717528	-0.540918
C	-0.746585	-2.279194	-0.670078
C	-0.448177	-3.509909	0.153895
H	-2.400364	-1.561342	0.512461
H	-2.859329	-2.463139	-0.926413
H	0.147532	-1.306514	-0.206557
H	-0.405428	-2.352934	-1.706261
H	0.585874	-3.839652	0.030262
H	-1.095827	-4.345343	-0.146389
H	-0.623171	-3.331175	1.218996
C	0.571351	0.128760	1.429455
O	0.898026	-0.227316	2.544175
O	-0.372749	1.073610	1.199683
C	1.082683	-0.435303	0.158986
C	2.379162	-1.214950	0.240558
H	2.327722	-1.907166	1.084529
H	2.505098	-1.803722	-0.672784
C	-1.021490	1.631773	2.357924
H	-1.856399	2.209345	1.970736
H	-0.324245	2.265159	2.909980
H	-1.381999	0.838326	3.012655
H	1.021437	0.267321	-0.670802
C	3.862369	0.561406	-0.774708
O	3.540527	0.310603	-1.912800
O	4.520463	1.688771	-0.428098
C	3.631106	-0.336278	0.430216
C	4.849813	2.588193	-1.503267
H	5.366102	3.423342	-1.035049
H	3.943856	2.929976	-2.005524
H	5.497022	2.094584	-2.229712
H	3.496606	0.306921	1.303593
C	4.890211	-1.198228	0.657671
H	4.758786	-1.816985	1.548449
H	5.069415	-1.859172	-0.195082
H	5.774934	-0.574997	0.804435

Chain transfer to monomer TS on A4
Standard orientation Energy= -1038.24231367

C	5.326168	0.531911	0.200797
O	5.964976	-0.488167	0.322345
O	4.085824	0.579442	-0.333021
C	5.772631	1.890451	0.604003
C	6.970690	2.086274	1.151030
H	7.645249	1.252666	1.312322
H	7.301720	3.074214	1.448255
C	3.520943	-0.680277	-0.763310
H	3.494776	-1.362244	0.090598
H	4.178075	-1.118177	-1.519921
H	5.075881	2.702403	0.431295
C	2.130939	-0.402541	-1.308776
C	1.455713	-1.681075	-1.824214
C	0.092341	-1.450792	-2.427360
H	1.524327	0.051287	-0.519853
H	2.205949	0.327835	-2.121731
H	1.381182	-2.418584	-1.015463
H	2.100499	-2.147894	-2.585506
H	-0.419050	-2.340657	-2.791268
H	0.009752	-0.608497	-3.114393
H	-0.792794	-1.060853	-1.388999

C	-1.102988	-1.173547	0.786910
O	-0.431101	-0.512571	1.553211
O	-1.378190	-2.486697	0.998086
C	-1.670761	-0.696840	-0.495874
C	-1.822699	0.805158	-0.644605
H	-0.887237	1.291450	-0.356755
H	-2.013844	1.041697	-1.695012
C	-0.829031	-3.060204	2.196759
H	-1.151098	-4.099482	2.195908
H	-1.209345	-2.542503	3.078683
H	0.260483	-2.997202	2.191019
H	-2.533649	-1.279144	-0.815481
C	-4.312998	0.896422	-0.225211
O	-4.610696	0.549966	-1.344303
O	-5.190513	0.901726	0.801632
C	-2.951150	1.414494	0.208919
C	-6.535238	0.491271	0.489655
H	-7.084016	0.559268	1.426383
H	-6.543773	-0.532367	0.112969
H	-6.971892	1.152073	-0.260574
H	-2.808089	1.138070	1.256649
C	-2.952459	2.954417	0.114647
H	-1.995229	3.347575	0.465066
H	-3.099376	3.281239	-0.918568
H	-3.743507	3.387502	0.730573

Chain transfer to monomer TS on V1

Standard orientation Energy= -1038.23345887

C	-1.711924	1.321275	-0.930342
O	-2.187569	1.670544	-1.987337
O	-0.929380	2.124726	-0.170325
C	-1.859883	-0.031272	-0.313264
C	-2.941106	-0.914348	-0.911592
H	-2.826260	-0.933860	-1.998314
H	-2.807685	-1.934453	-0.541219
C	-0.676967	3.440095	-0.697938
H	-0.042149	3.930832	0.036658
H	-1.612302	3.987451	-0.822826
H	-0.169811	3.374599	-1.661673
H	-1.869521	0.016729	0.775918
C	-4.672923	-0.541860	0.892159
O	-4.138466	-1.289760	1.676918
O	-5.653083	0.317840	1.245219
C	-4.378637	-0.460279	-0.597357
C	-6.062903	0.281337	2.625454
H	-6.845078	1.031848	2.715714
H	-5.223226	0.520919	3.279164
H	-6.446544	-0.706698	2.883790
H	-4.510210	0.579745	-0.906223
C	-5.410872	-1.318553	-1.357901
H	-5.241856	-1.233648	-2.433876
H	-5.322482	-2.372557	-1.079814
H	-6.430026	-0.988651	-1.146534
C	2.976339	-1.221862	-0.454312
O	3.245446	-2.291508	-0.946919
O	3.895295	-0.372284	0.052394
C	1.605892	-0.642034	-0.312019
C	0.534672	-1.285446	-0.734140
H	0.451444	-2.254486	-1.212289
H	-0.776073	-0.586878	-0.530557
C	5.277330	-0.803027	-0.012841
H	5.536987	-0.984458	-1.059122
H	5.372485	-1.751030	0.523127
H	1.540813	0.334857	0.158026
C	6.134677	0.287375	0.604757
C	7.626087	-0.070377	0.588406
C	8.504858	1.020209	1.207573
H	5.970562	1.223065	0.059039
H	5.804847	0.460579	1.634989
H	7.945988	-0.253294	-0.444279
H	7.780934	-1.012540	1.127433
H	9.561100	0.739627	1.183190
H	8.232819	1.201818	2.251861
H	8.399459	1.966621	0.668359

Methyl acrylate trimer molecule with chirality RRR

Standard orientation Energy= -960.296801360

C	-2.245765	-1.903256	-0.091832
O	-2.556359	-2.955727	-0.592324
O	-3.095662	-0.850132	-0.007274
C	-0.883394	-1.560727	0.493346
C	-0.135185	-2.837454	0.888865
H	0.092624	-3.446246	0.012636
H	-0.736496	-3.443134	1.570487
C	-4.395452	-1.041429	-0.598604
H	-4.926185	-0.103748	-0.447747
H	-4.919646	-1.863184	-0.109001
H	-4.301193	-1.259808	-1.663005
H	-1.069144	-0.952725	1.385979
C	1.718843	3.741710	-0.710383
H	1.356613	3.806901	-1.738318
H	1.426057	4.657272	-0.190162
C	-0.371896	2.595148	0.017634
O	-1.070958	2.815692	-0.942625
O	-0.859819	2.410016	1.262946
C	1.146212	2.497605	-0.011957
C	1.627838	1.209240	-0.717097
H	1.273192	1.197520	-1.752164
H	2.718910	1.276965	-0.761816
C	-2.295634	2.475306	1.400536
H	-2.485768	2.401783	2.469166
H	-2.760128	1.643228	0.870503
H	-2.671219	3.419858	1.006109
H	1.494118	2.469771	1.024004
C	2.350540	-1.113267	-0.244767
O	2.477078	-1.821944	-1.213233
O	3.255532	-1.066163	0.759537
C	1.220750	-0.120135	-0.014332
C	-0.126566	-0.670073	-0.525494
H	0.058901	-1.236714	-1.443196
H	-0.773549	0.165129	-0.796948
C	4.413856	-1.909753	0.606082
H	5.016292	-1.740111	1.495779
H	4.968739	-1.636327	-0.292459
H	4.115890	-2.956696	0.537134
H	1.167928	0.068457	1.061857
H	0.800061	-2.595630	1.399028
H	2.810627	3.696002	-0.726059

Methyl acrylate trimer molecule with chirality RRS

Standard orientation Energy= -960.301194407

C	3.197166	-0.227414	-0.596671
O	3.026202	-1.099505	-1.416242
O	3.856629	0.919589	-0.870603
C	2.754600	-0.277426	0.857484
C	3.917123	-0.833704	1.706594
H	3.637112	-0.831172	2.762543
H	4.817486	-0.226257	1.592147
C	4.381064	1.046725	-2.205644
H	4.870277	2.017897	-2.234072
H	3.574132	1.001732	-2.938249
H	5.096686	0.249681	-2.412527

H	2.565332	0.748863	1.182209
C	-2.922337	-2.714219	-0.650704
H	-3.328936	-3.002381	0.322608
H	-3.751617	-2.599065	-1.351552
C	-3.058463	-0.295748	-0.145880
O	-3.285391	0.079120	0.979667
O	-3.699273	0.193516	-1.230181
C	-2.100737	-1.409868	-0.536926
C	-0.939140	-1.584927	0.455084
H	-1.327546	-1.588633	1.477798
H	-0.509186	-2.574665	0.274745
C	-4.710686	1.184792	-0.976349
H	-5.118294	1.441025	-1.951902
H	-4.274050	2.064402	-0.501692
H	-5.489750	0.779879	-0.328710
H	-1.708203	-1.169116	-1.529147
C	-0.151877	0.771240	0.950958
O	-0.016209	1.072091	2.111806
O	-0.670360	1.610625	0.026136
C	0.228723	-0.569921	0.340131
C	1.487454	-1.127567	1.028736
H	1.683007	-2.126114	0.628714
H	1.285890	-1.230312	2.098421
C	-1.130220	2.881832	0.525570
H	-1.489598	3.423619	-0.347033
H	-0.311437	3.422957	1.001166
H	-1.932736	2.730599	1.248458
H	0.434249	-0.411190	-0.722153
H	4.153370	-1.861502	1.417328
H	-2.284986	-3.526508	-1.008064

Methyl acrylate trimer molecule with chirality RSR

Standard orientation Energy= -960.302977613

C	-2.783595	0.700456	-0.591321
O	-2.419755	1.380271	-1.520546
O	-3.456598	1.198251	0.469634
C	-2.601009	-0.805115	-0.476513
C	-3.789038	-1.495654	-1.178343
H	-3.800783	-1.255975	-2.244858
H	-4.741653	-1.184138	-0.743787
C	-3.738279	2.610141	0.431804
H	-4.273452	2.824438	1.354351
H	-4.353977	2.853791	-0.435228
H	-2.810700	3.181925	0.383385
H	-2.641775	-1.062511	0.585580
C	3.789792	-1.496072	-1.176293
H	3.802075	-1.257026	-2.242943
H	4.742206	-1.184331	-0.741454
C	2.784122	0.700431	-0.591098
O	2.421268	1.379592	-1.521186
O	3.455946	1.198990	0.470246
C	2.601446	-0.805071	-0.475467
C	1.264953	-1.259634	-1.081852
H	1.219499	-0.926276	-2.122160
H	1.245280	-2.353727	-1.082496
C	3.737639	2.610861	0.431722
H	4.272313	2.825715	1.354430
H	2.810070	3.182598	0.382488
H	4.353776	2.854013	-0.435133
H	2.641657	-1.061834	0.586799
C	-0.000197	-1.225478	1.083799
O	0.000046	-2.383602	1.431311
O	-0.000931	-0.194772	1.956786
C	0.000220	-0.748177	-0.360064
C	-1.264246	-1.259403	-1.082499
H	-1.244566	-2.353499	-1.083532
H	-1.218363	-0.925680	-2.122672
C	-0.001426	-0.546302	3.354160
H	-0.001721	0.399560	3.891148
H	0.887482	-1.128237	3.601086
H	-0.890427	-1.128388	3.600408
H	0.000284	0.343859	-0.363774
H	-3.705540	-2.579833	-1.071228
H	3.706193	-2.580181	-1.068567

Methyl acrylate chain transfer to polymer TS with chirality RRR

Standard orientation Energy= -1267.41200913

C	3.189681	-1.460648	-0.728584
O	3.149104	-2.002337	-1.806896
O	3.827864	-1.981546	0.339639
C	2.635073	-0.077109	-0.420713
C	3.703694	0.960788	-0.828146
H	3.864770	0.941479	-1.909628
H	4.657852	0.754138	-0.337256
C	4.415452	-3.279817	0.145452
H	4.882748	-3.533253	1.094943
H	5.158881	-3.250136	-0.652693
H	3.643446	-4.007211	-0.108899
H	2.495122	-0.013715	0.659447
C	-3.534313	1.366934	-1.440968
H	-3.574081	1.069687	-2.492878
H	-4.550829	1.364713	-1.043292
C	-3.254959	-0.963179	-0.628159
O	-2.950129	-1.885576	-1.345628
O	-4.277724	-1.023535	0.253635
C	-2.619088	0.418347	-0.634236
C	-1.205515	0.401868	-1.240892
H	-1.221711	-0.252264	-2.121520
H	-0.993167	1.408990	-1.610555
C	-5.026674	-2.252677	0.269961
H	-5.793747	-2.116066	1.029301
H	-4.379955	-3.093476	0.524301
H	-5.480851	-2.436642	-0.704780
H	-2.596979	0.765312	0.399622
C	-0.093032	-1.271007	0.406844
O	0.689880	-2.191233	0.347413
O	-1.150041	-1.268995	1.257464
C	0.001638	-0.003060	-0.382892
C	1.302968	0.174910	-1.162570
H	1.304501	1.200698	-1.544332
H	1.276395	-0.472833	-2.047198
C	-1.323219	-2.465013	2.041092
H	-2.197185	-2.280396	2.662192
H	-0.443311	-2.646908	2.659578
H	-1.485446	-3.323987	1.388646
H	0.034749	0.957125	0.576632
H	3.381615	1.962346	-0.535488
H	-3.147892	2.387212	-1.388151
C	0.562955	3.132742	0.802461
O	1.715046	3.511238	0.850417
O	-0.377364	3.737240	0.026067
C	0.007764	1.964561	1.517632
C	0.072189	4.875836	-0.729769
H	-0.803042	5.231947	-1.269204
H	0.861084	4.588823	-1.427007
H	0.452359	5.651314	-0.063323
H	-1.068585	2.030501	1.661099
C	0.790014	1.463407	2.706806
H	0.437416	0.477725	3.017524
H	1.856802	1.409884	2.486580
H	0.668178	2.141649	3.559543

Methyl acrylate chain transfer to polymer TS with chirality RRS
Standard orientation Energy= -1267.41344532

C	3.567772	-0.723014	-0.246470
O	3.674323	-0.788397	-1.449534
O	4.364371	0.057491	0.516718
C	2.594381	-1.515719	0.611289
C	3.334721	-2.765277	1.138706
H	2.685850	-3.301800	1.833620
H	4.249988	-2.489651	1.666589
C	5.400500	0.780461	-0.174457
H	5.927273	1.339356	0.595818
H	4.968849	1.458155	-0.912292
H	6.077152	0.088630	-0.678093
H	2.334600	-0.902474	1.474697
C	-2.710944	-0.614057	-2.844345
H	-2.864156	-1.661066	-3.122541
H	-3.659128	-0.084640	-2.950954
C	-3.238936	-1.019061	-0.454031
O	-3.253133	-2.112666	0.061583
O	-4.236602	-0.122301	-0.298022
C	-2.170966	-0.495017	-1.400700
C	-0.855358	-1.280355	-1.262422
H	-1.105372	-2.346849	-1.192443
H	-0.296158	-1.165193	-2.195418
C	-5.356325	-0.552096	0.496105
H	-6.046282	0.289114	0.505249
H	-5.037396	-0.797801	1.510103
H	-5.827904	-1.429433	0.050689
H	-2.026100	0.564001	-1.181570
C	-0.407442	-0.802250	1.263171
O	-0.001996	-1.400901	2.235520
O	-1.380535	0.133604	1.338411
C	0.130483	-0.966430	-0.128109
C	1.328991	-1.914784	-0.174485
H	1.607833	-2.086048	-1.216730
H	0.977015	-2.875506	0.218316
C	-1.949310	0.340144	2.644297
H	-2.716088	1.100067	2.509257
H	-1.184249	0.683409	3.341924
H	-2.385743	-0.586934	3.018137
H	0.570322	0.295454	-0.426264
H	3.596831	-3.438969	0.317819
H	-1.998357	-0.176381	-3.547279
C	0.087192	2.505548	-0.333031
O	-0.888936	2.911113	-0.928980
O	0.410726	2.916865	0.919562
C	1.057531	1.512244	-0.847740
C	-0.485437	3.869365	1.514339
H	-0.090328	4.060144	2.510141
H	-1.495930	3.462086	1.571246
H	-0.507776	4.790625	0.929782
H	1.995994	1.532893	-0.297503
C	1.184779	1.420523	-2.349810
H	1.786875	0.557748	-2.638855
H	0.204558	1.365959	-2.825585
H	1.682303	2.315298	-2.743768

Methyl acrylate chain transfer to polymer TS with chirality RSR
Standard orientation Energy= -1267.41060073

C	3.031093	-0.501005	-0.769667
O	2.680635	-1.402646	-1.495536
O	3.971806	-0.635699	0.182205
C	2.532881	0.937867	-0.849258
C	3.505002	1.722692	-1.756713
H	3.474001	1.339088	-2.780353
H	4.532143	1.651161	-1.392621
C	4.569341	-1.945702	0.312326
H	5.326370	-1.838383	1.086267
H	5.025248	-2.246185	-0.632012
H	3.810088	-2.667476	0.609754
H	2.595567	1.366058	0.152468
C	-3.411384	2.083512	-0.492397
H	-3.672439	2.175093	-1.550835
H	-4.326336	2.156489	0.099068
C	-3.635289	-0.385941	-0.584208
O	-3.660549	-0.987327	-1.631537
O	-4.504157	-0.626351	0.423609
C	-2.685791	0.746648	-0.225327
C	-1.371027	0.609831	-1.022207
H	-1.237250	-0.433909	-1.312418
H	-1.473514	1.158857	-1.967895
C	-5.496167	-1.639397	0.175546
H	-6.093388	-1.689800	1.083466
H	-5.018649	-2.599731	-0.023551
H	-6.117701	-1.365394	-0.678280
H	-2.493373	0.686614	0.845713
C	-0.108998	2.264913	0.511366
O	-0.831854	2.384831	1.478939
O	0.766791	3.232530	0.143305
C	-0.050949	1.053666	-0.371184
C	1.089040	1.006811	-1.388080
H	0.994721	1.902324	-2.012981
H	0.931883	0.151714	-2.049334
C	0.792705	4.408411	0.974766
H	1.543409	5.059752	0.532351
H	1.065191	4.146595	1.998065
H	-0.183208	4.895318	0.979258
H	0.148995	0.089536	0.575555
H	3.218379	2.776465	-1.775895
H	-2.770525	2.922162	-0.217900
C	0.502107	-2.122450	1.028615
O	1.502009	-2.749937	1.310605
O	-0.399591	-2.536244	0.109644
C	0.117770	-0.817123	1.618251
C	-0.074231	-3.749959	-0.596371
H	-0.897448	-3.908660	-1.289552
H	0.008314	-4.585189	0.100730
H	0.866164	-3.626586	-1.134624
H	-0.951627	-0.751731	1.811469
C	0.999070	-0.290010	2.722532
H	0.687219	0.713936	3.014836
H	0.926886	-0.933851	3.606844
H	2.049133	-0.274114	2.424590

Butyl acrylate trimer
Standard orientation Energy= -1313.56833012

C	-2.998226	-1.319047	-0.769501
O	-2.773616	-1.619752	-1.917788
O	-3.532343	-0.134040	-0.403807
C	-2.773226	-2.224672	0.432866
C	-4.012215	-3.128602	0.599600
H	-4.136169	-3.778690	-0.270504
H	-4.922460	-2.536384	0.720066
C	-3.855512	0.787733	-1.474224
H	-4.557374	0.300788	-2.156515
H	-2.943217	1.005053	-2.035667
H	-2.698031	-1.587697	1.318539
C	3.522115	-3.610068	-0.045843
H	3.424907	-4.193936	-0.965036
H	4.510599	-3.145339	-0.034568
C	2.562375	-1.578814	-1.110585
O	2.067097	-1.710486	-2.204397

O	3.377291	-0.552367	-0.783642
C	2.404129	-2.550604	0.049580
C	1.018255	-3.213381	0.050948
H	0.867156	-3.715554	-0.908455
H	1.009285	-3.980634	0.831446
C	3.663078	0.401511	-1.835960
H	2.725276	0.863214	-2.166098
H	4.054369	-0.153564	-2.702579
H	2.554836	-1.985634	0.973763
C	-0.013028	-1.550627	1.618729
O	-0.029165	-2.095204	2.698999
O	0.169066	-0.223290	1.462181
C	-0.177918	-2.264539	0.284986
C	-1.496249	-3.064089	0.272898
H	-1.463825	-3.797133	1.084469
H	-1.554602	-3.614234	-0.670211
C	0.360144	0.555665	2.670114
H	1.218204	0.153069	3.214589
H	-0.521976	0.437811	3.304976
H	-0.197981	-1.515095	-0.509136
H	-3.897407	-3.758091	1.485320
H	3.459256	-4.295317	0.802967
C	0.576316	2.001889	2.261135
H	-0.290050	2.342212	1.683320
H	1.443414	2.057060	1.594067
C	0.788984	2.920530	3.470642
H	1.651152	2.567949	4.049128
H	-0.076945	2.849850	4.139653
C	1.006971	4.383503	3.074093
H	1.156352	5.015308	3.953650
H	1.887034	4.492113	2.432855
H	0.146143	4.776679	2.524463
C	4.624574	1.404582	-1.318746
H	5.312418	1.090111	-0.540138
C	4.869133	2.677608	-2.056568
H	5.424070	2.472924	-2.989570
H	3.911183	3.101207	-2.388727
C	5.645121	3.720193	-1.242157
H	6.620106	3.330515	-0.935337
H	5.817475	4.627370	-1.826760
H	5.097242	3.999008	-0.338133
C	-4.448298	2.038293	-0.849079
H	-5.331307	1.760860	-0.262728
H	-3.723982	2.463360	-0.145467
C	-4.830369	3.087562	-1.900366
H	-5.547525	2.652005	-2.606203
H	-3.944652	3.352208	-2.490076
C	-5.429157	4.355330	-1.284486
H	-6.337032	4.128581	-0.717087
H	-4.722261	4.834572	-0.600128
H	-5.692299	5.084859	-2.054964

Butyl acrylate trimer with radical on the middle alkylic branch
TS to self-backbite with 8-ring structure
Standard orientation Energy= -1313.52463065

C	2.307030	1.466532	0.471341
O	2.432480	2.136285	1.469428
O	3.346054	0.883577	-0.164098
C	0.985711	1.221379	-0.242415
C	0.878290	2.227156	-1.408427
H	1.683329	2.065769	-2.129930
H	0.941529	3.255222	-1.043490
C	4.662212	1.098466	0.404160
H	4.663612	0.728958	1.432887
H	4.856990	2.173616	0.437103
H	0.218964	1.461947	0.493856
C	-1.668240	-4.466648	-1.915866
H	-0.948202	-5.039153	-1.327103
H	-2.600436	-5.035741	-1.945378
C	-2.358235	-3.139804	0.123177
O	-2.091964	-4.005907	0.919720
O	-3.014837	-1.995300	0.445663
C	-1.892533	-3.079527	-1.313594
C	-0.574187	-2.250519	-1.309593
H	0.195764	-2.832067	-0.790487
H	-0.250031	-2.167044	-2.354463
C	-2.798599	-1.499748	1.796846
H	-2.999694	-2.306343	2.505454
H	-3.548520	-0.719183	1.912212
H	-2.634881	-2.527719	-1.892627
C	-1.664594	0.027323	-1.316222
O	-2.127281	-0.131384	-2.425246
O	-2.065293	1.019118	-0.491355
C	-0.594632	-0.839825	-0.708168
C	0.823904	-0.244825	-0.710496
H	1.236148	-0.338542	-1.723788
H	1.440574	-0.900351	-0.090504
C	-3.099575	1.900224	-0.997125
H	-3.971687	1.296636	-1.260371
H	-2.738958	2.378837	-1.911780
H	-0.925500	-0.918615	0.629914
H	-0.071167	2.113874	-1.933741
H	-1.292638	-4.379327	-2.938570
C	-3.418131	2.918165	0.083991
H	-2.504258	3.462435	0.346363
H	-3.741736	2.391107	0.988470
C	-4.504333	3.907717	-0.355778
H	-5.411526	3.355598	-0.628427
H	-4.177771	4.426268	-1.265008
C	-4.841172	4.938987	0.724956
H	-5.615836	5.631076	0.384626
H	-5.205818	4.453338	1.635315
H	-3.961313	5.530857	0.995108
C	-1.380332	-0.958180	1.945977
H	-1.359462	0.110292	2.166344
C	-0.446633	-1.763260	2.833714
H	-0.936408	-1.870828	3.814280
H	-0.352141	-2.783327	2.449195
C	0.934611	-1.139754	3.048947
H	0.860232	-0.112381	3.416757
H	1.508369	-1.715569	3.779962
H	1.516469	-1.116256	2.124335
C	5.670273	0.365432	-0.463152
H	5.406337	-0.697353	-0.498429
H	5.599735	0.741863	-1.489644
C	7.105362	0.527960	0.053250
H	7.165886	0.158796	1.083933
H	7.359072	1.593848	0.095446
C	8.134321	-0.206092	-0.811270
H	7.928663	-1.280394	-0.844522
H	8.123111	0.165273	-1.840589
H	9.146821	-0.073502	-0.421115
